# Evaluation of a Single Dose of Ferric Carboxymaltose in Fatigued, Iron-Deficient Women – PREFER a Randomized, Placebo-Controlled Study

**DOI:** 10.1371/journal.pone.0094217

**Published:** 2014-04-21

**Authors:** Bernard Favrat, Katharina Balck, Christian Breymann, Michael Hedenus, Thomas Keller, Anna Mezzacasa, Christoph Gasche

**Affiliations:** 1 Department of Ambulatory Care and Community Medicine, University of Lausanne, Lausanne, Switzerland; 2 Private Practice for Internal Medicine, Meine, Germany; 3 Obstetric Research & Foeto-maternal Haematology Research Group, University Hospital, Zurich, Switzerland; 4 Department of Internal Medicine, Hematology Section, Sundsvall Hospital, Sundsvall, Sweden; 5 ACOMED statistic, Leipzig, Germany; 6 Vifor Pharma Ltd., Glattbrugg, Switzerland; 7 Department of Medicine 3, Medical University of Vienna, Austria; University of Florida, United States of America

## Abstract

**Background:**

Unexplained fatigue is often left untreated or treated with antidepressants. This randomized, placebo-controlled, single-blinded study evaluated the efficacy and tolerability of single-dose intravenous ferric carboxymaltose (FCM) in iron-deficient, premenopausal women with symptomatic, unexplained fatigue.

**Methods:**

Fatigued women (Piper Fatigue Scale [PFS] score ≥5) with iron deficiency (ferritin <50 µg/L and transferrin saturation <20%, or ferritin <15 µg/L) and normal or borderline hemoglobin (≥115 g/L) were enrolled in 21 sites in Austria, Germany, Sweden and Switzerland, blinded to the study drug and randomized (computer-generated randomization sequence) to a single FCM (1000 mg iron) or saline (placebo) infusion. Primary endpoint was the proportion of patients with reduced fatigue (≥1 point decrease in PFS score from baseline to Day 56).

**Results:**

The full analysis included 290 women (FCM 144, placebo 146). Fatigue was reduced in 65.3% (FCM) and 52.7% (placebo) of patients (OR 1.68, 95%CI 1.05–2.70; p = 0.03). A 50% reduction of PFS score was achieved in 33.3% FCM- vs. 16.4% placebo-treated patients (p<0.001). At Day 56, all FCM-treated patients had hemoglobin levels ≥120 g/L (vs. 87% at baseline); with placebo, the proportion decreased from 86% to 81%. Mental quality-of-life (SF-12) and the cognitive function scores improved better with FCM. ‘Power of attention’ improved better in FCM-treated patients with ferritin <15 µg/L. Treatment-emergent adverse events (placebo 114, FCM 209; most frequently headache, nasopharyngitis, pyrexia and nausea) were mainly mild or moderate.

**Conclusion:**

A single infusion of FCM improved fatigue, mental quality-of-life, cognitive function and erythropoiesis in iron-deficient women with normal or borderline hemoglobin. Although more side effects were reported compared to placebo, FCM can be an effective alternative in patients who cannot tolerate or use oral iron, the common treatment of iron deficiency. Overall, the results support the hypothesis that iron deficiency can affect women’s health, and a normal iron status should be maintained independent of hemoglobin levels.

**Trial Registration:**

ClinicalTrials.gov NCT01110356

## Introduction

Fatigue is a major health problem, affecting 14–32% of patients in primary care, particularly women [1]–[3], and often left untreated or subject to antidepressant therapy. Similar to reduced physical performance and cognitive function, fatigue is closely associated with anemia and iron deficiency (ID) [4]–[8] which are leading causes of disability as indicated by the global burden of disease study [9]. Since iron is not only a component of hemoglobin (Hb) but also a key element of various essential enzymes in all metabolic pathways (e.g. oxidative phosphorylation) [10], ID can have an Hb-independent effect on physical performance and fatigue [4],[11],[12].

A slow onset of ID-associated symptoms may lead patients to adapt to fatigue and consequently, they may not request medical help. Therefore, ID may be broadly unrecognized despite being one of the most prevalent nutrient deficiencies affecting human health and accounting for most anemia cases in underprivileged populations[13]. Even in industrialized countries a prevalence rate of 4–33% is reported, with ID being particularly common e.g. in menstruating women [14],[15].

Although previous randomized clinical trials have indicated that iron treatment (long-term oral or repeated intravenous [i.v.] iron) can reduce fatigue in iron-deficient, non-anemic (IDNA) women [5],[7],[8], the true benefit of iron supplementation in these patients is still controversial. This larger study, PREFER, evaluated the efficacy and tolerability of a single infusion of ferric carboxymaltose (FCM) in improving fatigue symptoms, quality-of-life (QoL), and cognitive function in iron-deficient, premenopausal women without active or chronic medical conditions (to exclude the mainly inflammation-related chronic fatigue syndrome). Compared to previous studies, fatigue symptoms were comprehensively assessed using the validated 22-item Piper Fatigue Scale (PFS) [16] and computerized cognitive functions test systems that are highly sensitivity for impairment or enhancement.

## Materials and Methods

The protocol for this trial and supporting CONSORT checklist are available as supporting information; see Checklist S1 and Protocol S1.

### Design and Setting

This study was designed as a randomized, placebo-controlled, single-blinded, comparative superiority study. The study was conducted from November 2010 to November 2011 at 21 sites, including private practices and general hospitals in Austria, Germany, Sweden, and Switzerland.

It was performed in compliance with the Declaration of Helsinki and the Guideline of Good Clinical Practice, registered at ClinicalTrials.gov (NCT01110356) and approved by independent ethics committees (GERMANY: Ethik-Kommission des Landes Sachsen-Anhalt (Lead EC), Ethik-Kommission der Ärztekammer Niedersachsen, Ethikkommission zur Beratung anderer berufsethischer Fragen, Ärztekammer Nordrhein - Ethik-Kommission, Ethik-Kommission der Ärztekammer Hamburg, Ethikkommission an der Medizinischen Fakultät der Universität Rostock, Ethik-Kommission Landesamt für Gesundheit und Soziales (LaGeSo), Ethik-Kommission der Ärztekammer Schleswig-Holstein; AUSTRIA: Ethik-Kommission der Medizinischen Universität Wien und des Allgemeinen Krankenhauses der Stadt Wien - AKH (Lead EC), Ethik-Kommission der Confraternität & Privatklinik Döbling; SWEDEN: Regional Ethics Review Board in Uppsala (Lead EC); SWITZERLAND: Commission Cantonale (VD) d’Ethique, Kantonale Ethikkommission Aarau, Kantonale Ethikkommission Zürich, Ethikkommission des Kantons St. Gallen, Kantonale Ethikkommission Bern). All patients gave written informed consent prior to study-related procedures.

### Study Population

Eligible patients were premenopausal, regularly menstruating women ≥18 years of age with symptomatic fatigue (≥5 points on the PFS) [16], who had ID with an unknown etiology (e.g., no menorrhagia) but had normal or borderline hemoglobin (Hb ≥115 g/L) at screening. Based on recommendations in other indications [17]–[20] and similar to the FERRIM study [5], ID was defined as serum ferritin <50 µg/L and transferrin saturation (TSAT) <20%, or ferritin <15 µg/L. Further inclusion criteria were a body weight of 50–90 kg (to exclude potential overweight-related impairment of iron metabolism), a negative pregnancy test and normal levels of C-reactive protein, thyroid-stimulating hormone, vitamin B12 and folic acid (according to each centers protocol).

Patients were excluded if they had any active or unstable concurrent medical condition, any major depressive disorder, ongoing infections or chronic inflammatory disease, any history of sleep apnea or concurrent medications that could affect physical or mental performance, a known sensitivity to any iron preparation, or use of iron preparations within 4 weeks prior screening.

### Randomization and Intervention

**Figure 1:**
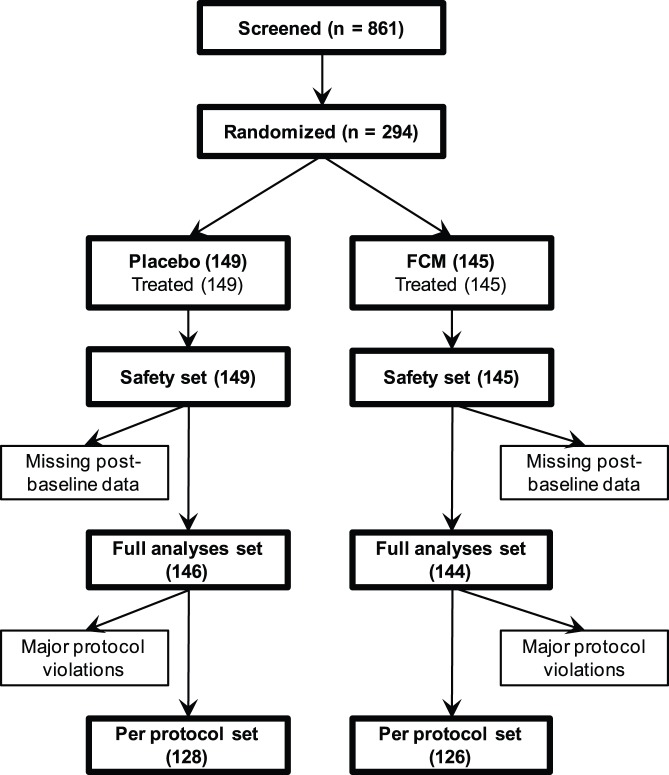
Study flow diagram. The most common major protocol deviations were ‘disallowed concurrent medications’ (15 patients in the placebo and 19 patients in the FCM group) and ‘selection criteria not met’ (5 patients in the placebo and 8 patients in the FCM group).

Baseline assessment and 1∶1 randomization to a single infusion of either FCM or placebo (Day 0) were performed within 14 days of the screening visit (patient flow in fig. 1). Treatment allocation followed a computer-generated list of random numbers that has been prepared by the clinical research organization using block randomization with variable block length (2, 4). No stratification was considered. Investigators received a set of sealed envelopes that corresponded to a randomization number and contained the identity of the study drug, and prepared and administered the study drug. Patients were blinded to the study treatment by covering infusion bags with opaque bags and using dark-colored infusion lines.

FCM (1000 mg iron) was diluted in 250 mL sterile 0.9% sodium chloride solution and administered as a single infusion over at least 15 min without the requirement for a test dose. Placebo-treated patients received 250 mL saline solution under identical conditions.

### Outcomes and Follow-up

Hematology and iron status (serum ferritin, TSAT), soluble transferrin receptor [sTfR]), were evaluated at baseline (pre-dose) and follow-up visits (Days 7, 28 and 56 or early termination visit). Blood biochemistry, including serum phosphate levels, was assessed at baseline, Day 7 and Day 56. Fatigue symptoms and treatment efficacy were assessed by the 22-item PFS [16] at each visit (classification of severity: 1–3 none or mild, 4–6 moderate, 7–10 severe). Additional efficacy measures were the SF-12 QoL questionnaire [21] (baseline and Day 56), and computerized cognitive tests, performed at each visit with the CDR system [22] (assessing attention, concentration and short-term memory) and the Bond-Lader visual analogue scale (VAS) (self-rated assessment of alertness, contentment and calmness) [23].

The primary endpoint was defined as the proportion of patients who achieved a ≥1 point decrease in total PFS score from baseline to Day 56. Secondary endpoints included mean changes from baseline to Day 56 in PFS total and subscale scores, the SF-12 QoL questionnaire [21] and cognitive function tests. Furthermore, the proportion of patients with Hb ≥120 g/L, ferritin ≥50 µg/L and TSAT ≥20% at Day 56 as well as the relationship between the change in ferritin and the success rate in cognitive tests were assessed. An additional objective that was defined before analysis of the data was the proportion of patients with 50% PFS-reduction.

Safety endpoints, including frequency of adverse events (AEs) and abnormal laboratory parameters, were evaluated at each visit. AEs that began or worsened in severity after study drug administration were considered treatment-emergent (TEAEs). Intensity/severity of an AE was categorized as ‘mild’, ‘moderate’ or ‘severe’ by the investigators. Events were categorized ‘related’ or ‘unrelated’ to the study drug (binary scale).

### Statistical Analysis

Sample size estimation (288 patients; 144 per group) was based on a two-group χ^2^ test with a 0.050 two-sided significance level, 80% power, a 10% drop-out rate, and a 17% minimum difference of responders between groups (calculated from response rates observed in a related subgroup in the FERRIM trial [5]).

The analysis populations comprised the safety set (SAF; all randomized patients who had received study treatment), the intent to treat set (ITT; treated patients with available post-baseline efficacy data) and the per-protocol set (PP; patients with available values of the relevant study variables at all periods and no major protocol violation).

Descriptive statistics of continuous data comprised mean±SD (standard deviation), median with interquartile range, and between-group difference with 95%CI (confidence interval). The primary objective was analyzed via two-sided χ^2^-test (alpha-level: 0.05). Changes in outcome measures were analyzed by mixed model repeated ANOVA (PROC MIXED in SAS). SF-12 was analyzed using a t-test. Single missing data were imputed using the last observation carried forward rule. Derived variables were calculated afterwards.

## Results

### Patient Characteristics

Of 861 screened patients, 294 fulfilled the inclusion-criteria and were randomized and treated (SAF: 145 to FCM, 149 to placebo). Of those, 290 patients were included in the ITT (FCM: 144; placebo: 146; fig. 1). The mean age was 35 years. Baseline clinical and laboratory characteristics were similar for the two treatment groups (table 1). Hb levels <120 g/L were measured in 19 [13.1%] patients of the FCM and 21 [14.1%] of the placebo group. Most enrolled patients (283 [96.3%]) completed the trial (FCM: 142 [97.9%]; placebo: 141 [94.6%]). Eleven patients withdrew prematurely, with “lost to follow-up” (FCM: 2 patients; placebo: 4 patients) being the most common reason. Two placebo-treated patients discontinued due to lack of efficacy.

**Table 1:** Patient baseline characteristics, SAF.

Mean (SD), median (Q1, Q3)*	FCM (N = 145)	Placebo (N = 149)
Age [years]	34.6 (8.8)	35.0 (9.6)
Weight [kg]*	64.0 (57.0, 72.5)	63.0 (58.4, 75.4)
PFS*	6.4 (5.7, 7.2)	6.4 (5.5, 7.3)
SF12 (Mental Score)	41.2 (9.7)	42.3 (9.2)
SF12 (Physical Score)	44.5 (8.2)	43.8 (8.1)
Hb [g/L]*	128 (124, 135)	129 (122, 134)
MCV [fL]*	87.4 (84.1, 90.0)	87.0 (85.0, 91.0)
Serum ferritin [µg/L]*	15 (10, 25)	16 (11, 28)
TSAT [%]*	14.0 (10.0, 18.0)	14.4 (10.9, 18.5)
CRP [mg/L]*	2.0 (1.0, 3.1)	1.7 (1.0, 3.1)

*Weight, PFS, Hb, MCV, serum ferritin, TSAT and CRP shown as median (Q1, Q3).

CRP C-reactive protein; Hb hemoglobin; MCV mean corpuscular volume; PFS piper fatigue scale; TSAT transferrin saturation.

### Fatigue Improvement

**Figure 2:**
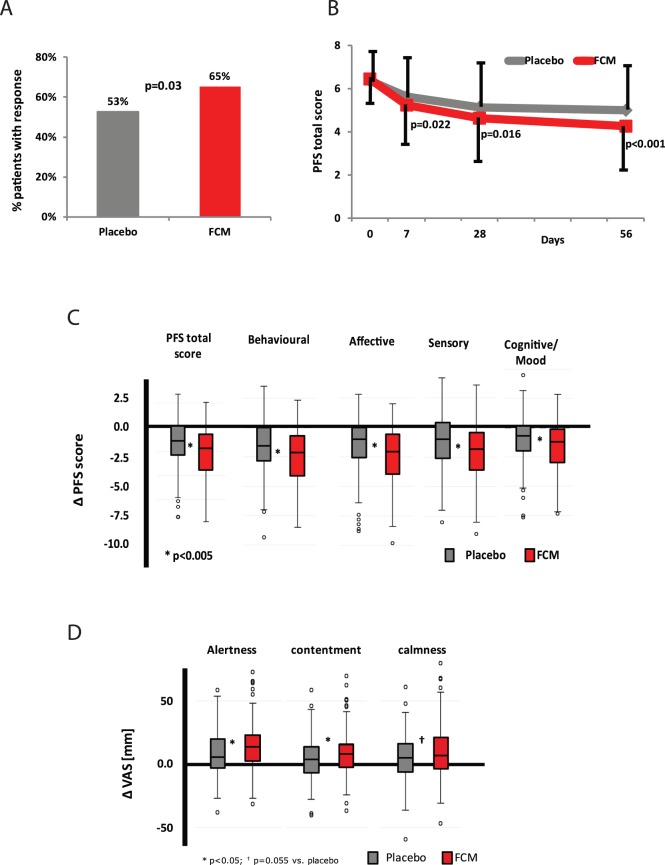
Treatment effects of FCM vs. placebo in fatigued, iron-deficient, non-anemic women (ITT; error bars SD). (A) Proportion of patients with ≥1 point reduction in PFS (primary endpoint). (B) Improvement of mean PFS total score in FCM- vs. placebo-treated patients throughout the study (Mean differences ± SD vs. baseline: Day 7: −1.2±1.8 vs. −0.8±1.5 points; Day 28: −1.8±2.1 vs. 1.2±2.0 points; Day 56: −2.2±2.1 vs. −1.4±2.0 points). P-values given for intergroup differences. (C) Mean change (Δ) in PFS total score and subscale scores from baseline to Day 56. *P≤0.01 for all scales. (D) Mean change (Δ) in self-rated alertness, contentment and calmness (computer-based VAS scales). *P<0.05; †P = 0.055 vs. placebo.

The percentage of patients with improved total PFS score (≥1 point decrease from baseline to Day 56) was statistically significantly higher in the FCM arm (65.3%) than in the placebo arm (52.7%) (difference: 12.5% [1.3% to 23.8%]; number needed to treat [NNT] = 8.0; P = 0.03; ITT; table 2, fig. 2A). A 50%-reduction of PFS total score at Day 56 was achieved by 33.3% FCM- vs. 16.4% placebo-treated patients (difference: 16.9% [7.1% to 26.7%]; NNT = 5.9; P<0.001). The latter treatment effect was also significant in the subpopulation of patients with baseline Hb ≥120 g/L (FCM: 29.6%; placebo: 16.8%; difference 12.8%; NNT = 7.7; P = 0.016). A lower mean total PFS score in the FCM vs. the placebo group was already observed after one week (fig. 2B). At Day 56, improvement in all PFS subscores was significantly greater in FCM-treated patients (table 3, fig. 2C). In the PP, the results were even more in favor of the FCM group (primary endpoint: difference 14.3% [2.5% to 26.2%], P = 0.019).

**Table 2:** Primary endpoint – Fatigue (% [n]) (ITT).

	FCM (N = 144)	Placebo (N = 146)	Difference [95% CI] FCM–placebo on Day 56, P-value
**≥1 point decrease in total PFS**	65.3 (94)	52.7 (77)	12.5 [1.3, 23.8], P = 0.030

PFS Piper fatigue scale.

**Table 3:** Secondary endpoints – Fatigue, QoL and cognitive function tests (mean [SD]) (ITT).

	FCM (N = 144)		Placebo (N = 146)		
	Baseline	Day 56	Baseline	Day 56	Difference [95% CI] in changesfrom baseline to Day 56, P-value
**PFS total score** †	6.46 (1.16)	4.26 (2.08)	6.37 (1.33)	5.01 (2.02)	−0.80 [−1.26, −0.34], P<0.001
**PFS behavioral severity** †	6.45 (1.44)	4.00 (2.33)	6.33 (1.56)	4.77 (2.27)	−0.86 [−1.36, −0.35], P<0.001
**PFS affective meaning** †	7.23 (1.27)	4.71 (2.48)	7.00 (1.53)	5.44 (2.31)	−0.89 [−1.41, −0.37], P<0.001
**PFS sensory** †	6.74 (1.33)	4.60 (2.21)	6.67 (1.56)	5.29 (2.15)	−0.72 [−1.21, −0.23], P = 0.004
**PFS cognitive/mood** †	5.59 (1.41)	3.88 (1.82)	5.63 (1.51)	4.65 (1.87)	−0.75 [−1.16, −0.34], P<0.001
**SF-12 mental health** ‡	41.3 (9.7)	47.3 (8.7)	42.2 (9.2)	45.1 (9.1)	3.0 [0.9, 5.2], P = 0.007
**SF-12 physical health** ‡	44.5 (8.2)	49.1 (7.4)	43.9 (8.2)	46.7 (7.7)	1.7 [−0.1, 3.6], P = 0.067
**VAS alertness** ‡	46.5 (12.7)	60.7 (17.5)	46.9 (13.8)	55.6 (17.1)	5.16 [1.33, 8.98], P = 0.008
**VAS contentment** ‡	60.6 (13.9)	69.8 (16.23)	61.2 (14.6)	65.7 (15.3)	4.37 [0.98, 7.76], P = 0.012
**VAS calmness** ‡	56.5 (16.1)	66.6 (17.6)	58.7 (16.0)	63.6 (15.1)	3.64 [−0.08, 7.35], P = 0.055
**CFT power of attention [msec]** †	1146 (127)	1165 (129)	1162 (122)	1190 (136)	−11.7 [−30.6, 7.3], P = 0.23
**CFT continuity of attention** ‡	91.6 (2.6)	91.9 (2.9)	91.2 (4.1)	91.0 (4.8)	0.64 [−0.10, 1.39], P = 0.09
**CFT quality of working memory** ‡	1.85 (0.22)	1.87 (0.19)	1.86 (0.22)	1.80 (0.35)	0.054 [−0.004, 0.113], P = 0.07
**CFT quality of episodic memory** ‡	221 (56)	243 (57)	219 (51)	240 (52)	2.65 [−7.20, 12.50], P = 0.60
**CFT speed of memory [msec]** †	3187 (609)	2944 (502)	3191 (624)	3008 (491)	−55.5 [−123.9, 12.8], P = 0.11

† A negative value of the difference FCM–placebo is in favor of FCM;

‡ A positive value of the difference FCM–placebo is in favor of FCM.

PFS Piper fatigue scale; QoL quality-of-life; SF short form; VAS visual analogue scale (results in mm); CFT cognitive function test (results in msec).

### Mental and Physical Quality-of-life

Mean improvement in the SF-12 mental health summary from baseline to Day 56 was better in the FCM group compared to placebo (P = 0.007; table 3). Improvement in physical health was numerically, but not statistically significantly, better in FCM-treated patients (table 3).

### Cognitive Function

A mixed model analysis of changes in VAS scores showed that FCM treatment led to significantly better improvement in alertness compared to placebo from Day 7 onwards (difference: 4.30 [0.73 to 7.88]; P = 0.019). At Day 56, FCM-associated improvement was significantly better for contentment and showed a trend for calmness (table 3, fig. 2D).

Changes in cognitive function subscales were numerically larger with FCM but not significantly different to placebo. Among patients with ferritin <15 µg/L, an FCM-associated significantly better improvement in ‘power of attention’ was seen at Day 56 (difference FCM vs. placebo: −38.4 msec [−65.8 to −11.0]; P = 0.006).

### Hematologic Response and Iron Status

**Figure 3:**
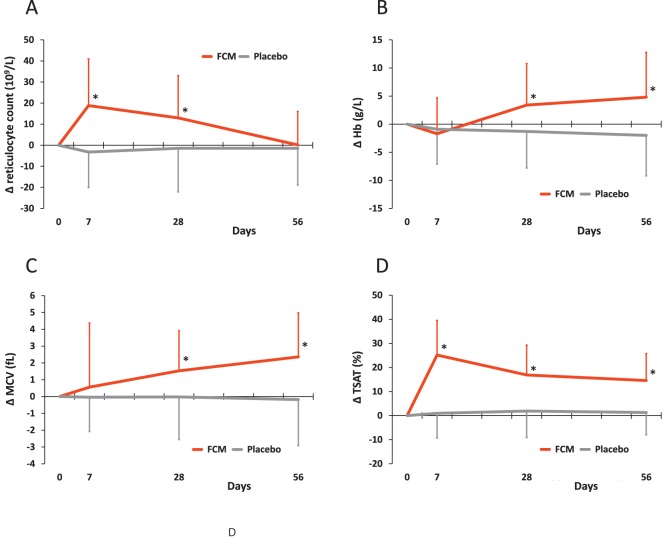
Mean changes (Δ) in reticulocyte counts, Hb levels, MCV and TSAT (error bars SD). The rapid increase of reticulocyte counts in the FCM group is in line with early FCM-associated improvement of the fatigue total score (fig. 2B). The significantly higher increase in reticulocyte counts in the FCM group at Days 7 and 28 (A) translated into higher increases of Hb and MCV at Days 28 and 56 (B,C). The early FCM-associated increase in reticulocyte counts remained significantly higher compared to placebo when analysing the subgroup of patients with Hb≥120 g/L (P<0.001 for Days 7 and 28). This suggests that iron deficiency without anaemia affected erythropoiesis in these apparently healthy women and raises doubts whether the currently recommended Hb cut-off level of 120 g/L to diagnose anaemia in premenopausal, menstruating women is appropriate. TSAT remained significantly improved until the end of the 56-day study period (D). *P<0.0001 for difference between FCM and placebo group in reticulocyte counts and Hb levels, respectively.

At Day 56, all FCM-treated patients achieved Hb levels ≥120 g/L (mean [min, max] 134 g/L [120, 159]) whereas 19.2% of placebo-treated patients had lower Hb levels (127 g/L [89, 154]; P<0.001; table 4). Mean reticulocyte count increased transiently in FCM-treated patients at Days 7–28 followed by an increase in Hb and mean corpuscular volume (MCV) from Day 28 onwards (fig. 3A–C). The majority of FCM-treated patients achieved normal iron status (ferritin, TSAT) at Day 56, whereas most placebo-treated patients had subnormal iron indices (table 4, fig. 3D). Mean levels of sTfR were significantly lower in FCM vs. placebo-treated patients at all time-points (Day 7: −0.32±0.32 vs. 0.02±0.27 mg/L; Day 28: −0.37±0.42 vs. 0.07±0.42 mg/L; Day 56: −0.56±0.51 vs. 0.13±0.61 mg/L; all time-points P<0.001). Among FCM-treated patients, the extent of changes in ferritin did not correlate with changes in other endpoints. An additional analysis including the patients of both groups (FCM and placebo) confirmed that the increase in ferritin correlated with improvement in the fatigue score (Spearman correlation coefficient, r = −0.173; P = 0.003).

**Table 4:** Secondary endpoints – Hematologic parameters (% [n] or mean [SD]) (ITT).

	FCM (N = 144)		Placebo (N = 146)		
	Baseline	Day 56	Baseline	Day 56	Difference [95% CI] FCM–placebo on Day 56, P-value
**Hb ≥120 g/L** ‡ **^,^** §	86.8 (125)	100 (144)	85.6 (125)	80.8 (118)	19.2 [12.8, 25.6], P<0.001
**Serum ferritin ≥50 µg/L** ‡ **^,^** §	0.7 (1)	99.3 (143)	1.4 (2)	2.1 (3)	97.3 [94.6, 99.9], P<0.001
**TSAT ≥20%** ‡ **^,^** §	9.9 (14)	81.3 (117)	13.8 (20)	32.9 (48)	48.4 [38.4, 58.3], P<0.001
**MCV (fl)** ‡ **^,^** §	87.3 (4.5)	89.6 (3.9)	87.4 (4.4)	87.2 (4.8)	2.41 [1.39, 3.42], P<0.0001

‡ A positive value of the difference FCM–placebo is in favor of FCM;

§ Mean Hb levels at Day 56 were 134±7.7 g/L (FCM) vs. 127±10.1 g/L (placebo), median serum ferritin levels at Day 56 were 169 µg/L (FCM) vs. 16 µg/L (placebo) and median TSAT was 27% (FCM) vs. 15% (placebo).

Hb hemoglobin; TSAT transferrin saturation; MCV mean corpuscular volume.

### Tolerability and Biochemical Analyses

TEAEs were experienced by 57.2% of FCM- and 49.0% of placebo-treated patients (P = 0.16, not significant at alpha = 0.05 level; table 5). The most frequently reported TEAEs were headache, nasopharyngitis, pyrexia and nausea. Overall, TEAS were mainly mild (66.5% vs. 63.2%) or moderate (28.2% vs. 36.8%) in intensity. The frequency of infections and infection-like symptoms was similar in the FCM and placebo group (18.6% vs. 20.1%: P = 0.74). Among 66 events of infections and infestations in 57 (19.4%) patients, nasopharyngitis (21 in 21 patients), respiratory tract infections (6 in 6 patients) and sinusitis (5 in 5 patients) occurred most frequently and without significant difference between the treatment groups. The TEAEs by system organ class that were reported more frequently in the FCM group were general disorders and administration site conditions (16.6% vs. 2.7%; P<0.05), nervous system disorders (20.0% vs. 12.1%; P = 0.06) and gastrointestinal disorders (11.7% vs. 6.0%; P = 0.09). Five FCM-treated patients reported severe TEAEs; in four, the events were considered drug-related (one patient: nausea, headache, heavy legs, arthralgia, myalgia; one patient: hematoma and 2x discoloration at injection sites; one patient: headache; one patient: nightly sweat). An additional covariate analysis indicated that relatively young age and low body weight were independent risk factors for all and related TEAEs, with age being the more relevant factor. Patients younger than 34 years (study population’s median age, n = 69) experienced more often at least one TEAE or related TEAE than patients that were older (n = 76) (68.8% vs. 51.3%; P<0.0001 or 37.7% vs. 19.7%; P<0.0001, respectively). Among patients with a body weight ≤65 kg (n = 79) and a weight >65 kg (n = 66), similar proportions had at least one TEAE (55.7% vs. 59.1%) but more patients with a body weight ≤65 kg had a related TEAE (36.7% vs. 18.2%).

**Table 5:** Adverse events profile (SAF).

	FCM (N = 145) % (n), events	Placebo (N = 149) % (n), events	P-value*
**Any TEAE (total)**	57.2% (83), 209	49.0% (73), 114	n.s.
– related†	28.3% (41), 111	3.4% (5), 6	P<0.0001
**Any mild or moderate TEAE**	53.8% (78), 198	49.0% (73), 114	n.s.
– related†	25.5% (37), 101	3.4% (5), 6	P<0.0001
**Any severe TEAE**	3.4% (5), 11	0	P = 0.03*
– related†	2.8% (4), 10	0	P = 0.06*
**Any SAE**	0.7% (1), 1	0	n.s.
– related†	0	0	n.s.
**Most common TEAEs (in ≥5% of patients):**			
– Headache	15.9% (23), 30	10.7% (16), 18	n.s.
– Nasopharyngitis	9.0% (13), 13	5.4% (8), 8	n.s.
– Pyrexia	8.3% (12), 13	1.3% (2), 2	p = 0.005
– Nausea	5.5% (8), 11	1.3% (2), 2	p = 0.06*

*p-values calculated by χ^2^-test except for severe TEAEs and nausea (Fisher’s exact test);

† TEAEs with a temporal relationship to the study drug administration if no other drug, therapeutic intervention or underlying condition provides a sufficient explanation for the observed event.

Three mild cases of urticaria that were considered FCM-related resolved without medication. Among the five patients that experienced one or more severe TEAEs, one patient had nausea, heavy legs, headache, arthralgia and myalgia, one patient had hematoma and two times discoloration at injection sites, and three patients had one severe TEAE each (headache, cough and night sweat, respectively).

TEAE treatment emergent adverse event; SAE serious adverse event; n.s. not significant.

No drug-related severe or serious allergic reactions were observed. One moderate hypersensitivity reaction after FCM infusion that resolved on the same day was reported in a patient with a history of hypersensitivity.

Most FCM-treated patients had a clinically asymptomatic, transient decrease in serum phosphate below normal (0.8 mmol/L) at Day 7 (86% [FCM] vs. 2% [placebo]; 0.62±0.41 vs. 1.12±0.18 mmol/L) that resolved spontaneously until end-of-study in almost all FCM-treated patients (91.9%; 1.09±0.24 vs. 1.17±0.30 mmol/L [FCM vs. placebo]). No other clinically meaningful between-group differences in laboratory parameters were observed. One treatment-unrelated serious AE has been reported in the FCM group (moderate left thoracic pain). No deaths or AE-related premature discontinuation of the study drug were reported.

## Discussion

So far, there was only limited clinical evidence that ID without anemia may cause chronic fatigue and impair QoL in menstruating women that appear to be healthy [5],[7],[8]. This study, PREFER, demonstrates that iron repletion with a single FCM-infusion (1000 mg iron) effectively and quickly reduced fatigue symptoms, and improved patient-rated mental QoL, cognitive functions and erythropoiesis in iron-deficient women with normal or borderline hemoglobin levels.

Three randomized controlled studies have reported that long-term oral iron can improve fatigue in IDNA women [7],[8],[24]. However, oral iron has limitations such as long time to response (particularly in case of recurrent blood loss as in menstruating women), gastrointestinal side effects, low adherence to therapy, and lack of QoL improvement [8],[25]. Notably, constipation and blackened stool may have compromised patient blinding in two studies [7],[8] resulting in higher reported response rates in the treatment group and lower placebo-effects. One randomized, controlled pilot study on repeated i.v. iron sucrose administration in IDNA women showed a trend that 4×200 mg iron over two weeks may reduce fatigue [5]. While the study’s primary endpoint (decrease in fatigue 6 weeks after treatment initiation) did not reach statistical significance vs. placebo, post-hoc analyses demonstrated a significant effect on fatigue.

In our study the 22-item PFS [16], the SF-12 health-related short form and cognitive function tests were used to evaluate and monitor patients’ fatigue and overall condition. Currently, no target levels or improvements are defined for fatigue. However, improvement of fatigue by a ≥1 point decrease in PFS score (primary endpoint) is considered clinically relevant [26] and corresponds to a ≥10% improvement on this scale, which is similar to the previously reported 0.97 point mean decrease on a 10-point VAS [7]. The early response, from Day 7 onwards, comprises a substantial improvement in time-to-response and patient convenience, and emphasizes the clinical relevance of the FCM treatment. Numbers-needed-to-treat (5.9 for 50% fatigue reduction and 8.0 for ≥1-point decrease of PFS total score) are comparable to those for psychological treatments of adult depression [27].

The strength of these results is supported by the consistent improvement in all PFS subscores and mental QoL (SF-12). The mental score reached by the FCM group is comparable to the norm value assessed for 35-year-old women in a general population (48±10 points) [28]. Furthermore, the improvements in self-rated alertness, contentment and calmness are an additional, independent assessment of patients and support the findings for PFS and SF-12.

Changes in the cognitive function test subscales did not reach statistical significance in the overall trial population. However, in patients with ferritin <15 µg/L, a significant difference in ‘power of attention’ was observed in FCM- vs. placebo-treated patients. The 43 msec improvement in power of attention compares to differences between cancer patients with ECOG (Eastern Cooperative Oncology Group) score zero (fully active) and one (can work but restricted activity) for whom medical fatigue treatment is more common [29]. In line with the observed treatment effect in FCM- vs. placebo-treated patients, the improvement in fatigue and cognitive function scores correlated with the increase in ferritin levels. Within the group of FCM-treated patients; however, the magnitude of changes in ferritin levels did not predict or correlate with changes in the endpoint scores. Also prior trials did not show a correlation between changes in serum ferritin and fatigue improvement [7],[8]. However, recent improvements in imaging techniques [30] may allow monitoring of the cerebral iron status and its potential correlation with fatigue in future trials.

Screening failures were not systematically recorded, yet a TSAT ≥20% was the main reason. This confirms that not every fatigued patient is automatically iron-deficient and appropriate iron status assessment is mandatory before initiating any kind of iron therapy.

The selection of cut-off levels defining iron deficiency anemia often raises controversies. Despite the WHO’s definition of non-anemic as Hb ≥120 g/L, the cut-off for inclusion in this study was set slightly lower (Hb ≥115 g/L) in order to include women with a borderline Hb that might have resulted from physiological fluctuations in this population (e.g. a transient drop in Hb after menstruation). The selected cut-off is in line with guidelines for the management of heavy menstrual bleeding [31] (approved and endorsed by the Royal New Zealand College of General Practitioners) and some textbooks [32],[33]. Also one of the prior studies investigating oral iron in fatigued women used a lower than the WHO-recommended cut-off (≥117 g/L) [7]. The serum ferritin cut-off is in line with prior studies on iron-based fatigue treatment [5],[8] but lower compared to conditions associated with chronic inflammation. A TSAT <20% is a widely accepted definition of insufficiently available iron and iron-restricted erythropoiesis [17],[20].

The PFS response in women with Hb ≥120 g/L confirmed the benefit of FCM in fatigue improvement among women that are non-anemic according to the WHO definition. This is in line with results in iron-deficient chronic heart failure patients showing significant improvements in cardiac functional class and physical performance in FCM- compared to placebo-treated patients regardless whether they were anemic or non-anaemic [34].

In contrast to the placebo group, all FCM-treated patients achieved or maintained Hb levels ≥120 g/L at Day 56. The transient increase in reticulocyte counts at Days 7 and 28 (statistically significantly better with FCM than placebo in both the overall trial population and the subgroup with Hb ≥120 g/L) together with the decrease in sTfR in FCM-treated patients indicates that FCM-based iron repletion enhanced erythropoiesis.

Notably, the PREFER study was the first trial investigating the safety and efficacy of FCM in fatigued but otherwise apparently healthy subjects. Together with the quite young age of the study population, this may have led to an overly cautious adverse event reporting rate also known as nocebo effect and suggested by the unusually high TEAE rate in the placebo arm. Accordingly, the frequency of FCM-related TEAEs (28%; all known side effects of FCM) seems to be higher than in other studies and associated with patients <34 years of age [34]–[37]. The binary causality assessment (related/unrelated) together with the single-blinded design may have added to over-reporting of related events compared to studies using the four-graded WHO causality scale [38] or double-blinding [35],[36]. Since the proportions of patients with at least one TEAE were not significantly different between those with a body weight below or above 65 kg, FCM doses of 1000 mg iron (max. 20 mg/kg) should be considered for iron-deficient patients who may benefit from i.v. iron treatment. Patients with a body weight below 65 kg who might be concerned about a potential risk of treatment-related symptoms, may be scheduled for two administrations of 500 mg iron with a one-week interval.

In contrast to the controversial discussion about a potential impact of i.v. iron on immune function [39], no difference in the frequency of infections was observed between the FCM and the placebo group. Similar to observations in earlier studies [36],[37],[40]–[42], the transient decrease in serum phosphate levels was asymptomatic and did not require treatment. The observation of only one moderate and spontaneously resolving hypersensitivity reaction is in line with prior experience that the dextran-free compound FCM exerts only a very low risk of hypersensitivity reactions [43]. Also a recent review of the risk of allergic reactions by the European Medicines Agency (EMA) concluded that the benefits of i.v. iron outweigh the risks in the treatment of ID. Accordingly, no test dose is needed although availability of resuscitation facilities is still required [44].

Since controlled studies of FCM in different patient populations confirmed a positive benefit/risk ratio for treatment with a single FCM dose of 1000 mg iron [34],[37],[45], and a prior pilot study in a similar population suggested a beneficial effect of iron sucrose given at a total dose of 800 mg iron, the approved FCM treatment with 1000 mg as a single dose was selected for this study.

Although ID is the major cause of fatigue, a substantial placebo-effect has been observed in this and other studies [5]. This may be associated with the emotional component of fatigue and the i.v. administration route. Notably, placebo-treated patients showed only modest fatigue reduction after the initial decrease, whereas the mean PFS of FCM-treated patients continuously decreased until Day 56 after the rapid initial reduction. Substantial placebo effects have also been reported in prior fatigue studies [5],[7] as well as for the management of another patient-reported outcome such as chronic low back pain [46]. The impact of patient expectations, associative learning and cognitive factors on the placebo response across different diseases and physiological systems which may trigger even complex neurobiological phenomena has been described before [47].

In clinical practice, active women may prefer i.v. iron due to its rapid effect, few adverse events and the single administration compared with up to three times daily oral medication. Although the single FCM administration can effectively replenish iron stores, the iron status (i.e. ferritin levels and % TSAT) should reassessed after a certain period (e.g. three months) to evaluate whether re-treatment is necessary. Since i.v. administration requires medical expertise it may be more expensive than oral iron treatment. While economic studies have shown that FCM may reduce the costs of ID treatment compared to other i.v. iron preparations [37],[48], a similar cost analysis vs. oral iron has not yet been performed.

## Conclusions

The results of this placebo-controlled study show the effectiveness of a single ferric carboxymaltose dose to rapidly reduce fatigue and to improve cognitive function scores in iron-deficient women with normal to borderline Hb. All FCM-treated patients achieved or maintained a normal Hb level. As with most placebo-controlled studies, more side effects were reported with the active study treatment. Although oral iron will remain the common treatment of iron deficiency in practice, the results of this study confirm that i.v. ferric carboxymaltose can serve as an effective alternative if oral iron is not tolerated or cannot be used. Overall, the results favor the maintenance of a normal iron status independent of Hb levels. The assessment and treatment of ID in women with unexplained fatigue should prevent incorrect diagnosis and might reduce inappropriate medication use.

## Supporting Information

Checklist S1 **CONSORT Checklist.**
(PDF)

Investigatorlist S1 **List of local principal investigators and institutions that participated in this study.**
(PDF)

Protocol S1 **Trial Protocol.**
(PDF)
