# Supplementary material for: Evaluation of a Single Dose of Ferric Carboxymaltose in Fatigued, Iron-Deficient Women – PREFER a Randomized, Placebo-Controlled Study
Source: PLoS One. 2014 Apr 21;9(4):e94217. doi: 10.1371/journal.pone.0094217 (PMC3994001; doi:10.1371/journal.pone.0094217)
Supplement: Checklist S1 — CONSORT Checklist. (PDF) [file pone.0094217.s001.pdf]

CONSORT 2010 checklist of information to include when reporting a randomised trial*

Item No	Checklist item	Reported in section(s)
1a	Identification as a randomised trial in the title	Title, Methods (Design and setting)
1b	Structured summary of trial design, methods, results, and conclusions (for specific guidance see CONSORT for abstracts)	Abstract
2a	Scientific background and explanation of rationale	Introduction
2b	Specific objectives or hypotheses	Introduction
3a	Description of trial design (such as parallel, factorial) including allocation ratio	Methods (Design and setting, Randomization and intervention)
3b	Important changes to methods after trial commencement (such as eligibility criteria), with reasons	n.a.
4a	Eligibility criteria for participants	Methods (Study population)
4b	Settings and locations where the data were collected	Methods (Design and setting)
5	The interventions for each group with sufficient details to allow replication, including how and when they were actually administered	Methods (Randomization and intervention)
6a	Completely defined pre-specified primary and secondary outcome measures, including how and when they were assessed	Methods (Outcomes and follow-up)
6b	Any changes to trial outcomes after the trial commenced, with reasons	n.a.
7a	How sample size was determined	Methods (Statistical analysis)
7b	When applicable, explanation of any interim analyses and stopping guidelines	n.a.
8a	Method used to generate the random allocation sequence	Methods (Randomization and intervention)
8b	Type of randomisation; details of any restriction (such as blocking and block size)	Methods (Randomization and intervention)
9	Mechanism used to implement the random allocation sequence (such as sequentially numbered containers), describing any steps taken to conceal the sequence until interventions were assigned	Methods (Randomization and intervention)
10	Who generated the random allocation sequence, who enrolled participants, and who assigned participants to interventions	Methods (Randomization and intervention)
11a	If done, who was blinded after assignment to interventions (for example, participants, care providers, those assessing outcomes) and how	Methods (Randomization and intervention)
11b	If relevant, description of the similarity of interventions	n.a.
12a	Statistical methods used to compare groups for primary and secondary outcomes	Methods (Statistical analysis)

12b	Methods for additional analyses, such as subgroup analyses and adjusted analyses	n.a.
13a	For each group, the numbers of participants who were randomly assigned, received intended treatment, and were analysed for the primary outcome	Results (Patient characteristics), Figure 1
13b	For each group, losses and exclusions after randomisation, together with reasons	Results (Patient characteristics), Figure 1
14a	Dates defining the periods of recruitment and follow-up	Methods (Design and setting)
14b	Why the trial ended or was stopped	n.a.
15	A table showing baseline demographic and clinical characteristics for each group	Results (Patient characteristics), Table 1
16	For each group, number of participants (denominator) included in each analysis and whether the analysis was by original assigned groups	Results (Patient characteristics)
17a	For each primary and secondary outcome, results for each group, and the estimated effect size and its precision (such as 95% confidence interval)	Results (Fatigue improvement, Mental and physical quality-of-life, Cognitive function, Hematologic response and iron status, Tolerability and biochemical analyses), Table 2, Figure 2, Figure 3
17b	For binary outcomes, presentation of both absolute and relative effect sizes is recommended	n.a.
18	Results of any other analyses performed, including subgroup analyses and adjusted analyses, distinguishing pre-specified from exploratory	Results (Cognitive function), Figure 3
19	All important harms or unintended effects in each group (for specific guidance see CONSORT for harms)	Results (Tolerability and biochemical analyses), Table 3
20	Trial limitations, addressing sources of potential bias, imprecision, and, if relevant, multiplicity of analyses	Discussion
21	Generalisability (external validity, applicability) of the trial findings	Discussion
22	Interpretation consistent with results, balancing benefits and harms, and considering other relevant evidence	Discussion
23	Registration number and name of trial registry	Methods (Design and setting)
24	Where the full trial protocol can be accessed, if available	n.a.
25	Sources of funding and other support (such as supply of drugs), role of funders	Online Editorial Manager

*We strongly recommend reading this statement in conjunction with the CONSORT 2010 Explanation and Elaboration for important clarifications on all the items. If relevant, we also recommend reading CONSORT extensions for cluster randomised trials, non-inferiority and equivalence trials, non-pharmacological treatments, herbal interventions, and pragmatic trials. Additional extensions are forthcoming: for those and for up to date references relevant to this checklist, see www.consort-statement.org.
