# Supplementary material for: Evaluation of a Single Dose of Ferric Carboxymaltose in Fatigued, Iron-Deficient Women – PREFER a Randomized, Placebo-Controlled Study
Source: PLoS One. 2014 Apr 21;9(4):e94217. doi: 10.1371/journal.pone.0094217 (PMC3994001; doi:10.1371/journal.pone.0094217)
Supplement: Investigatorlist S1 — List of local principal investigators and institutions that participated in this study. (PDF) [file pone.0094217.s002.pdf]

The following institutions and local principal investigators participated in this study:

Austria: Hans Christian Egarter, Universitätsklinik für Frauenheilkunde, Vienna; Christoph Gasche, Loha for Life, Vienna

Germany: Hans Joachim Ahrendt, Praxis für Frauenheilkunde, Magdeburg; Katharina Balck, Praxis Dr. Balck, Meine; Claudia Büttner, Klinische Forschung Berlin-Mitte GmbH, Berlin; Lothar Imhof, Praxis Dr. med. Imhof, Ahrensburg; Susanne Mindt-Prüfert, Klinische Forschung Hamburg GmbH, Hamburg; Sabine Mucha, Gemeinschaftspraxis Drs. Mucha, Schalk & Görtz-Diamadis, Wuppertal; Christiane Paschen, Klinische Forschung Schwerin GmbH, Schwerin; Veronika Richter, Klinische Forschung Hannover-Mitte GmbH, Hannover; Dirk Züchner, Klinische Forschung Berlin-Buch GmbH, Berlin

Sweden: Bo Anzén, Kvinnoklinikens forskningsmottagning, Stockholm; Michael Hedenus, Sundsvalls Sjukhus, Sundsvall; Lena Leissner, Neurologkliniken, Sömnenheten, Örebro; Jan Wesström, Kvinnokliniken, S-MVC, Falun; Ronnie Willenheimer, Hjärtkärl-Kliniken i Malmö, Limhamn

Switzerland: Bernard Favrat, Department of Ambulatory Care and Community Medicine, University of Lausanne, Lausanne; Andreas Huber, Kantonsspital Aarau AG, Aarau; Alexander Krafft, Universitätsspital Zürich, Zürich; Bernd Schultes, Interdisziplinäres Adipositas-Zentrum, Rorschach; Petra Stute, Universitätsklinik für Frauenheilkunde, Bern
