# Supplementary material for: Evaluation of a Single Dose of Ferric Carboxymaltose in Fatigued, Iron-Deficient Women – PREFER a Randomized, Placebo-Controlled Study
Source: PLoS One. 2014 Apr 21;9(4):e94217. doi: 10.1371/journal.pone.0094217 (PMC3994001; doi:10.1371/journal.pone.0094217)
Supplement: Protocol S1 — Trial Protocol. (PDF) [file pone.0094217.s003.pdf]

To whom it may concern,

This supplement contains the following item:

Original/Final protocol of the PREFER study

No amendments of the original protocol have been performed

A Multicentre Randomised Placebo-controlled Study to Assess the Efficacy and Safety of a Single Administration of Ferric Carboxymaltose (1,000 mg iron) in Improving Fatigue Symptoms in Iron-deficient Non-anaemic (IDNA) Women of Child Bearing Age

Clinical Protocol Number:	IDNA 2009-01
Date:	25 November 2009
Prior Amendments:	N/A
Sponsor Contact:	Dr Lise Riopel, PhD
Sponsor Address:	Vifor Pharma<br>Flughofstrasse 61<br>Glattbrugg CH-8152<br>Switzerland
Monitor Details:	SGS Life Sciences Services<br>Generaal De Wittelaan 19A bus 5<br>B-2800 Mechelen<br>Belgium
Principal Investigator:	PD Dr. B. Favrat, MD
Project Statistician:	Dr Thomas Keller, PhD<br>ACOMED statistik<br>Fockestr. 57<br>D 04275 Leipzig<br>Tel.: +49 (0)341 3910195<br>Fax: +49 (0)341 3910196

This trial will be conducted in compliance with this protocol, ICH GCP and applicable regulatory requirements.
The information contained in this document is proprietary to Vifor Pharma and is not to be copied, disclosed or otherwise distributed by an outside authority to any person or persons without the prior written permission of Vifor Pharma.

SIGNATURE PAGE

Declaration of Sponsor

Title: A Multicentre Randomised Placebo-controlled Study to Assess the Efficacy and Safety of a Single Administration of Ferric Carboxymaltose (1,000 mg iron) in Improving Fatigue Symptoms in Iron-deficient Non-anaemic (IDNA) Women of Child Bearing Age.

Version Number/Date: Version 1 dated 25 November 2009.

This study protocol was subjected to critical review. The information it contains is consistent with current knowledge of the risks and benefits of the investigational product, as well as with the moral, ethical and scientific principles governing clinical research as set out in the Declaration of Helsinki, 1996, and the International Conference on Harmonisation guidelines on Good Clinical Practice.

Lise Riopel, PhD<br>Clinical Representative<br>Vifor Pharma	09. 12. 2009<br>Date (day month year)
Thomas Keller, PhD<br>Trial Statistician<br>Consultant to Vifor Pharma	04.12.2009<br>Date (day month year)
Frits van Alphen, MD<br>Sponsor Medical Expert<br>Vifor Pharma	08-12-2009<br>Date (day month year)

INVESTIGATOR AGREEMENT AND SIGNATURE PAGE

I have read the attached protocol entitled "A Multicentre Randomised Placebo-controlled Study to Assess the Efficacy and Safety of a Single Administration of Ferric Carboxymaltose (1,000 mg iron) in Improving Fatigue Symptoms in Iron-deficient Non-anaemic (IDNA) Women of Child Bearing Age" dated 25 November 2009, and agree to abide by all provisions set forth therein.

I agree to comply with the current International Conference on Harmonisation Guideline on Good Clinical Practice and applicable regulations and guidelines.

I agree to ensure that financial disclosure statements will be completed by:

1. me (including, if applicable, my spouse (or legal partner) and dependent children)

2. my Sub-investigators

before the start of the study and to report any changes that affect my financial disclosure status for up to 1 year after the study is completed.

I agree to ensure that the confidential information contained in this document will not be used for any purpose other than the evaluation or conduct of the clinical investigation without the prior written consent of Vifor Pharma.

Signature by the Investigator on this Protocol Signature Page documents review, agreement and approval of the requirements contained within this protocol.

Signature	Date (day month year)

Name of Principal Investigator

SYNOPSIS

Protocol Number - IDNA 2009-01

Title	A Multicentre Randomised Placebo-controlled Study to Assess the Efficacy and Safety of a Single Administration of Ferric Carboxymaltose (1,000 mg iron) in Improving Fatigue Symptoms in Iron-deficient Non-anaemic (IDNA) Women of Child Bearing Age
Short Title	Effect of ferric carboxymaltose in improving fatigue in iron deficient non-anaemic women
Study Product(s)	Ferric carboxymaltose (FCM)
Phase	4
Sponsor	Vifor Pharma
Protocol Number	IDNA 2009-01
Study Code	PREFER
Principal Investigator	Dr. Bernard Favrat
Strategic Rationale	Claim support
Objectives	Primary Objective<br>• To assess the efficacy of a single intravenous (IV) administration of FCM (1,000 mg) compared with placebo in improving fatigue symptoms in IDNA women of child bearing age.<br>Secondary Objectives<br>• To compare efficacy of a single IV application of FCM with that of placebo on change of iron status on Day 56 (i.e., proportion of subjects with haemoglobin (Hb) ≥12 g/dL; serum-ferritin (s-ferritin) ≥50 ng/mL; transferrin saturation (TfS) >20%).<br>• To determine the relationship between change in iron status (s-ferritin and TfS) and improvement of fatigue symptoms.<br>• To compare the efficacy of a single IV administration of FCM with that of placebo in improving cognitive function (attention, concentration and short-term memory).<br>• To assess the safety of single IV administration of FCM.
Design	Multicentre, randomised, placebo-controlled, single-blinded, parallel group, comparative superiority study
Treatment	Study Drugs<br>1. Ferric carboxymaltose (commercial batch).<br>2. Placebo (0.9% sterile sodium chloride solution).
No. of Randomised Subjects	288
Number of sites	20
Inclusion Criteria	1. Signed informed consent prior to study specific procedures.<br>2. Premenopausal, regularly menstruating women.<br>3. Age ≥18 years.<br>4. Body weight between 50 and 90 kg.<br>5. Haemoglobin ≥115 g/L.

Inclusion Criteria (Cont'd)	6. Iron deficiency at screening defined as follows:<br>– S-ferritin level <50 ng/mL, AND, TfS <20%, OR,<br>– S-ferritin level <15 ng/mL.<br>7. Serum C-reactive protein:<br>– <5 mg/L if not on oral contraception, OR,<br>– <20 mg/L if use of oral contraception.<br>8. Minimum total score of 5 on the Piper Fatigue Scale (PFS) (mean of items 2 to 23).<br>9. Negative pregnancy test (serum human chorionic gonadotropin (hCG) at screening.<br>10. Normal levels of vitamin B_{12} and folic acid at screening.<br>11. Adequate contraception during the study period and for 1 month following study completion.<br>12. Availability and willingness to complete all study visits and procedures per protocol.
Exclusion Criteria	1. Haemoglobin level <115 g/L.<br>2. Haemoglobinopathy.<br>3. Haemochromatose.<br>4. Major depressive disorder based on Patient Health Questionnaire (PHQ-9) (5 items with scores ≥2; one of which corresponds to question number 1 or 2).<br>5. Any active or unstable concurrent medical condition (e.g., cancer, renal dysfunction, liver dysfunction (aspartate aminotransferase (AST); alanine aminotransferase (ALT) >3-fold upper limit), angina (Class IV).<br>6. Known human immunodeficiency virus/acquired immunodeficiency syndrome, hepatitis B virus or hepatitis C virus infection.<br>7. Chronic inflammatory disease (e.g., rheumatoid arthritis; inflammatory bowel disease).<br>8. Documented history of clinically significant level of sleep apnoea defined as 5 or more episodes per hour of any type of apnoea.<br>9. Intake of concurrent medications that could interfere with physical or mental performance (e.g., antidepressive, antihistamines, narcotic or any chemotherapeutic agents known to cause drowsiness).<br>10. Important recent weight loss (>10% within the past month).<br>11. Body weight <50 kg or >90 kg.<br>12. Thyroid dysfunction, thyroid stimulating hormone >4 µU/mL.<br>13. Intake of iron preparations 4 weeks prior to screening.<br>14. Use of gestagens e.g., Implanon® Mirena®, Depo-Provera® for menstruation repression (see Section 7.7, Prohibited Therapy or Concomitant Treatment, page 35).<br>15. Known hypersensitivity to FCM or to any other iron preparation.<br>16. Pregnancy (positive hCG test at screening) or breast feeding.

Exclusion Criteria (Cont'd)	17. Participation in any other interventional trial within 4 weeks prior to screening.<br>18. Inability to fully comprehend and/or perform study procedures or provide written consent in the Investigator's opinion.<br>19. Subject is not using adequate contraceptive precautions during the study and for up to 1 month after the last dose of the study medication. A highly effective method of birth control is defined as those which result in a low failure rate (i.e., less than 1% per year) when used consistently and correctly such as implants, injectables, combined oral contraceptives, some intra-uterine devices, sexual abstinence or vasectomised partner.<br>20. Subject previously has entered this study.<br>21. Subject will not be available for follow-up assessments.
Primary and Secondary Endpoints	Primary Efficacy Endpoint<br>• The proportion of responders defined as subjects who have a decrease in total score of PFS (mean of items 2 to 23) of at least 1 point from baseline on Study Day 56.<br>Secondary Endpoints<br>• Proportion of subjects with a decrease of at least 60 msec from baseline on cognitive function tests (total score) compared with placebo on Day 56.<br>• Mean change from baseline in cognitive functions tests total score compared with placebo.<br>• Mean change from baseline in cognitive function sub-tasks scores compared with placebo.<br>• Proportion of subjects who discontinue early due to lack of efficacy (no improvement, exacerbation of symptoms or worsening of fatigue).<br>• Mean change from baseline in PFS total score (mean of item 2 to 23) compared with placebo.<br>• Mean change from baseline in PFS of each of the 4 domains subscale scores compared with placebo on Day 56.<br>• Mean change from baseline in SF-12 total score compared with placebo on Day 56 (or early termination).<br>• Mean change from baseline in the mental health summary measures of SF-12 on Day 56 (or early termination).<br>• Mean change from baseline in physical health summary scores of SF-12 on Day 56 (or early termination).<br>• Mean change from baseline in restless leg syndrome (RLS) rating scale score compared with placebo if applicable.<br>• Proportion of subjects with Hb ≥12 g/dL; s-ferritin ≥50 ng/mL; TfS >20%.<br>• Relationships between change in s-ferritin level and improvement on PFS on Day 56.<br>• Relationships between change in s-ferritin level and success rate (decrease of at least 60 msec) of cognitive function test on Day 56.
Safety Endpoints	Frequency of adverse events.<br>Frequency of abnormal laboratory parameters.<br>• Haematology: (complete blood count, s-ferritin, TfS, soluble transferrin receptor, serum-iron).<br>• Blood biochemistry: ALT, AST, creatinine and phosphate.

Procedures	<u>Screening and Baseline Period</u><br>• Informed consent<br>• Pregnancy test (serum hCG)<br>• Blood biochemistry (ALT, AST, creatinine, phosphate)<br>• Urinalysis<br>• Haematology and iron status (serum-iron, s-ferritin, Tfs, soluble transferrin receptor)<br>• Vitamin B_{12} and folic acid<br>• Thyroid stimulating hormone<br>• Medical history, including concomitant medication<br>• Physical exam, including vital signs, body weight<br>• PHQ-9 depression screener (questionnaire)<br>• RLS questionnaire (diagnostic and rating if applicable)<br>• PFS (patient report outcome (PRO) questionnaire)<br>• SF-12 health survey (PRO questionnaire)<br>• Cognitive function tests (computer-based)<br>• Serious adverse events<br><u>Treatment Period (Interim Visits (Day 7 and Day 28))</u><br>• Haematology and iron status<br>• Blood biochemistry on Day 7 (+2 days)<br>• PFS<br>• RLS rating scale (if applicable)<br>• Cognitive function tests<br>• Vital signs (blood pressure, heart rate, body temperature)<br>• Concomitant medications<br>• Adverse events<br><u>End of Study (Day 56 +/- 4 Days or Early Discontinuation)</u><br>• Haematology and iron status<br>• Blood biochemistry (ALT, AST, creatinine and phosphate)<br>• Urinalysis (with phosphate)<br>• Vital signs, body weight<br>• Concomitant medications<br>• Adverse event<br>• PFS<br>• SF-12 health survey<br>• RLS rating scale (if applicable)<br>• Cognitive function tests

Sample Size	A two-group χ^2 test with a 0.050 two-sided significance level will have 80% power to detect the difference between placebo verum group proportion, $P_{Placebo}$, of 0.500 and verum group proportion, P_{Verum}, of 0.670 (odds ratio of 2.03) when the sample size in each group is 131. Assuming a drop-out rate of approximately 10%, a total of 288 subjects will be required for this study (144 per treatment arm). Drop-out patients will not be replaced.
Statistical Methods	<u>Randomisation</u><br>Block randomisation schemes will be applied, whereby blocks refer to centres.<br><u>Statistical Methods for Analysis</u><br>Descriptive statistics: contingency tables (categorically scaled variables by treatment: absolute and relative frequencies) and tables (mean, standard deviation, standard error, median, interquartile range, minimum, maximum; by treatment) for continuously scaled variables.<br><u>Primary Objective</u><br>Comparison of fractions P_{Verum} and $P_{Placebo}$ of subjects with improvement of total sum (PFS)≥1 via χ^2–Test (two-sided, alpha-level: 0.05). Null hypothesis: $P_{Verum}=P_{Placebo}$.<br><u>Secondary and Further Objectives</u><br>Differences between groups:<br>Statistical tests are performed, results are reported in a descriptive manner<br>fractions: χ^2-Test,<br>means/medians: t-Test or Mann Whitneys U-Test depending on distribution.<br>Relationships between parameters<br>Correlation analysis (Pearson, Rank-Spearman) depending on distribution.<br><u>Further Evaluations</u><br>Evaluation of centre effects, influence of baseline values, analysis of interactions: general linear (mixed) models.<br><u>Missing Values</u><br>Current available methods are applied to address missing values.<br><u>Interim Analysis</u><br>None planned.<br><u>Analysis Population</u><br>Primary: Intent-to-Treat<br>Secondary: Per-protocol<br><u>Software</u><br>Analysis: SAS 9.2

SCHEMA

Vifor Pharma
Vifor International

FERINJECT® – PREFER

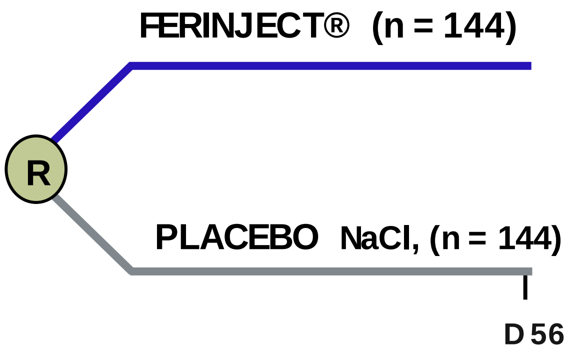

3 © Galenica Group 04.11.2009

SCHEDULE OF EVENTS

Visit	V1	V2	V3	V4	V5	V6
**Time**	**-14 to -1 Days**	**Day 0**	**Day 7 (±2 Days)**	**Day 28 (±4 Days)**	**Day 56 (±4 Days)/ Early Term.**	**Follow-up**
**Assessment**						
Informed consent	✓					
Medical history	✓					
Serum pregnancy test	✓				✓	
Concomitant medications	✓	✓	✓	✓	✓	
Physical examination	✓					
Vital signs	✓	✓	✓	✓	✓	
Haematology	✓		✓	✓	✓	
Iron status (s-ferritin, TfS, soluble transferrin receptor, serum-iron)	✓		✓	✓	✓	
Blood biochemistry	✓		✓		✓	
Thyroid stimulating hormone	✓					
Vitamin B_{12} and folic acid	✓					
PHQ-9	✓					
Restless leg syndrome diagnostic and rating scale[1]	✓	✓	✓	✓	✓	
Piper fatigue scale	✓	✓	✓	✓	✓	
SF-12		✓			✓	
Computerised cognitive tests		✓[2]		✓	✓	
Study drug administration		✓				
Adverse events[3]	✓	✓	✓	✓	✓	✓[4]

1 Restless leg syndrome diagnostic criteria will be applied at screening, RLS rating scale will be performed at every visit only if the RLS diagnostic is confirmed.
2 Training sessions will be performed before baseline assessments.
3 Adverse events will be recorded at each visit starting at Visit 2; All AE or serious AE occurring after consent will be recorded.
4 A follow-up call will be made 2 weeks after the last study visit to record the status of any ongoing AEs from Visit 5.
Notes: AE = Adverse event; RLS = Restless leg syndrome; s-ferritin = Serum ferritin; Term. = Termination; TfS = Transferrin saturation.

TABLE OF CONTENTS

Page

SIGNATURE PAGE 2

INVESTIGATOR AGREEMENT AND SIGNATURE PAGE 3

SYNOPSIS 4

SCHEMA 9

SCHEDULE OF EVENTS 10

TABLE OF CONTENTS 11

LIST OF APPENDICES 14

LIST OF ABBREVIATIONS 15

1. INTRODUCTION AND BACKGROUND 17

1.1 Background of the Disease and Treatment Options 17

1.2 Summary of Nonclinical and Clinical Data 20

2. RATIONALE 23

2.1 Rationale for the Study 23

2.2 Rationale for Dose Selection 23

3. STUDY OBJECTIVES 25

3.1 Primary Objective 25

3.2 Secondary Objectives 25

4. INVESTIGATIONAL PLAN 26

4.1 Overall Study Design 26

4.2 Discussion of Study Design 27

4.3 Duration of Subject Participation and Study 28

5. SELECTION AND WITHDRAWAL OF PATIENTS 29

5.1 Number of Patients 29

5.2 Inclusion Criteria 29

5.3 Exclusion Criteria 30

5.4 Withdrawal of Patients 31

6. RANDOMISATION, BLINDING AND UNBLINDING PROCEDURES 32

6.1 Randomisation/Treatment Allocation 32

6.2 Blinding 32

6.3 Unblinding 32

7. STUDY DRUGS: SUPPLY, PACKAGING, LABELLING AND STORAGE 33

7.1 Study Supply 33

7.1.1 Study Drug 33

7.1.2 Placebo 33

7.1.3 Devices....33
7.2 Packaging, Labelling and Storage....33
7.3 Storage....34
7.4 Drug Dosage and Administration....34
7.5 Drug Dose Modification....34
7.6 Accountability....35
7.7 Prohibited Therapy or Concomitant Treatment....35

8. RISKS/PRECAUTIONS....37

9. STUDY PROCEDURES....38
9.1 Description of Study Assessments....38
9.1.1 Efficacy Assessments....38
9.1.1.1 Patient Report Outcome and Screening Questionnaires....38
9.1.1.2 Cognitive Function Assessments (Appendix 5.5)....40
9.1.2 Safety Assessments....42
9.1.2.1 Laboratory Parameters....42
9.1.2.2 Vital Signs....42
9.1.2.3 Physical Examination....43
9.2 Schedule of Events....43
9.2.1 End of Treatment (or Early Discontinuation) Procedures....43
9.2.2 Follow-up Procedures....43

10. EVALUATION, RECORDING AND REPORTING OF AES AND SAES....44
10.1 Definitions....44
10.1.1 Adverse Event....44
10.1.2 Adverse Drug Reaction....44
10.1.3 Unexpected AE/ADR....44
10.1.4 Serious Adverse Event....44
10.1.5 Suspected Unexpected Serious Adverse Reaction....45
10.2 Adverse Event Descriptors....45
10.2.1 Intensity/Severity Categorisation....45
10.2.2 Causal Relationship Categorisation....46
10.2.3 Outcome Categorisation....46
10.2.4 Symptoms of the Disease Under Study....47
10.2.5 Clinical Laboratory Evaluations....47
10.2.6 Abuse, Misuse, Overdose and Medication Error....47
10.3 Reporting Procedure for AEs, SAEs and Pregnancy....47
10.3.1 Adverse Events....47
10.3.2 Serious Adverse Events....48
10.3.2.1 Elective Surgery/Routine Examination....50
10.3.3 Pregnancy....50

11. STUDY VARIABLES AND STATISTICAL ANALYSIS....52
11.1 Statistical Methods....52
11.1.1 Populations....52

11.2 Background and Demographic Characteristics....52
11.3 Study Medication....52
11.4 Medical History and Concomitant Diagnoses and Therapy....53
11.5 Efficacy Evaluations....53
11.5.1 Primary....53
11.5.2 Secondary....54
11.6 Safety Evaluations....55
11.7 Interim Analyses....56
11.8 Other Topics....56
11.8.1 Missing Values....56
11.8.2 Assessment of Interactions, Covariates, Centre Effects....57
11.8.3 Pharmacokinetics....57
11.8.4 Quality of Life....57
11.9 Sample Size and Power Calculations....57
11.10 Statistical Software....58

12. STUDY ETHICAL CONSIDERATIONS....59
12.1 Ethical Conduct of the Study....59
12.2 Informed Consent....59
12.3 Institutional Review Board or EC/IEC....59
12.4 Quality Control and Quality Assurance....59

13. ADMINISTRATIVE PROCEDURES....61
13.1 Sponsor's Responsibilities....61
13.1.1 Study Supplies....61
13.1.2 Insurance....61
13.1.3 Investigator Training....61
13.1.4 Study Monitoring....61
13.2 Investigator's Responsibilities....62
13.2.1 Reporting and Recording of Data....62
13.2.2 Source Documentation....62
13.2.3 Records Retention....63
13.2.4 Site Documentation....63
13.3 Other Services....63

14. PROCEDURE FOR MODIFICATION OF PROTOCOL OR PREMATURE TERMINATION OF THE STUDY....64
14.1 Protocol Waivers, Deviations and Violations....64
14.2 Protocol Amendments....64
14.3 Study Termination....64
14.4 Protocol Amendments....64
14.5 Study Termination....64

15. POLICY FOR PUBLICATION AND PRESENTATION OF DATA....65

16. REFERENCES....66

LIST OF APPENDICES

Page

Appendix 1 Summary of Product Characteristics (SmPC)....................................70
Appendix 2 Example Serious Adverse Event Reporting Form 79
Appendix 3 Example Pregnancy Reporting Form..81
Appendices 4 Informed Consent Documents 83
Appendix 4.1 Template Informed Consent Form...83
Appendix 4.2 Guidelines for Administering Informed Consent 91
Appendices 5 Patient Report Outcome Questionnaires...93
Appendix 5.1 Patient Health Questionnaire-9 93
Appendix 5.2 Piper Fatigue Scale and Scoring Instructions 95
Appendix 5.3 SF-12...100
Appendix 5.4a Restless Leg Syndrome Diagnostic Criteria 103
Appendix 5.4b Restless Leg Syndrome Rating Scale 104
Appendix 5.5 Cognitive Drug Research Cognitive Assessments............................105

LIST OF ABBREVIATIONS

ADR	Adverse drug reaction
AE	Adverse event
ALT	Alanine aminotransferase
AST	Aspartate aminotransferase
BFI	Brief symptom inventory
CDR	Cognitive Drug Research
CFR	Code of Federal Regulations
CI	Confidence interval
CKD	Chronic kidney disease
CRA	Clinical Research Associate
CRF	Case report form
CRO	Contract Research Organisation
EC	Ethics Committee
eCRF	Electronic case report form
EU	European Union
FCM	Ferric carboxymaltose
GCP	Good Clinical Practice
GLM	Generalised linear models
GLMM	Generalised linear mixed models
GMP	Good Manufacturing Practice
Hb	Haemoglobin
hCG	Human chorionic gonadotropin
IBD	Inflammatory bowel disease
ICF	Informed consent form
ICH	International Conference on Harmonisation
ID	Iron deficiency
IDA	Iron deficiency anaemia
IDNA	Iron deficiency non-anaemia
IEC	Independent Ethics Committee
IRB	Institutional Review Board
ITT	Intent-to-treat

IV	Intravenous
LOCF	Last observation carried forward
mcg	Micrograms
NDD-CKD	Non-dialysis-dependent chronic kidney disease
Opa bags	Opaque bags
PFS	Piper Fatigue Scale
PHQ-9	Patient Health Questionnaire-9
PP	Per-protocol
PRO	Patient report outcome
RBC	Red blood cell
RLS	Restless leg syndrome
SAE	Serious adverse event
SAP	Statistical analysis plan
SF-12	12-item short-form health survey
s-ferritin	Serum ferritin
SOC	System organ class
TfS	Transferrin saturation
US	United States
WHO	World Health Organisation
WMA	World Medical Association

1. INTRODUCTION AND BACKGROUND

1.1 Background of the Disease and Treatment Options

Iron is essential for virtually all types of cells and organisms. Not only is iron a functional component of the oxygen-carrying protein haemoglobin (Hb), it is also necessary for many major metabolic pathways. These include cellular respiration, glycolysis, fatty acid oxidation and deoxyribonucleic acid synthesis [1,2].

Iron deficiency (ID) is a state in which there is insufficient iron to maintain the normal physiological function of tissues such as the blood, brain, and muscles. If not treated ID leads inevitably to anaemia. However, ID can exist in the absence of anaemia if it has not lasted long enough or if it has not been severe enough to cause the Hb concentration to fall below the threshold for the specific sex and age group [3]. Evidence from animals fed on iron-deficient diet indicate that ID becomes detectable at about the same time in the blood, brain, and tissue enzyme system [4].

Serum ferritin (s-ferritin), a cellular iron-binding protein which is also secreted into the blood, provides the most reliable indirect measure of body iron stores if there is no concurrent infection. Serum ferritin levels below 15 ng/mL are highly specific for empty iron stores. Values from 15-30 ng/mL correspond to empty to limited iron stores; values from 30 to 50 ng/mL are borderline and values above 50 ng/mL without coexisting inflammation/infection are evidence of sufficient iron reserves [5]. Serum iron reflects the balance of iron flow in and out of the plasma pool. Transferrin is a serum glycoprotein that carries iron to the cells via transferrin receptors. A serum iron concentration of 1 mg/L corresponds to a transferrin saturation (TfS) of 30%. Lower TfS is frequently used as an indicator of ID.

The World Health Organisation (WHO) has identified ID to be one of the most prevalent nutrient deficiencies in the world which may affect human health [6]. This disorder is not limited to developing countries. Although modern diet and the use of iron supplements have reduced the incidence and degree of ID in industrialised countries, iron supply is still a problem in certain subgroups of the population, namely young children and menstruating women. A French epidemiological survey showed that about 20% of menstruating women had ID indicated by ferritin concentration less than 15 ng/mL but only 4% of these women had iron deficiency anaemia (IDA) [7]. In Europe the prevalence of ID without anaemia varied from 4 to 33% depending on region studied [8]. A more recent study conducted in Finland showed that s-ferritin levels in women less than 50 years of age average 32 ng/mL and about 20% of these women had s-ferritin levels below 12-15 ng/mL [9].

The non-haematological effects of ID have been known for decades. Specific skin and mucosal symptoms usually appear only in established anaemia. However, impaired physical condition, cognitive impairment, disturbed thermoregulation and fatigue have also been described in women with ID in the absence of anaemia. They improve with iron therapy [10-17].

Iron deficiency in the absence of anaemia has been associated to a reduced cognitive function [18]. Animal models have revealed several mechanisms by which ID may affect cognition: Decreased brain iron store impairs the activity of iron-dependent enzymes necessary for the synthesis, function and degradation of neurotransmitters, such as dopamine, serotonin, and noradrenaline [19,20,21,22]. Interestingly, iron content in the central nervous system decreases before red blood cell (RBC) production is affected by ID. Based on these findings it is reasonable to assume that ID in the absence of anaemia will affect the normal functioning of the brain, and therefore will result in the occurrence of unspecific symptoms such as sleep and mood disorders, impaired cognitive function or fatigue. Indeed, several clinical studies have shown that ID causes changes in behaviour and decreases development test scores in young children [23,24,25]. Studies in adolescents and young women have shown that iron supplementation improved verbal learning, memory and overall cognitive functioning [14,18].

Motor activity and exploratory behaviour are known to be dopamine dependent [22,26]. Iron content and the dopamine D2-receptor density in the brain are correlated [21]. The association between restless leg syndrome (RLS) and ID has been shown in several clinical studies [27]. Sun et al, 1998 could show for example that almost all patients with severe RLS had s-ferritin levels below 50 ng/mL and low levels were significantly correlated with a decreased sleep efficiency. Oral iron supplementation in patients with low s-ferritin values clearly reduced the RLS symptoms [28] and high-dose intravenous (IV) iron brought complete relief of the symptoms [29]. The efficacy of IV iron sucrose (1,000 mg iron) was also evaluated in a double-blind placebo-controlled clinical study in patients with RLS and mild to moderate iron deficit; iron treatment reduced RLS symptoms in the acute phase and long-term follow-up but failed to show superiority over placebo with respect to total score of RLS rating scale [30].

Physical performance can be impaired due to ID without concomitant anaemia. In 2 randomised placebo-controlled studies, Brownlie et al [13,31] showed that oral iron supplementation 20 mg daily for 6 weeks improved aerobic adaptation and endurance capacity in untrained women. Iron supplementation in iron deficient non-anaemic (IDNA) women was also associated with a significant improvement in muscle fatiguability [17,32].

Verdon et al, 2003 showed that oral iron therapy in non-anaemic women was superior to placebo in improving fatigue assessed on visual analogue scale [12]. Mansson et al, 2005 showed reduced symptoms of dizziness, irritability, depressive symptoms and indisposition following a 3-month oral iron treatment [10]. These results are in line with those of an Australian study which showed also a decrease in mean total score of the Piper Fatigue Scale (PFS) after iron supplementation compared with iron rich diet alone [16]. Ballin et al, 1992 [33] showed that iron supplementation results in improvement in lassitude, ability to concentrate and mood in non-anaemic ID adolescents. A recent placebo-controlled study conducted in women with s-ferritin below 50 ng/mL and complaining of unspecific symptoms (exhaustion, depressed

mood, lack of concentration) suggest that treatment with IV iron sucrose (Venofer®: 4 x 200 mg over 2 weeks) may be useful to relieve fatigue. In this study fatigue was assessed using the brief symptom inventory (BFI), as primary endpoint. There was a trend for greater improvement of the BFI total score, from baseline to Day 42, in patients treated with IV iron sucrose compared to patients treated with placebo (p=0.076). Further, there was significantly greater improvement in fatigue from baseline to Day 42 in patients treated with IV iron sucrose compared to patients treated with placebo as measured by categorised changes in BFI scores (p=0.036). In patients presenting with depleted iron stores at baseline, defined as TfS below 20% and s-ferritin below 50 ng/mL or s-ferritin below 15 ng/mL, there was a significantly greater improvement in fatigue at Day 42, as measured by the BFI, in patients treated with IV iron sucrose compared to patients treated with placebo (p=0.026) [34].

However, these results contrast with those of other studies which have failed to show a direct correlation between iron status and non-specific symptoms [35].

The apparent inconsistent findings of iron therapy on improvement of non-specific symptoms such as fatigue may result in part from the design employed (generally underpowered), the heterogeneity of the patient populations (disease characteristics, ferritin threshold levels defining ID) and the diversity of outcome measures (visual analogue scale, multiple fatigue assessment instruments, Quality of Life instrument, etc.). The various iron form, dose, route (oral or parenteral) and treatment duration may also explain the inconclusive outcome of these studies.

The iron deficit in non-anaemic women lies between 500 mg and 1,000 mg, although some studies suggest that 500 mg may not be sufficient to replenish the iron stores. Oral supplementation with iron sulphate is usually the first line treatment when no other pathology is present. The usual dose for adults is 150-200 mg iron per day for several weeks. Absorption rate rarely exceeds 10% which is insufficient to raise the s-ferritin values significantly. Most patients tolerate oral iron therapy but 10-40% may experience adverse reactions attributable to iron such as nausea or epigastric discomfort. These adverse effects occur within 1 hour after ingestion and may be mild or be more serious with pain, vomiting, diarrhoea or constipation. The incidence of these adverse reactions increases with the dose, which often requires lowering the dose or changing the iron formulation. Other iron formulation such as iron fumarate permit slower release of iron salts, which causes fewer adverse effects [36].

Despite the improvement in oral iron formulations, several limitations are associated to oral therapy: poor absorption results in a slow onset of clinical effect and the intolerance or poor compliance result in poor efficacy. Parenteral iron is then indicated in patients with poor iron absorption, intolerance or when a rapid effect is required or desired. In recent years, we have gained a better understanding of the use of parenteral iron in the management of anaemia in a broad scope of clinical conditions. Additionally, more information is forthcoming on the efficacy and safety of a new parenteral iron product, ferric carboxymaltose (FCM), which represents a

significant advance over currently available products with respect to dose regimen, stability and safety.

Fatigue (exhaustion) is recognised as a multidimensional construct [37] and is one of the most common complaints in primary care medicine [38,39,40,41]. In many instances ID may be overlooked as possible cause and appropriate treatment is delayed. In recent years, many clinicians have gained valuable experience in recognising and treating symptomatic ID also in the absence of anaemia. However, data from well-designed and well-controlled studies are lacking in order to provide the convincing scientific evidence needed to introduce new guidelines for this condition. This randomised placebo-controlled study is aimed at demonstrating the clinical benefit of IV iron therapy in relieving fatigue symptoms in patients with confirmed ID using a convenient single administration of FCM.

Given the subjective nature of fatigue, self-report (patient report outcome (PRO)) is considered the best way to assess symptoms and treatment effectiveness. Several PRO instruments have been developed to document the severity of fatigue or general well-being but few have been used in this specific patient population or have been shown to be sensitive enough to detect treatment effect in drug trial. The current version of the PFS has undergone extensive testing in female populations and has been shown to have good reliability and sensitivity. Concurrent validity estimates have been determined by correlating the subscale with the mood disturbances scores of the profile Mood States and with the Fatigue Symptom Checklist subscales and Total Fatigue Scores [42]. Thus, the PFS is considered appropriate to assess fatigue in this study.

One well-established dimension of fatigue is the cognitive impairment. Therefore, fatigue will also be assessed using objective measures that focus on physiological process and performance such as reaction time, number of errors, etc. A validated computer based cognitive assessment tests will allow to identify the cognitive effect of iron therapy in these patients. Using the cognitive drug research (CDR) system attentional, working memory and episodic secondary memory tasks will be performed at baseline, interim and final study visits. Cognitive drug research system has been subject to extensive validation, demonstrating high degree of bi-directional sensitivity (impairment or enhancement).

1.2 Summary of Nonclinical and Clinical Data

Ferric carboxymaltose (5% weight to volume ratio iron containing FCM in a solution of water for injection) is a formulation of parenteral iron, developed by Vifor Pharma - Vifor (International) Inc. and approved for treatment of ID and IDA in the EU countries and Switzerland.

Overall, nonclinical studies performed with FCM have demonstrated that this is an effective complex for delivery of iron to target tissues in the treatment of ID. The toxicities observed in the various studies performed were all considered to have

resulted from iron overload in healthy iron-replete animals and, providing the iron dose is carefully tailored to the needs of each patient, toxicity is very unlikely during clinical use of the product. Data from the reproductive and developmental toxicity studies do not reveal any data other than that which might be expected from iron-overloaded animals. The compound does not show any cross reactivity with anti-dextran antibodies. The carbohydrate component of FCM is considered to have low immunogenic potential due to its composition, and the ready breakdown to simple oligo-glucose units. The efficacy and safety profile of FCM as assessed from these nonclinical studies is considered similar to or, in some cases, more favourable to that of other currently approved parenteral iron complexes such as iron dextran or iron sucrose (Venofer). The available nonclinical data support the use of FCM as a parenteral replacement therapy for the treatment of IDA.

Based on human pharmacokinetic data, the terminal half-life of FCM is 7-12 hours. The analysis of an FCM positron emission tomography study in 6 patients, each receiving a single dose of 100 mg iron as FCM, demonstrated that, during the initial distribution phase, a major proportion of the dose was distributed to the bone marrow [43].

Red cell utilisation of iron was found to be high. After 16 to 24 days, patients with IDA showed a red cell utilisation of 61-99%. A single-dose Phase 1 study demonstrated that FCM could be safely administered at doses of up to 1,000 mg iron. A multiple-dose Phase 1/2 study in patients with gastrointestinal disorders has been performed to prove the safety, efficacy, and kinetics of repeated doses of 500 and 1,000 mg iron as FCM. Another Phase 2 study has been performed to study the safety and efficacy of ascending single doses of FCM (100-1,000 mg) in patients with renal anaemia receiving dialysis treatment. Further details on FCM and these studies are available in the Investigator's Brochure.

The efficacy of FCM was compared to iron sucrose (Venofer) in a Phase 3 study (VIT-IV-CL-015) conducted in haemodialysis patients. The results show a more pronounced increase in Hb, s-ferritin and TfS in the FCM group compared to the Venofer group and in the patients with low Hb at baseline. As the differences occurred in several analyses and subgroups, they can be considered as a strong trend. There were no statistically significant differences between the FCM and the Venofer group.

Efficacy and safety of FCM were investigated in a prospective, randomised, controlled study conducted in inflammatory bowel disease (IBD) patients. According to the results of this study, FCM is safe and effective in the treatment of IDA; it provides a faster Hb response, a higher increase in iron storage and a better patient tolerance compared to oral preparations [44]. Studies in patients with postpartum anaemia showed, that FCM is effective and safe in the treatment of this condition [45].

Two studies have been conducted in non-dialysis-dependent chronic kidney disease (NDD-CKD), the 1VIT04004 and 1VIT05005 studies. Study 1VIT04004 was a

multicentre, randomised, open-label, active control, 2-arm, parallel-group Phase 3 study in the treatment of anaemia in patients with NDD-CKD. In the modified full analysis set population, the proportion of patients who achieved an increase from baseline in Hb by ≥10 g/L during the study was statistically significantly larger (Fisher's exact test, p<0.001) in the FCM group (60.4%) than in the ferrous sulphate group (34.7%). Study 1VIT05005 was an open-label, multicentre, non-randomised, longitudinal single-arm, follow-up to Study 1VIT04004, to evaluate the long-term safety, tolerability and efficacy of an IV FCM maintenance dosing strategy in the treatment of IDA in patients with NDD-CKD. In the efficacy set, 72 patients (51.4%) achieved clinical success (Hb ≥110 g/L, ferritin between ≥100 and ≤800 ng/mL, and TfS between ≥30% and ≤50%) at any time during the study and 14 patients (10.0%) achieved sustained clinical success (clinical success at ≥50% of the assessments). Sustained clinical success was most pronounced in patients with a baseline Hb between 101 and 110 g/L and in patients with baseline ferritin levels <100 ng/mL. An Hb level ≥110 g/L at any time in the study was achieved by 87.9% patients (by-time-point range: 55.0% on Day 84 to 64.3% on Day 308). A ferritin level between ≥100 and ≤800 micrograms (mcg)/L at any time during the study was achieved by 99.3% of patients. A TfS level between ≥30 and ≤50% at any time in the study was achieved by 75.7% of patients. Highest mean changes from baseline during the study were: 18.9 g/L for Hb, 20.37% for TfS, 744.56 mcg/L for ferritin, 0.71% for reticulocyte count, and 2.35 pg for reticulocyte Hb content.

2. RATIONALE

2.1 Rationale for the Study

Fatigue is major public health problem with a prevalence reported to be in the range of 13-33% in primary care setting [38,39,40,41]. Accumulating clinical reports and observations suggest that iron replacement therapy is effective in relieving fatigue in patients with ID even in the absence of anaemia. Findings from a previous study warrant further investigations in order to confirm the clinical benefit of IV iron therapy in symptomatic IDNA women [34] and to better define ID threshold requiring iron replacement.

Additionally, data on the relationship between improvement of fatigue symptoms and normalisation of iron status is still lacking. If a relationship is established this study will provide strong evidence for the need of adequate medical management of unspecific fatigue symptoms associated to depleted iron stores in the absence of anaemia.

This study shall also confirm the safety and tolerance of a single dose of 1,000 mg of FCM in this patient population.

Potential Risks and Benefits

As with all iron preparations, overdosing with respect to the total amount must be avoided. Based on animal toxicity data and clinical experience to date, FCM does not cause anaphylactic reactions or liver toxicity at the doses intended for use in this study. However, due to the relatively large doses of iron being administered, patients will be monitored carefully throughout the study for symptoms of iron overload.

Potential benefits to the patients include restoration of iron stores and as a consequence improvement of fatigue symptoms.

2.2 Rationale for Dose Selection

A single dose of 1,000 mg iron (FCM) or placebo will be administered to eligible women with a body weight of at least 50 kg. The dose was selected on current clinical practice and published reports.

In a retrospective study IBD patients with anaemia were treated with Venofer at a dose calculated according to the Ganzoni formula, which establish the iron store requirement at 500 mg. At the end of the treatment many patients were still ID [46]. In a recent study [47], iron sucrose (Venofer) was administered at a dose calculated according to Ganzoni, but 1,000 mg instead of 500 was used as iron store threshold. Serum ferritin reached a mean of 130 ng/mL following treatment. Grote et al, 2009 [30] administered 5 times 200 mg of iron sucrose (Venofer) over 3 weeks to IDNA RLS patients: the treatment was well tolerated and there were no serious adverse events (SAEs) reported at this dose.

The Anaemia Working Group of Sweden in IBD has issued recommendations regarding IV iron treatment of ID and IDA. Patients with ID (s-ferritin <30 ng/mL) but without anaemia defined as Hb above 120 g/L in females or 130 g/L in males, a dose of 1,000 mg is recommended.

A dose of 1,000 mg in a patient with ID will on average increase s-ferritin by 100 ng/mL. This increase will result in an s-ferritin still within the normal range when the patients have depleted or empty iron stores at baseline [36].

In patients with chronic kidney disease (CKD), it is recommended to maintain s-ferritin >100 ng/mL, but in order to reach this level, ferritin levels should reach following iron therapy 200-500 ng/mL with an upper limit of 800 ng/mL.

Further the selected dose of 1,000 mg was shown to be safe and effective in various patient populations; the proposed regimen is easier to administer as there is no calculation required and the single administration is more comfortable to the patients. In clinical practice, the physician will favour a simple and standard dose regimen as there is less risk of dosing error.

3. STUDY OBJECTIVES

The objectives of this study are to demonstrate the efficacy and safety of a single IV administration of FCM in IDNA pre-menopausal women suffering from unexplained fatigue symptoms.

3.1 Primary Objective

- To assess the efficacy of a single intravenous (IV) administration of FCM (1,000 mg iron) compared with placebo in improving fatigue symptoms in IDNA women of child bearing age.

3.2 Secondary Objectives

- To compare efficacy of a single IV application of FCM with that of placebo on change of iron status on Day 56 (i.e., proportion of subjects with haemoglobin (Hb) ≥12 g/dL; serum-ferritin (s-ferritin) ≥50 ng/mL; transferrin saturation (TfS) >20%).

- To determine the relationship between change in iron status (s-ferritin and TfS) and improvement of fatigue symptoms.

- To compare the efficacy of a single IV administration of FCM with that of placebo in improving cognitive function (attention, concentration and short-term memory).

- To assess the safety of single IV administration of FCM.

4. INVESTIGATIONAL PLAN

4.1 Overall Study Design

This is a multicentre, randomised, single-blinded, placebo-controlled, comparative, parallel group study to assess the efficacy and safety of a single administration of FCM 1,000 mg iron in improving fatigue symptoms in iron-deficient non-anaemic (IDNA) women of child bearing age.

After obtaining consent patient will be screened for iron status and minimum eligible total score on the PFS. Other screening procedures are intended to preclude enrolment of patients with a pathology or medical condition that could explain fatigue symptoms. Special attention will be given to the Patient Health Questionnaire-9 (PHQ-9), a questionnaire aimed at ruling out a major depressive disorder. Questionnaires such as RLS diagnostic and rating scale or SF-12 and the cognitive function tests will be applied at baseline, prior to study drug administration but will not be used as selection criteria. Screening procedures should be completed within a maximum of 14 days of randomisation.

After completion of baseline assessments on Day 0, patient will be assigned a unique randomisation number corresponding to a treatment group. Each randomisation number will correspond to a study drug package labelled with the randomisation number. Investigator will assign randomisation number in ascending sequential order.

Patient will be randomised to receive either:

- Group A: Ferric carboxymaltose 1,000 mg iron, or
- Group B: Placebo (0.9% normal saline solution of IV injection)

To keep the patients blinded, the infusion pouch will be prepared in a separate room, since the colour of FCM is different from the placebo solution. A placebo solution matching the colour of FCM is not feasible. Treatment identification will be masked by covering the infusion kit with opaque bags (Opa bags) and infusion will be done via a dark coloured infusion set.

The Investigator will prepare and administer the study drug in accordance to the instructions provided in this protocol. Ferric carboxymaltose will be provided in 2 vials of 10 mL containing each 500 mg iron, which will be diluted in 250 mL normal saline for injection. Study drug will be administered by drip infusion immediately after preparation over a minimum of 15 minutes. Placebo patients will be administered 250 mL normal saline for injection over a minimum of 15 minutes.

The Investigator will monitor the vital signs until 1 hour after the infusion at which time patient may be released if the patient does not experience adverse reaction. Patient will be instructed to return to the study sites for interim visits on Day 7, Day 28 and End of Study on Day 56 (post baseline).

At interim visits, Investigator will assess safety parameters, such as vital signs, adverse events (AEs) and concomitant medications. Blood draws will be done on Day 7 and End of Study visit for clinical laboratory studies (haematology including iron status and biochemistry). Patient will be administered the computerised cognitive function tests. Investigator must ensure that patient did not ingest caffeine containing drinks or use nicotine containing product for 1 hour before the test. Patient should refrain from consuming alcoholic drink with 12 hours prior to testing.

Patient shall complete the PFS and other health questionnaires at the study site. These are self-administered questionnaires. The Investigator will review the questionnaires after completion to ensure patient answered all the questions prior to leaving the study site. Complete questionnaires will be stored in the patient case report form (CRF).

End of Study assessments must be performed at study completion on Day 56 or at the time of withdrawal for patients who terminate early for any reason.

Approximately 288 patients, 144 per treatment group, will be randomised across approximately 20 study sites in Switzerland, Sweden, Austria and Germany. The patient recruitment shall start during the first trimester of 2010 and be concluded during the second trimester of 2011.

4.2 Discussion of Study Design

A randomised design was chosen to reduce bias with regard to patient selection and baseline differences of the treatment groups. A placebo control group assessed under single-blind (patient is blinded) conditions will ensure a reliable evaluation of the study endpoints. Self-perceived fatigue has a high subjective component and therefore a placebo effect is expected. For this reason and because the study is aimed at establishing the scientific evidence for the need of iron replacement therapy in iron-deficient non-anaemic symptomatic patients, a placebo group is justified. The single blind condition would preclude that a patient (subjective) factor is introduced in the study evaluation.

Considerations Regarding Patient Population

The threshold for defining ID is based on generally accepted levels corresponding to empty iron stores and on the result of a previous study using iron sucrose [34]. The lower limit of Hb to define non-anaemic was set at 115 g/L to take into account the laboratory variations as well as physiological fluctuations of Hb levels in this population.

Considerations Regarding Primary Efficacy Endpoint

There are no direct observable or physical measures for assessing fatigue. Patient provides a unique perspective on treatment effectiveness because improvement in clinical measures of a condition may not necessarily correspond to improvement in how the patient functions or feels. Self-administered questionnaires (PRO) capture directly the patient’s perceived response to treatment without third-party

interpretation. The PFS is a self-administered research instrument which was used to measure subjective fatigue patterns in a variety of populations including young women with ID. The PFS was selected based on its sensitivity to detect treatment effect and because the scale was shown to be relevant to this patient population [16].

The effect related to primary efficacy endpoint will be measured by difference in fractions of patients who responded, whereby a patient responds when at least a decrease in total score of PFS of 1 unit is found.

Hereby, 2 decisions were made in terms of study design: choice of a dichotomous outcome (response/no response) and definition of response as 1 unit of PFS.

The decision for a dichotomous outcome to assess primary endpoint was made because it directly reflects patients benefit from therapy based on a predefined response. In support of the primary assessment treatment effect will also be assessed on a continuous scale (means of difference total scores of PFS versus baseline). However, this approach is not directly linked to individual patient's benefit from therapy.

Response defined as a decrease of 1 unit in total score of PFS relates to the fact that such patient reported outcome measure is constructed in a way that 1 unit describes a considerable difference in patient state.

4.3 Duration of Subject Participation and Study

The maximum duration of subject participation will be 70 ±4 days (maximum 14 days for screening and 56 days post treatment). To improve visit compliance, a window of 2 to 4 days will be allowed for interim and End of Study visit. If an AE is ongoing at the time of study completion the Investigator should ensure an appropriate follow up including, if necessary, additional clinic visit until the AE is resolved or that no further relevant medical information can be reasonably expected.

5. SELECTION AND WITHDRAWAL OF PATIENTS

5.1 Number of Patients

A total of 288 patients, 144 per treatment group, will be randomised across approximately 20 study sites in Switzerland, Sweden, Austria and Germany. Each site will be required to enrol 12 to 15 patients over approximately 15 months. Patients who withdraw early for any reasons will not be replaced. A complete sample size justification is provided in Section 11, Study Variables and Statistical Analysis, page 52.

5.2 Inclusion Criteria

1. Signed informed consent prior to study specific procedures.
2. Premenopausal, regularly menstruating women.
3. Age ≥18 years.
4. Body weight between 50 and 90 kg.
5. Haemoglobin ≥115 g/L.
6. Iron deficiency at screening defined as follows:
   - S-ferritin level <50 ng/mL, AND, TfS <20%, OR,
   - S-ferritin level <15 ng/mL.
7. Serum C-reactive protein:
   - <5 mg/L if not on oral contraception, OR,
   - <20 mg/L if use of oral contraception.
8. Minimum total score of 5 on the Piper Fatigue Scale (PFS) (mean of items 2 to 23).
9. Negative pregnancy test (serum human chorionic gonadotropin (hCG) at screening.
10. Normal levels of vitamin B_{12} and folic acid at screening.
11. Adequate contraception during the study period and for 1 month following study completion.
12. Availability and willingness to complete all study visits and procedures per protocol.

5.3 Exclusion Criteria

1. Haemoglobin level <115 g/L.
2. Haemoglobinopathy.
3. Haemochromatose.
4. Major depressive disorder based on Patient Health Questionnaire (PHQ-9) (5 items with scores ≥2; one of which corresponds to question number 1 or 2).
5. Any active or unstable concurrent medical condition (e.g., cancer, renal dysfunction, liver dysfunction (aspartate aminotransferase (AST); alanine aminotransferase (ALT) >3-fold upper limit), angina (Class IV).
6. Known human immunodeficiency virus/acquired immunodeficiency syndrome, hepatitis B virus or hepatitis C virus infection.
7. Chronic inflammatory disease (e.g., rheumatoid arthritis; inflammatory bowel disease).
8. Documented history of clinically significant level of sleep apnoea defined as 5 or more episodes per hour of any type of apnoea.
9. Intake of concurrent medications that could interfere with physical or mental performance (e.g., antidepressive, antihistamines, narcotic or any chemotherapeutic agents known to cause drowsiness).
10. Important recent weight loss (>10% within the past month).
11. Body weight <50 kg or >90 kg.
12. Thyroid dysfunction, thyroid stimulating hormone >4 μU/mL.
13. Intake of iron preparations 4 weeks prior to screening.
14. Use of gestagens e.g., Implanon® Mirena®, Depo-Provera® for menstruation repression (see Section 7.7, Prohibited Therapy or Concomitant Treatment, page 35).
15. Known hypersensitivity to FCM or to any other iron preparation.
16. Pregnancy (positive hCG test at screening) or breast feeding.
17. Participation in any other interventional trial within 4 weeks prior to screening.
18. Inability to fully comprehend and/or perform study procedures or provide written consent in the Investigator's opinion.
19. Subject is not using adequate contraceptive precautions during the study and for

up to 1 month after the last dose of the study medication. A highly effective method of birth control is defined as those which result in a low failure rate (i.e., less than 1% per year) when used consistently and correctly such as implants, injectables, combined oral contraceptives, some intra-uterine devices, sexual abstinence or vasectomised partner.

20. Subject previously has entered this study.

21. Subject will not be available for follow-up assessments.

5.4 Withdrawal of Patients

Patients may voluntarily withdraw for any reason. Patients may be withdrawn because of the appearance of a new health condition suspected to require appropriate care or require medications prohibited by the protocol, unacceptable AEs, refusal to continue treatment, or at the Investigator's discretion if it is in the patient's best interest according to the Investigator's clinical judgment.

Discontinuation from the study is mandatory in the following circumstances:

- Development of an intolerable AE, whether or not it is related to study participation.
- The patient withdraws consent, i.e., does not wish to continue, irrespective of the reason.
- Development of an illness, condition, or procedural complication, which would interfere with the patient's continued participation.
- The Investigator/study physician feels it is medically in the best interest of the patient to discontinue the patient's participation in the study.
- Intake of iron preparations other than the study drug.
- Pregnancy (to be immediately notified to the Sponsor).

If a patient withdraws from the study at any time either at her request or at the Investigator's discretion, the reason(s) for withdrawal must be recorded on the relevant page of the patient's CRF. Patients who withdraw from the study prematurely should undergo all end-of-study assessments.

It is vital to obtain follow-up data on any patient withdrawn because of an AE. In any case, every effort must be made to undertake protocol-specified safety follow-up procedures. If a patient is discontinued due to an AE, the event will be followed until resolution or until no further relevant medical information can be expected. If a patient refuses to continue study procedures, the reason for refusal should be fully documented in the patient's source document and recorded in the study specific CRF.

Patients who withdraw early for any reason will not be replaced.

6. RANDOMISATION, BLINDING AND UNBLINDING PROCEDURES

6.1 Randomisation/Treatment Allocation

All patients enrolled must be identifiable throughout the study. The Investigator will maintain a personal list of patient names, contact information, screening and randomisation number to enable records to be found at a later date.

Treatment allocation will be performed only after baseline assessments have been performed and confirmation of patient eligibility in the study. Randomisation to 1 of the 2 treatment arms will be performed based on a pre-defined, computer-generated, block randomisation list.

Patients eligible for randomisation will be assigned a randomisation number. Investigator will assign the randomisation number in a sequential ascending order. Investigator will select the study medication kit corresponding to the randomisation number.

6.2 Blinding

To keep the patients blinded, the infusion set will be prepared in a separate room, since the colour of FCM is different from the placebo solution. The infusion bags will be covered with opaque bags (Opa bags), and the infusion will be given via a dark coloured infusion tubing. At the time of drug administration patient will be asked to look on the opposite side for the duration of the infusion time.

6.3 Unblinding

Investigator will be provided a set of sealed envelopes, each corresponding to a randomisation number and containing the identity of the study drug. In case of emergency only, the envelope corresponding to the patient randomisation number may be opened and the blind be broken for that patient. Investigator should keep the envelopes in a restricted area. If the Investigator or an authorised person needs to break the blind, the reason(s) shall be explained in the patient CRF. All study medication envelopes shall be returned to the Sponsor with the study material at the end of the study.

7. STUDY DRUGS: SUPPLY, PACKAGING, LABELLING AND STORAGE

7.1 Study Supply

The study medication will be provided by Vifor Pharma - Vifor (International) Inc.

7.1.1 Study Drug

Drug name:	Ferinject®.
Active ingredient:	Ferric carboxymaltose.
Dosage form:	5% w/v iron containing 50 mg iron per mL, as sterile solution of FCM in water for injection.
Administration:	Intravenous route only. In this study FCM will be administered by drip infusion. Ferric carboxymaltose must be diluted only in 250 mL sterile 0.9% sodium chloride prior to administration. The minimum infusion time shall be 15 minutes.
Excipients:	Water.
Strength/Packaging:	2 x 10 mL vials each containing 500 mg iron as iron per vial.
Manufacturer:	Vifor Pharma - Vifor (International) Inc, Switzerland.
Storage:	Do not store above 30°C. Do not refrigerate or freeze.

7.1.2 Placebo

Sterile 0.9% sodium chloride for IV infusions will be used as placebo.

7.1.3 Devices

Opa bags and dark infusion tubing kit will be provided by Vifor Pharma within each randomisation kit.

7.2 Packaging, Labelling and Storage

All study supply will be provided by Vifor Pharma. All packaging and labelling operations will be performed according to Good Manufacturing Practice (GMP) and Good Clinical Practice (GCP) guidelines. The study drug will be labelled in accordance with local study site regulations for investigational products.

Ferric carboxymaltose will be packaged in a kit each containing 2 x 10 mL vial containing each 500 mg iron, 1 x 250 mL sterile saline for dilution, 1 Opa bag and dark coloured infusion line. The kit box will be labelled as well as each vial within the kit box.

Placebo kit will contain 1 x 250 mL sterile saline, 1 Opa bag and dark coloured infusion line.

All kit boxes, vials and bags will be labelled with the "randomisation number".

7.3 Storage

All study medication kits must be stored in a locked area at a temperature between 4 and 25°C.

Do not refrigerate or freeze.

Maintenance of a temperature log is mandatory. The log should be updated by site personnel once weekly.

7.4 Drug Dosage and Administration

Study Drug

Ferric carboxymaltose will be administered as a single dose via IV drip infusion by designated staff at each site. Details of infusion (volume and time) will be documented in the CRF.

The designated study staff will prepare the study medication in a restricted room which is not visible to the study patient.

The content of the 2 x 10 mL vial containing each 500 mg iron must be diluted in no more than 250 mL sterile 0.9% sodium chloride solution.

Infusion pouches will be placed into the Opa bag and will be used with the dark tubing device provided in the medication kit.

Patient will be administered 20 mL FCM diluted in a maximum of 250 mL of sterile 0.9% sodium chloride solution.

The infusion time must be at least 15 minutes.

Placebo

Patient randomised to placebo group will be administered a single dose of 250 mL of 0.9% sodium chloride solution via IV drip infusion. Placebo infusion pouches will be prepared and handled as described above.

The infusion time must be at least 15 minutes.

7.5 Drug Dose Modification

The dose to be administered should not be modified unless the patient experiences an adverse reaction in which case study drug infusion must be stopped immediately. The volume infused at the moment should be estimated and recorded in the CRF. The AE

will be reported as described in Section 10, Evaluation, Recording and Reporting of AEs and SAEs, page 44.

7.6 Accountability

The Investigator at each site is responsible for study drug supplies. The Investigator will ensure that adequate records of the receipt, preparation, administration and return of the study drug are kept and that the study drug is used only for subjects enrolled in the study. All data regarding the study drug must be recorded on the relevant forms provided.

Each study site will maintain a drug inventory/dispensing record for all drugs dispensed and returned. At the end of the study, 1 copy of the drug inventory/dispensing record should be sent to the Sponsor for the central study file. The original will be kept in the site files.

After completion of the study, or if it is prematurely terminated, all unused materials will be returned to the Sponsor. If the study medication is destroyed at site, the Investigator will forward the certificate of destruction to the Sponsor. The decision to destroy study medication at site must be made by the Sponsor.

7.7 Prohibited Therapy or Concomitant Treatment

Any concomitant treatment given for any reason must be recorded on the CRF, including dosage, start and stop dates and reason for use.

The following medication or medication containing any of the following substance should not be taken immediately prior to or during the study period:

- Antidepressant
- Antihistamine
- Benzodiazepine or derivative
- Narcotics
- Antibiotics or other antimicrobial
- Gestagens for menstruation repression (unless the patient has regular menstruation or if used for contraception)
- Oral iron preparation or iron containing multivitamins

If any of the above-listed medication was taken prior to the study, a washout period corresponding to 5 times the half-life of the compound should be applied prior to baseline assessments.

Investigator shall ensure that patient has not taken caffeine containing drink or nicotine product at least 1 hour prior to the fatigue or cognitive function assessments. Patient should refrain from consuming alcoholic drink with 12 hours prior to testing.

8. RISKS/PRECAUTIONS

Parentally administered iron preparations can cause hypersensitivity reactions (see Section 2, Rationale, page 23). Therefore, facilities for cardio-pulmonary resuscitation must be available.

Parenteral iron must be used with caution in cases of acute or chronic infection, asthma, eczema, or atopic allergies.

Caution should be exercised to avoid paravenous leakage when administering FCM. Paravenous leakage at the injection site may lead to brown discolouration and irritation of the skin. In case of paravenous leakage, the administration should be stopped immediately.

One millilitre of undiluted FCM contains up to 0.24 mmol (5.5 mg) of sodium. This has to be taken into account in patients on sodium-controlled diet.

In case of accidental overdose, patient shall be monitored for iron overload.

9. STUDY PROCEDURES

9.1 Description of Study Assessments

9.1.1 Efficacy Assessments

9.1.1.1 Patient Report Outcome and Screening Questionnaires

Patient report outcome is an umbrella term applicable to any health care data reported by the patient without interpretation by the physician/Investigator or other health care giver about how the patient feels in relation to a health condition and its therapy. In this study the primary clinical efficacy of FCM will be assessed using the PFS, a validated self-administered research instrument which is relevant to this study population. The SF-12 (version 2) relates to general health and overall well-being. The RLS diagnostic criteria and the RLS rating scale are validated instruments to confirm the RLS and to measure severity, respectively. The SF-12 questionnaire will provide data to support and confirm findings of the primary efficacy endpoint. If RLS is confirmed at screening using the validated diagnostic criteria, the RLS rating scale will then be performed at every subsequent visit. Results from the sub-group of RLS-confirmed patients will be analysed as secondary endpoints.

Finally, the PHQ (see Appendix 5.1) is not a PRO but a screening tool for Major Depressive Disorder. It is a self-administered questionnaire but unlike the PRO the Investigator will be required to evaluate the results to determine whether the patient is eligible. A score equal or greater than 2 on 5 items, one of which corresponds to question number 1 or 2 will preclude the patient from participation in the study.

As far as possible, at each visit the patient will complete the forms before any other assessments or interviews are carried out, so that the answers will not be influenced by the Investigator or other study procedures.

Original English version of all study-related questionnaires are appended to this protocol (Appendices 5). Investigator will be provided with adequately validated questionnaires in the following languages: German, French and Swedish.

Piper Fatigue Scale (Appendix 5.2)

The revised PFS questionnaire will be used at screening to confirm patient eligibility. A minimum total score of 5 is required at screening to be eligible for this study. The PFS will also be performed at every study visit.

The PFS in its current form is composed of 22 numerically scaled, "0" to "10" items that measure 4 dimensions of subjective fatigue:

- Behavioural/severity (6 items; numbers 2-7)
- Affective meaning (5 items: numbers 8-12)

- Sensory (5 items: numbers 13-17), and
- Cognitive/mood (6 items: numbers 18-23)

These 22 items are used to calculate the 4 sub-scales dimensional scores and the total fatigue scores. Instruction for calculating the total score are provided in Appendix 5.2.

Five additional items (number 1 and numbers 24-27) are not numerically scaled but need to be answered by the patient. These are not used to calculate subscale or total fatigue scores but are recommended to be kept on the scale as these items furnish rich, qualitative data. Item 1, in particular gives a categorical way in which to assess the duration of the respondent's fatigue.

SF-12 (Appendix 5.3)

The SF-12 Health Survey is a shorter version of SF-36. It utilises only 12 items drawn from each of the 8 subscales of the SF-36. The performance of the SF-12 has been reported to be comparable to that of the SF-36 while having the advantage of being easier and taking 2-3 minutes to complete. The SF-12 physical and mental health summary measures are referred to physical component summary-12 and mental component summary-12, respectively.

The SF-12 Health Survey is published in standard (4 week) and acute (1 week) recall versions for self-administration. In this study the acute 1-week recall version will be used. Each item must be answered by patient on a 5-choice response scale. There is no minimal score required for eligibility. The SF-12 will be performed at baseline before treatment and at the end of the study.

- Functional status
  - Physical functioning x 2 items
  - Social functioning x 1 item
  - Role limitations attributable to physical problems x 2 items
  - Role limitations attributable to emotional problems x 2 items
- Well-being
  - Mental health x 2 items
  - Energy and fatigue x 1 item
  - Pain x 1 item
- Overall evaluation of health
  - General health perception x 1 item

Restless Leg Syndrome (Appendices 5.4a and 5.4b)

The association between brain ID and RLS is well established (see Section 1, Introduction and Background, page 17). This syndrome is often overlooked and because of the high prevalence in subject with an s-ferritin <50 ng/mL, the Investigator will apply the diagnostic criteria (Appendix 5.4a) as part of the medical history. The presence of RLS is not required for entry. However, if confirmed the RLS rating scale shall be applied at baseline and every visit thereafter.

9.1.1.2 Cognitive Function Assessments (Appendix 5.5)

The CDR computerised cognitive assessment system will be used to assess cognitive function. A selection of tasks from the CDR computerised cognitive assessment system will be administered, parallel forms of the tests being presented on each testing session. All tasks are computer-controlled, the information being presented on high resolution screens, and the responses recorded via a response module containing 2 buttons, 1 marked 'NO' and the other 'YES'. In the word recall tasks the patient writes down the words on a sheet of paper. The test battery takes about 20-25 minutes to perform. The tests are administered in the following order:

- Immediate Word Recall: A list of 15 words is presented on the screen at the rate of 1 every 2 seconds for the patient to remember. The patient is then given 1 minute to recall as many of the words as possible.

- Picture Presentation: A series of 20 pictures is presented on the screen at the rate of 1 every 3 seconds for the patient to remember. No data are recorded from this task.

- Simple Reaction Time: The patient is instructed to press the 'YES' response button as quickly as possible every time the word 'YES' is presented on the screen. Thirty stimuli are presented with a varying inter-stimulus interval.

- Digit Vigilance Task: A target digit is randomly selected and constantly displayed to the right of the screen. A series of digits is then presented in the centre of the screen at the rate of 150 per minute and the patient is required to press the 'YES' button as quickly as possible every time the digit in the series matched the target digit. There are 45 targets. The task lasts for about 3 minutes.

- Choice Reaction Time: Either the word 'NO' or the word 'YES' is presented on the screen and the patient is instructed to press the corresponding button as quickly as possible. There are 30 trials for which each stimulus word is chosen randomly with equal probability and there is a varying inter-stimulus interval.

- Spatial Working Memory: A picture of a house is presented on the screen with 4 of its 9 windows lit. The patient has to memorise the position of the lit windows. For each of the 36 subsequent presentations of the house, the patient is required to decide whether or not the 1 window that was lit was also lit in the original

presentation. The patient responds by pressing the 'YES' or 'NO' response button as appropriate.

- Numeric Working Memory: A series of 5 digits is presented for the patient to hold in memory. This is followed by a series of 30 probe digits for each of which the patient has to decide whether or not it was in the original series and press the 'YES' or 'NO' response button as appropriate.

- Delayed Word Recall: The patient is again given 1 minute to recall as many of the words as possible.

- Word Recognition: The original words plus 15 distractor words are presented 1 at a time in a randomised order. For each word the patient is required to indicate whether or not the patient recognises it as being from the original list of words by pressing the 'YES' or 'NO' button as appropriate, as quickly as possible.

- Picture Recognition: The original pictures plus 20 distractor pictures are presented 1 at a time in a randomised order. For each picture the patient has to indicate whether or not the patient recognises it as being from the original series by pressing the 'YES' or 'NO' button as appropriate.

Additional Assessments

- Bond-Lader visual analogue scale of mood and alertness: This questionnaire of 16 analogue scales derives 3 factors that assess change in self-rated alertness, self-rated calmness and self-rated contentment. It has proven sensitivity to a wide range of compounds. A computerised version of this test is employed, with the patient using the computer mouse (see Appendix 5.5).

CDR TESTING SCHEDULE

Training

Training on the CDR system and the Visual Analogue Scales will take place prior to the first day of dosing of the trial in order to ensure an optimal level of performance for the baseline assessment on the first study day. Training helps overcome initial test anxiety, familiarises the patients with the procedures, enables the development of strategies for task performance and overcomes any initial practice effects. Two training sessions will be completed by each patient prior to the start of the study. If a patient is unable to perform 3 or more of the CDR tasks to the specified level (age-matched normative data) then the patient shall be excluded from the study.

Study Days

The CDR system test battery will be administered at pre-dose on Day 0, Day 7, Day 28 and Day 56 (or at the early termination visit).

Patients should not smoke, or drink caffeine containing drinks for 1 hour prior to testing. Patient should refrain from drinking alcoholic beverage 12 hours prior to testing.

9.1.2 Safety Assessments

9.1.2.1 Laboratory Parameters

Blood samples will be taken using standard venipuncture techniques. Laboratory assessments will be performed in local or regional laboratory used at each site.

Before starting the study, the Investigator will supply the Sponsor with a list of the normal ranges and units of measurement for each site.

The serum iron status parameters ferritin, transferrin, and TfS and soluble transferrin receptors will be determined in accordance with the Schedule of Events, page 10.

The following clinical laboratory parameters will be determined in accordance with the Schedule of Events, page 10.

Haematology

Haemoglobin, haematocrit, RBC count, reticulocytes, white blood cell count with differential and platelet count, mean corpuscular volume, mean corpuscular haemoglobin, erythrocyte sedimentation rate.

Biochemistry

Alanine aminotransferase, AST

Phosphate

Urinalysis

With phosphate in spontaneous urine

Screening Only

C-reactive protein

Thyroid stimulating hormone

Vitamin B_{12} and folic acid

Serum pregnancy test (a urine pregnancy test will be required at End of Study visit)

9.1.2.2 Vital Signs

- Axillary or oral temperature (same route should be used throughout)

- Resting blood pressure (systolic and diastolic) and heart rate will be measured in a sitting position

- For timing of assessments refer to the Schedule of Events, page 10

9.1.2.3 Physical Examination

Body systems to be assessed as part of the physical examination should include:

- General appearance
- Head (eyes, ears, nose and throat)
- Cardiovascular
- Respiratory
- Abdomen
- Urogenital
- Musculoskeletal
- Neurological
- Lymph nodes
- Skin

For timing of assessments refer to the Schedule of Events, page 10.

9.2 Schedule of Events

After signing the informed consent baseline assessments will be performed within 14 days of screening visit. At baseline visit (Day 0) all eligibility criteria should be confirmed. Patient will be randomised and treated on "Day 0". Study procedures will be performed and data collected as per Schedule of Events, page 10.

9.2.1 End of Treatment (or Early Discontinuation) Procedures

On completion of treatment (or if subject is withdrawn early) all assessments required on Day 56 per Schedule of Events, page 10 should be completed.

9.2.2 Follow-up Procedures

A follow-up call will be made 2 weeks after the last study visit to record the status of any ongoing AEs from Visit 5. A follow-up visit may be scheduled only if additional laboratory or clinical assessments are deemed necessary.

10. EVALUATION, RECORDING AND REPORTING OF AES AND SAES

10.1 Definitions

10.1.1 Adverse Event

Any untoward medical occurrence in a subject or clinical investigation subject administered a pharmaceutical product and which does not necessarily have a causal relationship with this treatment. An AE can therefore be any unfavourable and unintended sign (including an abnormal laboratory finding), symptom, or disease temporally associated with the use of a medicinal (investigational) product, whether or not related to the medicinal (investigational) product.

10.1.2 Adverse Drug Reaction

In the pre-approval clinical experience with a new medical product or its new usages, particularly as the therapeutic dose(s) may not be established, all noxious and unintended responses to a medicinal product related to any dose should be considered adverse drug reactions (ADRs). The phrase “responses to a medicinal product” means that a causal relationship between a medicinal product and an AE is at least a reasonable possibility, i.e., the relationship cannot be ruled out.

10.1.3 Unexpected AE/ADR

An AE/ADR, the nature (i.e., specificity/seriousness/outcome/frequency) or severity of which is not consistent with the applicable product information (e.g., Investigator’s Brochure for an unapproved investigational product, or Package Insert/Summary of Product Characteristics for an approved product).

10.1.4 Serious Adverse Event

An SAE is defined as any untoward medical occurrence that at any dose:

- Results in death
- Is life-threatening (the term “life-threatening” in the definition of “serious” refers to an event in which the subject was at risk of death at the time of the event; it does not refer to an event which hypothetically might have caused death if it was more severe)
- Requires inpatient hospitalisation or prolongation of existing hospitalisation
- Results in persistent or significant disability/incapacity
- Is a congenital anomaly/birth defect
- Is an important medical event (i.e., medically significant)

Medical and scientific judgment should be exercised in deciding whether expedited reporting is appropriate in other situations, such as important medical events that may not be immediately life-threatening or result in death or hospitalisation but may jeopardise the subject or may require intervention to prevent one of the other outcomes listed in the definition above. These should also usually be considered serious.

Examples of such events are intensive treatment in an emergency room or at home for allergic bronchospasm; blood dyscrasias or convulsions that do not result in hospitalisation; or development of drug dependency or drug abuse.

Conversely, some hospitalisations, particularly those which are the result of elective or previously scheduled surgery for pre-existing conditions, which have not worsened after initiation of treatment, should not automatically be classed as SAEs. For example, an admission for a previously scheduled ventral hernia repair would not be classed as an SAE. Previously scheduled hospitalisations must be documented in the subject's source documents before the subject signed the informed consent form (ICF).

Any suspected transmission of any infectious agent via a medicinal product should be considered as an important medical event (i.e., medically significant) and therefore documented as an SAE.

10.1.5 Suspected Unexpected Serious Adverse Reaction

Any ADR that is both serious and unexpected (per the reference safety information) that, based on the opinion of the Investigator or Sponsor, is felt to have a reasonable suspected causal relationship to a medicinal product.

10.2 Adverse Event Descriptors

10.2.1 Intensity/Severity Categorisation

The term "severe" is often used to describe the intensity (severity) of a specific event (as in mild, moderate, or severe myocardial infarction); however the event itself may be of relatively minor medical significance (such as severe headache). This is not the same as "serious," which is based on subject/event outcome or action criteria usually associated with events that pose a threat to a subject's life or functioning. Seriousness (not severity) serves as a guide for defining regulatory reporting obligations.

In general, the intensity of a particular AE to be recorded is the worst intensity experienced by the subject during the course of the event. The medical assessment of intensity will be determined by using the following definitions:

Mild: The AE is easily tolerated and does not interfere with usual activity.

Moderate: The AE interferes with daily activity, but the subject is still able to function.

Severe: The AE is incapacitating and the subject is unable to work or complete usual activity.

10.2.2 Causal Relationship Categorisation

An Investigator who is qualified in medicine (or dentistry, if appropriate) must make the determination of relationship to investigational product for each AE and SAE. The Investigator should decide whether, in his or her medical judgement, there is a reasonable possibility that the event may have been caused by the investigational product. If there is no valid reason for suggesting a relationship, then the AE/SAE should be classified as not related. Otherwise, if there is any valid reason, even if undetermined or untested, for suspecting a cause-and-effect relationship between the investigational product and the occurrence of the AE/SAE, then the AE/SAE should be considered related. For SAEs, the Investigator must provide a brief comment explaining the rationale of his/her assessment of causal relationship on the SAE Reporting Form.

The following additional guidance may be helpful:

Term	Relationship	Definition
Related	Yes	The temporal relationship of the clinical event to trial drug administration indicates a causal relationship, and other drugs, therapeutic interventions or underlying conditions do not provide a sufficient explanation for the observed event.
Not related	No	The temporal relationship of the clinical event to trial drug administration does not indicate a causal relationship, or other drugs, therapeutic interventions or underlying conditions provide a sufficient explanation for the observed event.

If the causal relationship between an AE/SAE and the investigational product is determined to be ‘related’, the event will be considered to be related to investigational product for the purposes of expedited regulatory reporting. In circumstances where the Investigator is not providing his/her assessment about the relationship, the event will be considered as ‘related’ and qualify for expedited regulatory reporting.

10.2.3 Outcome Categorisation

Outcome may be classified as resolved without sequelae; unresolved; resolved with sequelae; fatal or unknown. If the outcome is reported as resolved with sequelae, the Investigator should specify the kind of sequelae on the SAE report form.

10.2.4 Symptoms of the Disease Under Study

Symptoms of the disease under study should not be classed as AEs as long as they are within the normal day-to-day fluctuation of the disease.

Worsening of the symptoms however, should be recorded as an AE, and clearly marked as worsening.

10.2.5 Clinical Laboratory Evaluations

A change in the value of a safety laboratory investigation can represent an AE if the change is clinically relevant or if, during treatment with the investigational product, a shift of a parameter is observed from a normal value to a pathological value, or a further worsening of an already pathological value. When evaluating such changes, the extent of deviation from the reference range, the duration until return to the reference range, either while continuing treatment or after the end of treatment with the investigational product, and the range of variation of the respective parameter within its reference range, must be taken into consideration.

If, at the end of the treatment phase, there are pathological laboratory values which were not present at baseline, further clinical or laboratory investigations should be performed until the values return to within reference range or until a plausible explanation (e.g., concomitant disease) is found for the pathological laboratory values.

The Investigator should decide, based on the above criteria and the clinical condition of a subject, whether a change in a laboratory parameter is clinically significant and therefore represents an AE. If the Investigator considers such an AE as serious (e.g., medically significant event fulfilling criteria per Section 10.1.4, Serious Adverse Event, page 44) it must be reported as an SAE.

At the end of the study period all pathological laboratory findings/values diagnosed throughout the treatment period should be reviewed by the Investigator to provide a final clinical assessment in view of the dynamic of laboratory changes/abnormalities.

10.2.6 Abuse, Misuse, Overdose and Medication Error

All special events such as study medication abuse, misuse, overdose and medication error have to be documented in the subject’s CRF. If any abuse, misuse, overdose, and medication errors leads to any event that fulfils any seriousness criteria (see Section 10.1.4, Serious Adverse Event, page 44), the event has to be reported as an SAE.

10.3 Reporting Procedure for AEs, SAEs and Pregnancy

10.3.1 Adverse Events

All AEs either observed by the Investigator or one of his/her medical collaborators, or reported by the subject spontaneously, or in response to a direct question, will be noted in the AE section of the subject's CRF and source document. This applies to all AEs regardless of presumed relationship to the study treatment, and to the

investigational product, active comparator, placebo and no treatment arms. Adverse events leading to discontinuation of study treatment should also be collected.

If any AE is reported, the date of onset, relationship to study medication or treatment, any action taken, date of resolution (or the fact that it is still continuing or has become chronic), outcome, and whether the AE is serious or not will be recorded. Where possible, the Investigator should report a diagnosis rather than signs and symptoms or abnormal laboratory values.

The AE reporting period begins at the time the ICF is signed by the subject. The AE reporting period ends at the last study visit (Visit 5/Day 56). Adverse events persisting at the time of study completion will be followed by the Investigator through contact with the subject until resolution or stabilisation has occurred.

If the subject reports an AE, it is the Investigator's responsibility to acquire sufficient information in order to assess causality. This may require additional laboratory testing, physical examinations, telephone contacts, etc.

In order to avoid bias in eliciting AEs, subjects should be asked a non-leading question, such as "How are you feeling?" It is also important to question the subject in a non-leading way about changes in their health or concomitant medication usage since their last visit. This information should be collected prior to completion of assessments at all study visits. In addition, any symptoms/conditions reported during assessments and deemed to be clinically significant by the Investigator will be assessed as AEs.

10.3.2 Serious Adverse Events

The occurrence of an SAE must be reported to the Sponsor immediately (within 24 hours of awareness) by email safety@viforpharma.com to Vifor Pharma (or its delegate; e.g., Contract Research Organisation (CRO)) safety contacts listed below. The SAE should be reported using the Vifor Pharma SAE Reporting Form provided by Vifor Pharma. The Investigator must complete, sign and date the SAE pages, and verify the accuracy of the information recorded on the SAE pages with the corresponding source documents. The Vifor Pharma SAE Reporting Form must be completed in capital letters, in medical terms, in English and to the best extent possible given the time constraints. Where possible, the Investigator should report a diagnosis rather than signs and symptoms. Where the SAE Reporting Form cannot be transmitted due to technical problems, the Investigator must inform the CRO/Vifor Pharma about the SAE by phone. As soon as technical problems are resolved, the Investigator will send a copy of the SAE Reporting Form to the CRO/Vifor Pharma.

All SAEs ('related' or 'not related') will be reported from the time the informed consent is signed until 30 days (or the appropriate pharmacokinetic equivalent of five half-lives, whichever is longer) following the last visit date. The onset date of the AE is defined as the onset of signs and symptoms or a change in baseline. The onset date of the SAE is defined as the date the signs and symptoms/diagnosis became serious,

i.e., met at least 1 of the International Conference on Harmonisation (ICH) criteria for ‘serious’. The resolution date of the SAE is defined as when the symptoms resolve, or the event is considered chronic (e.g., sequelae) or stable. Serious adverse events that are ongoing events at the time of death are considered unresolved. All recorded SAEs, regardless of relationship to investigational product, will be followed up until resolution, stabilisation, or the subject is lost to follow-up and cannot be contacted. In circumstances where the Investigator is unable to make contact with the subject, the Investigator must provide a written statement to the CRO/Vifor Pharma, confirming that the subject is lost to follow-up.

Any SAE considered to have a ‘causal relationship’ (i.e., ‘related’) to the investigational product and discovered by the Investigator at any time after the study should be reported. A rationale for the assessment of a causal relationship must be provided by the Investigator together with the Vifor Pharma SAE Reporting Form. Any safety information that is obtained after database lock of the clinical database will be documented only in the safety database.

A death occurring during the study or which comes to the attention of the Investigator within 30 days after the last study visit, whether considered treatment-related or not, must be reported to the CRO/Vifor Pharma using the Vifor Pharma SAE Reporting Form. Preliminary reports will be followed by detailed descriptions which will include copies of hospital case reports, autopsy reports/certificates and other documents when requested and applicable.

Contact information for reporting of SAEs:

SGS Life Sciences Services
Clinical Research
Generaal De Wittelaan 19A bus 5
B-2800 Mechelen
Belgium.

At a minimum the following should be provided at the time of the initial SAE report:

- Study name and/or number
- Subject number, date of birth, gender/sex and initials
- Event description (including onset date of the event, outcome and reason for it being considered serious)
- Relationship to investigational product (i.e., causality)
- Name of the investigational product (including drug dose and administration dates)
- Investigator name and address

- Name of the reporter (including site name or number and country), and
- Dated signature of the Investigator or Sub-/Co-investigator.

Additional follow-up information, if required or available, must be faxed immediately (within 24 hours of awareness) following Investigator (or site) awareness of the information. The follow-up information must be completed on a Vifor Pharma SAE Reporting Form (marked follow-up) and placed with the original in the appropriate section of the study file.

The Investigator is encouraged to discuss with CRO/Sponsor any AEs for which the issue of seriousness is unclear or questionable.

Vifor Pharma, or its delegate, is responsible for expedited reporting to the relevant regulatory authorities, to Investigators and to local and central Institutional Review Board (IRB)/Ethics Committee (EC)/Independent Ethics Committee (IEC) as per local regulations.

Additional detail regarding SAE reporting is available in the SAE Reporting Procedure document, which will be provided in the study documentation.

10.3.2.1 Elective Surgery/Routine Examination

Elective surgery (a planned, non-emergency medical procedure) and in-patient routine examination for a pre-existing condition do not qualify as SAEs. However, AEs which occur during the elective hospitalisation will need to be collected and reported.

10.3.3 Pregnancy

The safety of study treatment in pregnant women needs to be monitored once study treatment has been administered to them (as per protocol, pregnancy is an exclusion criteria). Therefore, the outcome of all such pregnancies (including normal births) must be followed up and documented, even if the subject was withdrawn from the study.

Women of child bearing potential should have a negative serum pregnancy test with a sensitivity of at least 50 mIU/mL hCG within 1 week prior to beginning study medication. It is recommended that study medication should not be initiated by the Investigator until a report of a negative pregnancy test has been obtained.

Effective contraception must be used before beginning study medication, during study dosing, and for 30 days following discontinuation of study medication, even when there has been a history of infertility, unless due to hysterectomy. Please refer to the exclusion/inclusion criteria (Section 5, Selection and Withdrawal of Patients, page 29) for additional information.

A female subject must immediately inform the Investigator if she becomes pregnant during the study. The Medical Monitor must be contacted immediately to break the

blind (if applicable). The Investigator should counsel the subject and discuss the risks of continuing with the pregnancy and the possible effects on the foetus. The Investigator/Sponsor is responsible for monitoring the subject and pregnancy outcome until 90 days postpartum (or otherwise as appropriate). The female partner of a male subject should also be counselled and followed-up as described above.

Any report of pregnancy recorded for any female subject or for a female partner of a male subject should be reported to the CRO/Vifor Pharma as though it were an SAE, i.e., immediately (within 24 hours of awareness). The Investigator should complete a Vifor Pharma Pregnancy Report Form and forward to the CRO/Vifor Pharma. Complications of pregnancy such as abortion (spontaneous or induced), premature birth or congenital abnormality are considered SAEs and should be reported using the Vifor Pharma SAE Reporting Form.

All pregnancies occurring in a female subject or the female partner of a male subject within 90 days after discontinuation of investigational product should be reported as an SAE to the CRO/Vifor Pharma.

Please also refer to Section 5.2, Inclusion Criteria, page 29, and Section 5.3, Exclusion Criteria, page 30, for inclusion and exclusion criteria.

11. STUDY VARIABLES AND STATISTICAL ANALYSIS

11.1 Statistical Methods

11.1.1 Populations

Safety set: The safety set/population consists of all volunteers who were randomised, taken at least 1 dose of study treatment (regardless of amount of drug) and are analysed by the treatment taken.

Intent-to-treat (ITT) set: All subjects who were randomised and dispensed at least 1 dose of study medication (regardless of amount of drug) and have at least 1 post-baseline efficacy measurement. Hereby, treatment group is defined by randomisation (and not by real treatment, "treated as randomised").

Per-protocol (PP) set: All subjects without any major protocol violation and with available values of the relevant study variables in all periods will be included in the PP population. Hereby, treatment group is defined by randomisation (and not by real treatment, "treated as randomised").

A confirmatory analysis will be based on the ITT population. Analysis will be repeated for PP population. Differences in study results between both analysis populations in regard of primary endpoint will be addressed in the study report.

A complete listing of protocol violations, their classification and the assignment of volunteers to analysis populations will be signed by the Sponsor before data base lock.

11.2 Background and Demographic Characteristics

Descriptive statistics of background and demographic characteristics will be presented in tabular format.

In case of variables with continuous scaled data, mean, standard deviation, standard error, minimum, maximum, median and interquartile range will be presented by time point and treatment group. If variable is included in statistical analysis of primary or secondary endpoint, deviation from normal distribution is evaluated with Kolmogoroff-Smirnow test. Hereby, a $p<0.1$ is indicating deviation.

In case of variables with categorically scaled data, absolute and relative frequencies will be presented.

11.3 Study Medication

Following characteristics of study medication will be presented as absolute and relative frequencies: Therapy (verum/treatment), treatment in coincidence with randomisation, dose, correct dose, days between study medication and visits.

11.4 Medical History and Concomitant Diagnoses and Therapy

Medical history and concomitant diagnoses or therapies will be presented by treatment as absolute and relative frequencies of patients' organ class and preferred term according to the Medical Dictionary for Regulatory Activities.

Previous therapies and concomitant medications taken at any stage during the study will be presented by treatment as absolute and relative numbers of patients taking medications in each anatomic therapeutic class according to the WHO drug dictionary.

11.5 Efficacy Evaluations

Descriptive statistics of any collected as well as derived variable which are in relationship to primary or secondary endpoints will be presented in a tabular format.

Continuously scaled data will be analysed as described in Section 11.5.2, Secondary, page 54. In addition, variables will be presented in boxplots by time and treatment group.

In case of variables with categorically scaled data, absolute and relative frequencies will be presented.

11.5.1 Primary

Number of responders (patients with improvement of total sum of PFS $\geq$1) and non-responders (patients with decrease or improvement of total sum of PFS <1) will be presented in a 2 x 2 table, whereby table contains expected values, too. In addition, fraction of responders within both treatment groups (verum group: P_{Verum}, placebo group: $P_{Placebo}$) will be given together with their exact 95% confidence intervals (CIs). Finally, difference of fractions will be provided.

Two bar charts, 1 for absolute values, 1 for fractions, will be used for graphical presentation.

Difference in fractions of responders is analysed via χ^2–test (two-sided, alpha-level: 0.05) using contingency table mentioned above. It should be noted, that decision for two-sided approach is based on recommendation of guidelines for statistical analysis of controlled clinical trials.

For confirmatory analysis, following null hypothesis is used:

$$H_0: \{P_{Verum}=P_{Placebo}\}.$$

If an effect of a larger fraction of responders in verum group is found, and null hypothesis is rejected at 0.05 level, a significant elevated efficacy of study treatment medication compared with placebo medication can be concluded.

As result of χ^2-test, χ^2, degrees of freedom and p-value will be presented.

Because response might be influenced by covariates as, for example, baseline PFS or centre effects, Section 11.8.2, Assessment of Interactions, Covariates, Centre Effects, page 57 describes application of generalised linear models (GLM) as well as generalised linear mixed model (GLMM) in order to assess effect of treatment and additional covariates on response in an exploratory way. If important covariates are identified, result of χ^2-test will be discussed in conjunction with these findings.

11.5.2 Secondary

From statistical point of view, secondary endpoints can be distinguished into (i) comparison of fractions (secondary endpoints 1, 4, 11), (ii) comparison of expected values (mean, median) of continuously scaled variables (secondary endpoints 2-3, 5-10) and (iii) correlation analysis (secondary endpoints 12-13).

(i) Contingency table presenting absolute frequencies by investigated factor (rows) and by treatment (columns) will be presented. Column-wise fractions are given together with 95% confidence interval (CI). Difference of fraction is given. Within an explorative analysis, two-sided χ^2- test is performed, and χ^2, degrees of freedom and p-value will be indicated.

(ii) Results regarding difference of mean changes are presented in dependence of the distribution.

(iia) In case of no deviation from normal distribution, mean, standard deviation, standard error a 95% CI of mean changes as well as their difference is given. Hereby, variance estimates of difference are calculated by usual rules. Two-sided t-test for unpaired samples will be applied in exploratory manner to test whether difference is different from 0.

(iib) In case of deviation from normal distribution, median, interquartile range and minimum, maximum of changes versus baseline are presented. Median difference of changes is presented by Hodges-Lehman estimate and its 95% CI. Mann Whitneys U test is applied in exploratory manner to test whether difference is different from 0.

In both cases, box-whisker plots are used for graphical presentation of changes by treatment.

(iii) In dependence on distribution of both variables, results (correlation coefficient r (Pearson correlation) or ρ (Spearmann rank correlation), p-value for its difference from 0) of Pearson correlation (both variables do not deviate from normal distribution) or Spearman rank correlation (at least 1 of the variables show deviation from normal distribution) are presented, whereby analysis is performed per treatment group. If appropriate, a correlation matrix can be used to summarise results. Overlay scatterplots of variables by treatment data are presented.

Within analyses of secondary endpoints, significance level is set at an alpha of 0.05 and no adjustment will be made for testing multiple secondary outcomes. Some significant findings are expected to occur by chance so undue consideration will not be given to any particular significant difference. Moreover, interpretation of the results will be based on patterns of differences and in conjunction with the results of the primary analyses.

11.6 Safety Evaluations

Adverse events and severe adverse events (SAE) will be summarised by presenting following safety parameters by absolute and relative frequencies of patients by treatment:

Serious Adverse Event

- Description (system organ class (SOC))
- Classification (SOC)
- Ongoing (y/n)
- Death (y/n)
- Life threatening (y/n)
- Involved or prolonged inpatient hospitalisation (y/n)
- Involved persistence of significant disability or incapacity (y/n)
- Congenital abnormality/birth defect, important medical event (y/n)
- Causality (unrelated, unlikely related, possibly related, probably related, certainly related)
- Outcome of AE (resolved without sequelae, resolved with sequelae, not yet recovered, death, lost to follow-up)
- Action taken (none, study drug discontinuation temporarily, study drug discontinuation permanently, remedial drug therapy)

Adverse Event, Whereby each SAE is also Documented as an AE

- Serious (y/n)
- Description (SOC)
- Classification (SOC)
- Discontinuation from the study (y/n)

- Temporary discontinuation from the study (y/n)
- Require treatment (y/n)
- Causality (no: unrelated, unlikely related, yes: possibly related, probably related, certainly related)
- Start date/time
- End date/time
- Ongoing (y/n)
- Intensity (mild, moderate, severe)
- Outcome (resolved without sequelae, resolved with sequelae, not yet recovered, death, lost to follow-up)
- Action taken (none, study drug discontinuation temporarily, study drug discontinuation permanently, remedial drug therapy)

11.7 Interim Analyses

Not applicable here.

11.8 Other Topics

11.8.1 Missing Values

Two types of missing values could occur within study. First, values for single items of PFS can be missing. Second, entire measurements can be missed.

If less than 25% of PFS items are missing for a patient, the total PFS score is calculated on base of remaining items, and missing items are replaced by the mean of the other items. If more than 25% of items are missing, the entire value is considered to be missing.

If entire values are missed, different strategies will be applied, and sensitivity of conclusions in regard of these different strategies is investigated. Hereby only missing values in terms of primary efficacy endpoint (missing total score PFS) are addressed by imputation methods, whereby several procedures are applied.

Primary analysis uses LOCF (last observation carried forward) rule. In addition, a likelihood-based, mixed effects repeated measures analysis of difference of fractions (without imputation of missing values) is performed (SAS procedure PROC GLIMMIX). Finally, a worst case scenario is applicable, regarding all verum-group patients with missing values as non-responders and all placebo-group patients as responders.

Analysis of sensitivity of conclusion in dependence on method assesses:

- Spectrum of results
- Whether “missing completely at random” condition is fulfilled (only than LOCF rule is applicable)

Final methodology of handling of missing entire data is fixed in the statistical analysis plan (SAP) after blinded review of data.

11.8.2 Assessment of Interactions, Covariates, Centre Effects

Generalised linear models (here logistic regression) and generalised linear mixed models (GLMM) are applied to assess influence of covariates and interactions. Moreover, subgroups can be assessed by regarding the whole dataset and introducing subgroup-describing covariate as a factor into the model.

It might be the case, that a positive study result can only be achieved when data are assessed by GLM or GLMM. In that case, the result is valid, but have only explorative character.

11.8.3 Pharmacokinetics

Not applicable.

11.8.4 Quality of Life

Not applicable.

11.9 Sample Size and Power Calculations

General Considerations

Sample size calculation refers to number of subjects required for the primary analysis.

Sample size estimation requires assumptions about effect, variation among patients, probabilities of Type I error (alpha-level) and Type II error (100% power) and should consider factors leading to drop-outs, withdrawals and missing values.

Main aspect is the effect, which is here the difference in fractions of patients who respond. Assumptions for this difference are yielded from a (i) general request on efficacy of a fatigue-related treatment as well as from (ii) analyses of previous trial [34].

As a result of an expert panel discussion, we consider a 15-20% difference of responders between iron treatment and placebo treatment as an amount, which would justify a treatment of fatigue.

Applying inclusion criteria (s-ferritin <50 ng/mL plus TfS <20% or s-ferritin <15 ng/mL) of this trial to the population of FERRIM trial, a sub-population (verum:

n=22, placebo: N=19) is found which shows a significant difference in change of mean total score of BFI (-0.89; 95% CI: -0.22 to -1.89), p=0.022). It should be noted that an improvement of BFI is connected with a decreased total score. Moreover, using a similar response criterion (decrease of 1 unit on a 10-unit), 59.1% (13/22) versus 31.6% (6/19) of patients (Fisher's exact test: p=0.12) were identified as responders. In other words, a 27.5% difference was found. Because of low sample size, difference could not be shown to be significant.

Based on previous result of a similar clinical trial (reduced by 10%) which used other iron compound as well as other PRO measure, a minimum difference of 17% was regarded to be appropriate [12].

For Type I and Type II error, conventionally values of 5% and 20% (power: 80%) are chosen.

Calculation

Software n-query (Statistical solutions) is used. A two-group χ^2-test with a 0.050 two-sided significance level will have 80% power to detect the difference between placebo verum group proportion, $P_{Placebo}$, of 0.500 and verum group proportion, P_{Verum}, of 0.670 when the sample size in each group is 131.

If we assume drop-out rate of around 10% or less, a total 144 cases per treatment group need to be randomised.

11.10 Statistical Software

SAS 9.2.

12. STUDY ETHICAL CONSIDERATIONS

12.1 Ethical Conduct of the Study

The study will be conducted according to the principles of the World Medical Association's (WMA) Declaration of Helsinki (as amended by the 59th WMA General Assembly, Seoul, October 2008), and the ICH guidelines for GCP. Vifor Pharma will ensure that the study complies with all local, federal or country regulatory requirements as applicable, such as US 21 Code of Federal Regulations (CFR) Parts 50, 54, 56, and 312. If full compliance with all declarations, guidelines and regulations is not planned, the exceptions will be noted and an explanation provided as to the acceptability of the data generated from the clinical trial.

The Investigator must ensure the anonymity of all subjects participating in the study. Each subject will be assigned a unique subject number and this should be used on all forms associated with the subject's documents or samples that will be supplied to the Sponsor or any party completing testing on behalf of the Sponsor (e.g., blood for central laboratory assessments).

All anonymous data remains the property of Vifor Pharma.

12.2 Informed Consent

The ICF used for the study must comply with the Declaration of Helsinki, federal regulations (US 21 CFR 50, 312, and ICH guidelines) and must have been approved by the IRB/EC/IEC. The Investigator or an authorised associate must explain orally and in writing the nature of the study and the treatment in such a manner that the subject is aware of potential benefits and risks. Subjects must also be informed that participation is voluntary and that they may withdraw from the study at any time, without prejudice. Subjects must be provided sufficient time to consider participation, including discussion with family members prior to signing the ICF. Documentation of the discussion and the date of informed consent must be recorded in the source documentation. Subjects must give informed consent in writing.

12.3 Institutional Review Board or EC/IEC

The protocol, any protocol amendments and consent form for the proposed clinical study and any other documents required by the local IRB/EC/IEC must be submitted by the Investigator for review and approval to the IRB/EC/IEC. The Investigator must also ensure that the IRB/EC/IEC reviews the progress of the study on a regular basis and, if necessary, renews its approval of the study on an annual basis. A copy of the approval letter must be forwarded to Vifor Pharma before the study is implemented.

12.4 Quality Control and Quality Assurance

The Investigator must ensure that all trial related sites, source data/documents, and reports will be available and that the provision of direct access for monitoring and auditing by Vifor Pharma or its designees will be permitted. In addition, all sites,

source data/documents, and reports will be made available for inspection by the appropriate Regulatory Authority and review by the IRB/EC/IEC.

Accurate and reliable data collection will be assured by verification and cross-check of the CRFs against the Investigator's records by the study Clinical Research Associate (CRA)/monitor (source document verification), and the maintenance of a drug dispensing log by the Investigator. The data collected will be entered (double-date entry) into the study database once it has been verified by the CRA/monitor. A comprehensive validation check program will verify the data and queries will be generated accordingly for resolution by the Investigator. Throughout the study, Vifor Pharma or its designates may review data as deemed necessary.

Signature by the Investigator on the affiliated Protocol Signature Page documents review, agreement and approval of the requirements contained within this protocol.

13. ADMINISTRATIVE PROCEDURES

13.1 Sponsor's Responsibilities

13.1.1 Study Supplies

Sites will be provisioned with all supplies required to manage this study. This will include but not be limited to:

- Investigator file(s) (for storage of all study related documentation)
- Study medication and infusion
- Contact list of all relevant study personnel
- Documentation and instruction for use of relevant study questionnaires
- Laptops and instruction manual for the computer-based cognitive assessments
- Case report form completion guidelines
- Study Reference Manual

13.1.2 Insurance

Vifor Pharma - Vifor (International) Inc. confirms that it carries liability insurance which protects non-employee physicians or Investigators against claims for which they may become liable as a result of damages caused by Vifor products used in clinical studies. Insurance coverage is not extended to damages that the Investigators or third parties may suffer by reason of acts of commission or omission on the part of such Investigators and that are not in accordance with accepted common medical practices (*lege artis* procedures). Vifor Pharma - Vifor (International) Inc. will reimburse the subject for all study-related injuries provided that the injury does not arise from the subject's misuse of the study drug or failure to follow the Investigator's instructions.

13.1.3 Investigator Training

All Investigators and their study personnel will receive training regarding the study procedures and GCP. This training will take place prior to enrolment of the first subject at each study centre. Each study centre will be provided with information regarding GCP and regulations specific to the conduct of clinical trials.

13.1.4 Study Monitoring

The study will be monitored by representatives of Vifor Pharma (or designee) which may include the CRO and/or partner company.

It is understood that the responsible Vifor Pharma CRA/monitor (or designee) will contact and visit the Investigator regularly and will be allowed, on request, to inspect

the various records of the trial (CRFs and other pertinent data) provided that subject confidentiality is maintained in accordance with local requirements.

It will be the CRA/monitor's responsibility to inspect the CRFs at frequent regular intervals throughout the study, to verify the adherence to the protocol and the completeness, consistency and accuracy of the data being entered on them. The CRA/monitor should have access to laboratory test reports and other subject records needed to verify the entries on the CRF. The Investigator (or his/her deputy) agrees to co-operate with the CRA/monitor to ensure that any problems detected in the course of these monitoring visits are resolved.

13.2 Investigator’s Responsibilities

13.2.1 Reporting and Recording of Data

All requested study data must be recorded legibly on the CRFs provided for the study. An explanation should be provided for all missing data. Correction of data on the CRF will be made by crossing out the incorrect entry with a single stroke and entering the correct data beside it with the initials of the individual making the correction and date of the correction. Only individuals who are identified on the authorised signature page may correct data in the CRF. For those subjects who withdraw before completion of their specified treatment regimen, all available efficacy and safety data must be entered in the CRF. The reason for withdrawal must be specified. Incomplete or inconsistent data on the CRFs will result in data queries that will be returned to the Investigator for resolution.

When using the electronic case report form (eCRF) to create, modify or maintain data, an electronic signature shall be employed to ensure the authenticity, integrity and, where appropriate, the confidentiality of the electronic records. Electronic signatures shall employ 2 distinct identification components such as an identification code and password. Signed electronic records will contain information associated with the signing such as the printed name of the signatory, the date and time when the signature was executed AND the meaning (data creation modification, review approval, maintenance) associated with the signature. Each electronic signature shall be unique to 1 individual and shall not be reused by, or reassigned to, anyone else.

Specific details for reporting and recording of safety data are as per Section 10, Evaluation, Recording and Reporting of AEs and SAEs, page 44.

13.2.2 Source Documentation

The Investigator must maintain adequate and accurate source documents upon which case reports for each subject are based. They are to be separate and distinct from CRFs, except for cases in which the Sponsor has pre-determined that direct data entry into specified pages of the subject’s CRF is appropriate. These records should include detailed notes on:

- The medical history prior to participation in the study

- The basic identifying information, such as demographics, that link the subject's source documents with the CRFs
- The results of all diagnostic tests performed, diagnoses made, therapy provided and any other data on the condition of the subject
- The subject's exposure to study treatment
- All adverse events
- The subject's exposure to any concomitant therapy (including date and quantity dispensed)
- All relevant observations and data on the condition of the subject throughout the study
- The oral and written communication with the subject regarding the study treatment (including the risks and benefits of the study). The date of informed consent must be recorded in the source documentation

13.2.3 Records Retention

Vifor Pharma may request that the Investigator must arrange for the retention of all study documentation (such as CRFs, research files, and master files) for at least 2 years after the completion or discontinuation of the study. The Sponsor will inform the Investigator in writing when files can be destroyed. Subject files and other source data must be kept for the maximum period of time permitted by the hospital, institution or private practice, but not less than 2 years. Vifor Pharma must retain all other documentation pertaining to the study for the lifetime of the product. Archived data may be held on microfiche or electronic record, provided that a back-up copy exists and that a hard copy can be generated if required.

The Investigator must inform Vifor Pharma immediately if any documents are to be destroyed, to be transferred to a different facility, or to be transferred to a different owner.

13.2.4 Site Documentation

The Investigator must maintain adequate and accurate records to enable the conduct of the study to be fully documented and the study data to be subsequently verified.

13.3 Other Services

Not applicable.

14. PROCEDURE FOR MODIFICATION OF PROTOCOL OR PREMATURE TERMINATION OF THE STUDY

14.1 Protocol Waivers, Deviations and Violations

As a general rule protocol waivers shall not be permitted.

Deviations from the protocol including violations of inclusion/exclusion criteria will be assessed as “minor” or “major” on a case-by-case basis. The criteria describing the deviation(s) and how they will be handled shall be documented in the SAP.

14.2 Protocol Amendments

Protocol amendments, except where necessary to eliminate an immediate hazard to subjects, must be made only with the prior approval of Vifor Pharma. Each IRB/EC/IEC will review and approve amendments prior to their implementation in the study. IRB/EC/IEC approval need not be obtained prior to removal of an immediate hazard to subjects.

14.3 Study Termination

Both Vifor Pharma and the Investigator reserve the right to terminate the study at any time. Should this be necessary, both parties will arrange the procedures on an individual basis after review and consultation and in accordance with the study contract. Reasons for termination may include (but are not limited to) unsatisfactory subject enrolment with respect to quality and/or quantity, site is unable to comply with the requirements of the protocol or GCP or data recording is inaccurate and/or incomplete.

In terminating the study, Vifor Pharma and the Investigator will assure that adequate consideration is given to the protection of the subject’s interests.

14.4 Protocol Amendments

All protocol amendments must be written and approved by Vifor Pharma. Each IRB or EC will review and approve amendments prior to their implementation in the study. IRB or EC approval need not be obtained prior to removal of an immediate hazard to patients.

14.5 Study Termination

Both Vifor Pharma and the Investigator reserve the right to terminate the study at any time. Should this be necessary, both parties will arrange the procedures on an individual basis after review and consultation and in accordance with the study contract. Reasons for termination may include (but are not limited to) unsatisfactory subject enrolment with respect to quality and/or quantity, site is unable to comply with the requirements of the protocol or GCP or data recording is inaccurate and/or incomplete. In terminating the study, Vifor Pharma and the Investigator will assure that adequate consideration is given to the protection of the subject’s interests.

15. POLICY FOR PUBLICATION AND PRESENTATION OF DATA

Vifor Pharma is committed to the timely communication of data from clinical research trials, following the Pharmaceutical Research and Manufacturers of America principles [48]. Where possible, authorship will be agreed at the beginning of the study. The authors will form a publication committee and this committee will propose and develop appropriate scientific manuscripts or abstracts from the study data. Investigators may not present or publish partial or complete study results individually. Any manuscript or abstract proposed by the Investigators must be reviewed and approved in writing by Vifor Pharma before submission for publication. Names of all Investigators participating in the study will be included in the publication.

The publication committee for a study will comprise of authors selected in adherence with the International Committee of Medical Journal Editors [49] criteria for authorship. That is, all authors must meet each of the following 3 criteria:

1. Substantial contribution to conception and design or acquisition of data, or analysis and interpretation of data.

2. Drafted the article or revised it critically for important intellectual content.

3. Approved the final version for publication.

Members of the study steering committee (if applicable) generally fulfil the authorship criteria through their involvement in protocol design and review, monitoring of and sometimes direct involvement with recruitment, and thus they will usually be part of the publication committee. If studies are multicentre, it may be appropriate to assign group authorship.

In addition, certain Vifor Pharma employees involved in the design and conception of the protocol, study management and data analysis and interpretation are qualified authors and will be included in the publication committee e.g., the lead physician, statistician and study project manager or their equivalents.

16. REFERENCES

1. Andrews NC. Forging a field: the golden age of iron biology. Blood. 2008 Jul 15;112(2):219-30.

2. Dunn LL, Rahmanto YS, Richardson DR. Iron uptake and metabolism in the new millennium. Trends Cell Biol. 2007;17(2):93-100.

3. WHO, UNICEF, UNU. Iron deficiency anaemia: assessment, prevention, and control. A guide for programme managers. World Health Organization 2001.

4. Feelders RA, Vreugdenhil G, Eggermont AMM, Kuiper-Kramer PA, Van Eijk HG, Swaaka JG. Regulation of iron metabolism in the acute phase response: interferon γ tumour necrosis factor α induce hypoferraemia, ferritin production and a decrease in circulating transferrin receptors in cancer patients. Eur J Clin Inv. 1998;28:520–7.

5. Guyatt GH, Oxman AD, Ali M, Willan A, McIlroy W, Patterson C. Laboratory diagnosis of iron deficiency anaemia: an overview. J Gen Int Med. 1992;7:145-53.

6. WHO. Worldwide prevalence of anaemia 1993-2005. WHO global database of anaemia. World Health Organisation. 2008.

7. Galan P, Yoon HC, Preziosi P, Viteri F, Valeix P, Fieux B, et al. Determining factors in the iron status of adult women in the SU.VI.MAX study. Eur J Clin Nutr. 1998 June;52(6):383-8.

8. Hercberg S, Preziosi P, Galan P. Iron deficiency in Europe. Public Health Nutr. 2001 Apr;4(2B):537-45.

9. Lahti-Koski M, Valsta LM, Alfthan G, Tapanainen H, Aro A. Iron status of adults in the capital area of Finland. Eur J Nutr. 2003 Oct;42(5):287-92.

10. Mansson J, Johansson G, Wiklund M, Baigi A, Marklund B. Symptom panorama in upper secondary school students and symptoms related to iron deficiency. Screening with laboratory tests, questionnaire and interventional treatment with iron. Scand J Prim Health Care. 2005 Mar;23(1):28-33.

11. Beutler E, Larsh SE, Gurney CW. Iron therapy in chronically fatigued, nonanemic women: a double-blind study. Ann Intern Med. 1960 Feb;52:378-94.

12. Verdon F, Burnand B, Stubi CL, Bonard C, Graff M, Michaud A, et al. Iron supplementation for unexplained fatigue in non-anaemic women: double blind randomised placebo controlled trial. BMJ. 2003 May 24;326(7399):1124.

13. Brownlie T 4th, Utermohlen V, Hinton PS, Haas JD. Tissue iron deficiency without anemia impairs adaptation in endurance capacity after aerobic training in previously untrained women. Am J Clin Nutr. 2004 Mar;79(3):437-43.

14. Bruner AB, Joffe A, Duggan AK, Casella JF, Brandt J. Randomised study of cognitive effects of iron supplementation in non-anaemic iron-deficient adolescent girls. Lancet. 1996 Oct 12;348(9033):992-6.

15. Patterson AJ, Brown WJ, Powers JR, Roberts DC. Iron deficiency, general health and fatigue: results from the Australian Longitudinal Study on Women's Health. Qual Life Res. 2000;9(5):491-7.

16. Patterson AJ, Brown WJ, Roberts DC. Dietary and supplement treatment of iron deficiency results in improvements in general health and fatigue in Australian women of childbearing age. J Am Coll Nutr. 2001 Aug;20(4):337-42.

17. Brutsaert TD, Hernandez-Cordero S, Rivera J, Viola T, Hughes G, Haas JD. Iron supplementation improves progressive fatigue resistance during dynamic knee extensor exercise in iron-depleted, nonanemic women. Am J Clin Nutr. 2003 Feb;77:441-8.

18. Murray-Kolb LE, Beard JL. Iron treatment normalizes cognitive functioning in young women. Am J Clin Nutr. 2007 Mar;85(3):778-87.

19. Yehuda S, Youdim MBH. Brain iron: a lesson from animal models. Am J Clin Nutr. 1989 Sep;50(3 Suppl):618-29.

20. Dallman PR. Biochemical basis for the manifestations of iron deficiency. Ann Rev Nutr. 1986;6:13-40.

21. Erikson KM, Jones BC, Hess EJ, Zhang Q, Beard JL. Iron deficiency decreases dopamine D1 and D2 receptors in rat brain. Pharmacol Biochem Behav. 2001 Jul-Aug;69(3-4):409-18.

22. Izquierdo I. Dopamine receptors in the caudate nucleus and memory processes. Trends Pharmacol Sci. 1992 Jan;13(1):7-8.

23. Oski FA. The nonhaematologic manifestations of iron deficiency. Am J Dis Childhood. 1979;133:315-22.

24. Oski FA, Honig AS, Helu B, Howanitz P. Effect of iron therapy on behavior performance in nonanemic, iron-deficient infants. Pediatrics. 1983 Jun;71(6):877-80.

25. Lozoff B, Jimenez E, Smith JB. Double burden of iron deficiency in infancy and low socioeconomic status: a longitudinal analysis of cognitive test scores to age 19 years. Arch Pediatr Adolesc Med. 2006 Nov;160(11):1108-13.

26. Robbins TW, Everitt BJ. Functional studies of the central catecholamines. Int Rev Neurobiol. 1982;23:303-65.

27. Sun ER, Chen CA, Ho G, Earley CJ, Allen RP. Iron and the restless legs syndrome. Sleep. 1998;21:371-7.

28. O'Keeffe ST, Gavin K, Lavan JN. Iron status and restless legs syndrome in the elderly. Age Ageing. 1994;23(3):200-3.

29. Norlander NB. Restless legs. Br J Phys Med. 1954;17:160-2.

30. Grote L, Leissner L, Hedner J, Ulfberg J. A randomized, double-blind, placebo-controlled, multi-center study of intravenous iron sucrose and placebo in the treatment of restless legs syndrome. Mov Disorder. 2009 Jul 30;24(10):1445-52.

31. Brownlie T 4th, Utermohlen V, Hinton PS, Giordano C, Haas JD. Marginal iron deficiency without anaemia impairs aerobic adaptation among previously untrained women. Am J Clin Nutr. 2002;75(4):734-42.

32. Hinton PS, Giordano C, Brownlie T, Haas JD. Iron supplementation improves endurance after training in iron-depleted, nonanemic women. J Appl Physiol. 2000 Mar;88(3):1103-11.

33. Ballin A, Berar M, Rubinstein U, Kleter Y, Hershkovitz A, Meytes D. Iron state in female adolescents. Am J Dis Child. 1992 Jul;146(7):803-5.

34. Vifor Pharma. Effect of intravenous iron versus placebo on exhaustion symptoms in non-anaemic premenopausal women with low ferritin levels 2009.

35. Rangan AM, Blight GD, Binns CW. Iron status and non-specific symptoms of female students. J Am Coll Nutr. 1998 Aug;17(4):351-5.

36. Crichton RR, Danielson BG, Geisser P. Iron therapy with special emphasis on intravenous administration. 4th ed. Germany: Bremen; 2008.

37. Radbruch L, Sabatowski R, Elsner F, Everts J, Mendoza T, Cleeland C. Validation of the German version of the brief fatigue inventory. J Pain Symptom Manage. 2003 May;25(5):449-58.

38. Bates DW, Schmitt W, Buchwald D, Ware NC, Lee J, Thoyer E, et al. Prevalence of fatigue and chronic fatigue syndrome in a primary care practice. Arch Intern Med. 1993 Dec 27;153(24):2759-65.

39. Cathébras PJ, Robbins JM, Kirmayer LJ, Hayton BC. Fatigue in primary care: prevalence, psychiatric comorbidity, illness behavior, and outcome. J Gen Intern Med. 1992 May-Jun;7(3):276-86.

40. Fuhrer R, Wessely S. The epidemiology of fatigue and depression: a French primary-care study. Psychol Med. 1995 Sep;25(5):895-905.

41. Ridsdale L, Evans A, Jerrett W, Mandalia S, Osler K, Vora H. Patients with fatigue in general practice: a prospective study. BMJ. 1993 Jul 10;307(6896):103-6.

42. Piper BF, Dibble SL, Dodd MJ, Weiss MC, Slaughter RE, Paul SM. The revised Piper Fatigue Scale psychometric evaluation in women with breast cancer. Onco Nurs Forum. 1998 May;25(4):677-84.

43. Beshara S, Sörensen J, Lubberink M, Tolmachev V, Långström B, Antoni G. Pharmacokinetics and red cell utilization of $^{52}Fe/^{59}Fe$-labelled iron polymaltose in anaemic patients using positron emission tomography. Br J Haematol. 2003;120:853-9.

44. Kulnigg S, Stoinov S, Simanenkov V, Dudar LV, Karnafel W, Garcia LC, et al. A novel intravenous iron formulation for treatment of anemia in inflammatory bowel disease: the ferric carboxymaltose (FERINJECT®) randomized controlled trial. Am J Gastroenterol. 2007;102:1-11.

45. Van Wyck DB, Martens MG, Seid MH, Baker JB, Mangione A. Intravenous ferric carboxymaltose compared with oral iron in the treatment of postpartum anemia. A randomized controlled trial. Obstet Gynecol. 2007 Aug;110 (2 Pt 1):267-78. Erratum in: Obstet Gynecol. 2007 Aug;110(2 Part 1):267-78.

46. Bodemar G, Kechagias S, Almer S, Danielson BG. Treatment of anaemia in inflammatory bowel disease with iron sucrose. Scand J Gastrolenterol. 2004;39: 454-8.

47. Lindgren S, Wikman O, Befrits R, Blom H, Eriksson A, et al. Intravenous iron sucrose is superior to oral iron sulphate for correcting anaemia and restoring iron store in IBD patients: A randomized, controlled evaluator-blinded multicenter study. Scand J Gastroenterol. 2009;27:1-8.

48. Pharmaceutical Research and Manufacturers of America [homepage online]. 2009. PhRMA principles and guidelines: public disclosure of clinical trial results. Available from: http://www.phrma.org/principles_and_guidelines.

49. International Committee of Medical Journal Editors [homepage on the Internet]. ICMJE guidelines. Available from: http://www.icmje.org.

Patient Information Leaflet **Vifor (International) Inc.**
Regulatory Affairs

Document No:<br>SPC-YV007/YV080/MR/E03	**FERINJECT**	Research & Development<br><br>signed date
Page 1/9		Drug Safety<br><br>signed date
Valid from:<br>20.01.2009		Marketing<br><br>signed date
Replaces document:<br>SPC-YV007/YV080/MR/E02<br>04.07.2008	Regulatory Affairs<br><br>signed date	Quality Assurance<br><br>signed date
Distribution:		

1. NAME OF THE MEDICINAL PRODUCT

FERINJECT 50 mg iron/ml solution for injection/infusion.

2. QUALITATIVE AND QUANTITATIVE COMPOSITION

One millilitre of solution contains 50 mg of iron as ferric carboxymaltose.

Each 2 ml vial contains 100 mg of iron as ferric carboxymaltose.
Each 10 ml vial contains 500 mg of iron as ferric carboxymaltose.

Ferinject contains sodium hydroxide. One millilitre of solution contains up to 0.24 mmol (5.5 mg) sodium, see section 4.2. For a full list of excipients, see section 6.1.

3. PHARMACEUTICAL FORM

Solution for injection/infusion. Dark brown, non-transparent, aqueous solution.

4. CLINICAL PARTICULARS

4.1 Therapeutic indications

FERINJECT is indicated for treatment of iron deficiency when oral iron preparations are ineffective or cannot be used.
The diagnosis must be based on laboratory tests.

4.2 Posology and method of administration

Calculation of the cumulative dose

The adequate cumulative dose of FERINJECT must be calculated for each patient individually and must not be exceeded. For overweight patients, a normal body weight/blood volume relation should be assumed when determining the iron requirement. The dose of FERINJECT is expressed in mg of elemental iron.

The cumulative dose required for Hb restoration and repletion of iron stores is calculated by the following Ganzoni formula:

Cumulative iron deficit [mg] =
body weight [kg] x (target Hb* - actual Hb) [g/dl] x 2.4*** +**

Quality Assurance:
date signed

iron storage depot [mg]****

Factor 2.4 = 0.0034 x 0.07 x 10000;
0.0034: iron content of haemoglobin ≅ 0.34%;
0.07: blood volume ≅ 7% of body weight;

Depot iron for body weight below 35 kg = 15 mg/kg body weight.
Depot iron for body weight 35 kg and above = 500 mg.

For patients ≤ 66 kg: the calculated cumulative dose is to be rounded down to the nearest 100 mg.
For patients > 66 kg: the calculated cumulative dose is to be rounded up to the nearest 100 mg.

Patients may continue to require therapy with FERINJECT at the lowest dose necessary to maintain target levels of haemoglobin, and other laboratory values of iron storage parameters within acceptable limits.

Maximum tolerated single dose
The adequate cumulative dose of FERINJECT must be calculated for each patient individually and must not be exceeded.

Intravenous bolus injection:
FERINJECT may be administered by intravenous injection up to a maximum single dose of 4 ml (200 mg of iron) per day but not more than three times a week.

Intravenous drip infusion:
FERINJECT may be administered by intravenous infusion up to a maximum single dose of 20 ml of FERINJECT (1000 mg of iron) but not exceeding 0.3 ml of FERINJECT (15 mg of iron) per kg body weight or the calculated cumulative dose. Do not administer 20 ml (1000 mg of iron) as an infusion more than once a week.

The use of Ferinject has not been studied in children, and therefore is not recommended in children under 14 years.

Method of administration
FERINJECT must be administered only by the intravenous route: by bolus injection, during a haemodialysis session undiluted directly into the venous limb of the dialyser, or by drip infusion. In case of drip infusion FERINJECT must be diluted only in sterile 0.9% sodium chloride solution as follows:

Dilution plan of FERINJECT for intravenous drip infusion

Quality Assurance:
date signed

FERINJECT	Iron	Maximum amount of sterile 0.9% sodium chloride solution	Minimum administration time
2 to < 4 ml	100 to < 200 mg	50 ml	-
4 to < 10 ml	200 to < 500 mg	100 ml	6 minutes
10 to 20 ml	500 to 1000 mg	250 ml	15 minutes

Note: For stability reasons, dilutions to concentrations less than 2 mg iron/ml are not permissible.

FERINJECT must not to be administered by the intramuscular route.

4.3 Contraindications

The use of FERINJECT is contraindicated in cases of:

- known hypersensitivity to Ferinject or to any of its excipients
- anaemia not attributed to iron deficiency, e.g. other microcytic anaemia
- evidence of iron overload or disturbances in utilisation of iron
- pregnancy in the first trimester

4.4 Special warnings and precautions for use

Parenterally administrered iron preparations can cause hypersensitivity reactions including anaphylactoid reactions, which may be potentially fatal
(see section 5.3). Therefore, facilities for cardio-pulmonary resuscitation must be available.

In patients with liver dysfunction, parenteral iron should only be administered after careful risk/benefit assessment. Parenteral iron administration should be avoided in patients with hepatic dysfunction where iron overload is a precipitating factor, in particular Porphyria Cutanea Tarda (PCT). Careful monitoring of iron status is recommended to avoid iron overload.

Parenteral iron must be used with caution in case of acute or chronic infection, asthma, eczema or atopic allergies. It is recommended that the administration of FERINJECT is stopped in patients with ongoing bacteraemia. In patients with chronic infection a risk/benefit evaluation has to be performed, taking into account the suppression of erythropoiesis.

Caution should be exercised to avoid paravenous leakage when administering FERINJECT. Paravenous leakage of FERINJECT at the injection site may lead to brown discolouration and irritation of the skin. In case of paravenous leakage, the administration of FERINJECT must be stopped immediately.

One millilitre of undiluted FERINJECT contains up to 0.24 mmol (5.5 mg) of sodium. This has to be taken into account in patients on a sodium-controlled diet.

Quality Assurance:
date signed

One millilitre of undiluted FERINJECT contains up to 0.24 mmol (5.5 mg) of sodium. This has to be taken into account in patients on a sodium-controlled diet.

The use of FERINJECT has not been studied in children.

4.5 Interaction with other medicinal products and other forms of interaction

As with all parenteral iron preparations the absorption of oral iron is reduced when administered concomitantly.

4.6 Pregnancy and lactation

Clinical data on pregnant women are not available. A careful risk/benefit evaluation is required before use during pregnancy.

Animal data suggest that iron released from FERINJECT can cross the placental barrier and that its use during pregnancy may influence skeletal development in the fetus.
Clinical studies showed that transfer of iron from FERINJECT to human milk was negligible (≤ 1%). Based on limited data on nursing women it is unlikely that FERINJECT represents a risk to the nursing child.

4.7 Effects on ability to drive and use machines

FERINJECT is unlikely to impair the ability to drive or operate machines.

4.8 Undesirable effects

The most commonly reported ADR is headache, occurring in 3.3% of the patients.

Immune System Disorders
Uncommon (>1/1,000, <1/100): Hypersensitivity including anaphylactoid reactions

Nervous system disorders
Common (>1/100, <1/10): Headache, dizziness
Uncommon (>1/1,000, <1/100): Paraesthesia

Vascular disorders

Quality Assurance:
date signed

Uncommon (>1/1,000, <1/100): Hypotension, flushing

Respiratory, thoracic and mediastinal disorders
Rare (>1/10,000, <1/1,000): Dyspnoea

Gastrointestinal disorders
Common (>1/100, <1/10): Nausea, abdominal pain, constipation, diarrhoea
Uncommon (>1/1,000, <1/100): Dysgeusia, vomiting, dyspepsia, flatulence

Skin and subcutaneous tissue disorders
Common (>1/100, <1/10): Rash
Uncommon (>1/1,000, <1/100): Pruritus, urticaria

Musculoskeletal and connective tissue disorders
Uncommon (>1/1,000, <1/100): Myalgia, back pain, arthralgia

General disorders and administration site conditions
Common (>1/100, <1/10): Injection Site Reactions
Uncommon (>1/1,000, <1/100): Pyrexia, fatigue, chest pain, rigors, malaise, oedema peripheral

Investigations
Common (>1/100, <1/10): Transient blood phosphorus decreased, alanine aminotransferase increased
Uncommon (>1/1,000, <1/100): Aspartate aminostransferase increased, gamma-glutamyltransferase increased, blood lactate dehydrogenase increased

4.9 Overdose

Administration of FERINJECT in quantities exceeding the amount needed to correct iron deficit at the time of administration may lead to accumulation of iron in storage sites eventually leading to haemosiderosis. Monitoring of iron parameters such as serum ferritin and transferrin saturation may assist in recognising iron accumulation.

5. PHARMACOLOGICAL PROPERTIES

5.1 Pharmacodynamic properties

Pharmacotherapeutic group: Iron trivalent, parenteral preparation

ATC Code: B03A C01

Quality Assurance:
date signed

Ferinject solution for injection/infusion contains iron in a stable ferric state as a complex with a carbohydrate polymer designed to release utilisable iron to the iron transport and storage proteins in the body (ferritin and transferrin). Clinical studies showed that the haematological response and the filling of the iron stores was faster after intravenous administration of Ferinject than with orally administered comparators.
Using positron emission tomography (PET) it was demonstrated that red cell utilisation of ^{59}Fe and ^{52}Fe from FERINJECT ranged from 61% to 99%. Patients with iron deficiency showed utilisation of radio-labelled iron of 91% to 99% after 24 days, and patients with renal anaemia showed utilisation of radiolabelled iron of 61% to 84% after 24 days.
One millilitre of undiluted Ferinject contains less than 75 µg aluminium. This should be considered in the treatment of patients undergoing dialysis.

5.2 Pharmacokinetic properties

Using positron emission tomography (PET) it was demonstrated that ^{59}Fe and ^{52}Fe from FERINJECT was rapidly eliminated from the blood, transferred to the bone marrow, and deposited in the liver and spleen.

After administration of a single dose of FERINJECT of 100 to 1000 mg of iron in iron deficient patients, maximum iron levels of 37 µg/ml up to 333 µg/ml after 15 minutes to 1.21 hours respectively are obtained. The volume of the central compartment corresponds well to the volume of the plasma (approximately 3 litres).

The iron injected or infused was rapidly cleared from the plasma, the terminal halflife ranged from 7 to 12 hours, the mean residence time (MRT) from 11 to 18 hours. Renal elimination of iron was negligible.

5.3 Pre-clinical safety data

Pre-clinical data revealed no special hazard for humans based on conventional studies of safety pharmacology, repeat dose toxicity and genotoxicity. Animal studies indicate that iron released from FERINJECT does cross the placental barrier and is excreted in milk. In reproductive toxicology studies using iron replete animals FERINJECT was associated with minor skeletal abnormalities in the fetus. No long-term studies in animals have been performed to evaluate the carcinogenic potential of FERINJECT. No evidence of allergic or immunotoxic potential has been observed. A controlled *in-vivo* test demonstrated no cross-reactivity of FERINJECT with anti-dextran antibodies. No local irritation or intolerance was observed after intravenous administration.

6. PHARMACEUTICAL PARTICULARS

6.1 List of excipients

Quality Assurance:
date signed

Sodium hydroxide (for pH adjustment)
Hydrochloric acid (for pH adjustment)
Water for injections

6.2 Incompatibilities

This medicinal product must not be mixed with other medicinal products than those mentioned in section 6.6.
The compatibility with containers other than polyethylene and glass is not known.

6.3 Shelf-life

Shelf-life of the product as packaged for sale:
3 years.

Shelf-life after first opening of the container:
From a microbiological point of view, preparations for parenteral administration should be used immediately.

Shelf-life after dilution with sterile 0.9% sodium chloride solution:
From a microbiological point of view, preparations for parenteral administration should be used immediately after dilution with sterile 0.9% sodium chloride solution.

6.4. Special precautions for storage

Store in the original package. Do not store above 30 °C. Do not refrigerate or freeze.

6.5. Nature and contents of container

2 ml of solution in a vial (type I glass) with bromobutyl rubber stopper and aluminium cap in pack sizes of 1 and 5 vials.

10 ml of solution in a vial (type I glass) with bromobutyl rubber stopper and aluminium cap in pack sizes of 1 and 5 vials.

6.6 Special precautions for disposal and other handling

Inspect vials visually for sediment and damage before use. Use only those containing sediment-free, homogeneous solution.

Each vial of FERINJECT is intended for single use only. Any unused product or waste material should be disposed of in accordance with local requirements.

Quality Assurance:
date signed

FERINJECT must only be mixed with sterile 0.9% sodium chloride solution. No other intravenous dilution solutions and therapeutic agents should be used, as there is the potential for precipitation and/or interaction. For dilution instructions, see section 4.2.

7. MARKETING AUTHORISATION HOLDER

Vifor France SA
7-13, Bd Paul Emile Victor
92200 Neuilly-sur-Seine
France
Tel. +33 (0)1 41 06 58 90
Fax +33 (0)1 41 06 58 99
e-mail: contact@vifor-france.fr

8. MARKETING AUTHORISATION NUMBER

PL 15240/0002

9. DATE OF FIRST AUTHORISATION/RENEWAL OF THE AUTHORISATION

19.07.2007

10. DATE OF REVISION OF THE TEXT

09.07.2009

Quality Assurance:
date signed

Example Serious Adverse Event (SAE) Form — Total pages of Fax:____

Vifor Pharma Ltd., Fax No.: +41 58 851 86 59 | VIFOR (Int.) ONLY: *Coding number:*______

Vifor Study: PREFER<br>CRO No. :<br>Eudract No.:	Initial Report ❑<br>Follow-up Report ❑<br>Follow-up number:___	Date of Report : ☐☐☐☐☐☐☐☐☐ (dd/mmm/yyyy)

Patient informaon	Year of birth: ☐☐☐☐ (yyyy) If unknown, age at onset: ☐☐ (Years) Gender: Male ❑ Female ❑<br>Patient No.: ☐☐☐☐ ☐☐☐☐<br>Screening No.: Random No.: Weight: ______ kg Height: _______ cm Hb Value _____ mg/dl (at Baseline)
Study Medication	Treatment drug: ❑ Ferinject ❑ Placebo<br>First Application of Study Drug: ☐☐☐☐☐☐☐☐☐ (dd/mmm/yyyy) Last Application of Study Drug: ☐☐☐☐☐☐☐☐☐ (dd/mmm/yyyy)<br>Dilution: __________________ Duration of infusion: _______________<br>Total administered dose: ______________mg<br>Will study drug treatment be continued? ❑ No, If no: Why was study drug stopped? ______________<br>❑ Yes, If yes: Date of next drug administration:______________<br>Next originally planned date of drug administration if it is different from the date above: _________________
Event Verbatim	Serious Adverse Event Term:<br>____
SAE Information	Event start date: ☐☐☐☐☐☐☐☐☐ (dd/mmm/yyyy) Event stop date: ☐☐☐☐☐☐☐☐☐ (dd/mmm/yyyy) OR ongoing ❑
SAE Description	(incl. signs, symptoms, most important reaction(s), diagnosis, relevant tests, relevant lab data)
Concomitant drugs and diseases	Please attach a copy of “Prior and Concomitant Medication”and “Medical and Surgical History” CRF page.<br>-Please continue on next page -

1/2

Action taken	SAE treatment medication given ? ❑ No ❑ Yes<br>If yes, provide medication number(s) of attached CRF: ____<br>Non-drug treatment, specify: ____<br>Withdrawal from study due to event ❑ Yes<br>❑ No
Out-come	❑ Recovered without sequelae ❑ Recovered with sequelae ❑ Not yet recovered ❑ Death ❑ Unknown
SAE category	**❑ Death, date:** [ ][ ][ ][ ][ ][ ][ ][ ][ ] (dd/mmm/yyyy) **Autopsy: ❑ No ❑ Yes**<br>**Cause of death:** ____ **If yes, please attach a copy of the autopsy report**<br>**❑ Life-threatening**<br>**❑ Persistent or significant disability/incapacity**<br>**❑ Inpatient hospitalization…………………………..from** [ ][ ][ ][ ][ ][ ][ ][ ][ ] **to** [ ][ ][ ][ ][ ][ ][ ][ ][ ]<br>**❑ Prolongation of existing inpatient hospitalisation** (dd/mmm/yyyy) (dd/mmm/yyyy)<br>**❑ Congenital anomaly/birth defect**<br>**❑ Important medical event**<br>Premature births will be considered SAEs up to gestational week 35.
Causality Assessment	❑ Unrelated ❑ Related<br>Please describe reason for your opinion: ____<br>____<br>____
Inten-sity	❑ Mild ❑ Moderate ❑ Severe
Reporter Identifier	**Reporter's Name, Profession (speciality):** ____<br>Name & Address of Institution: ____<br>Phone/Fax: ____<br>**Signature of Reporter:** ____ **Date:** [ ][ ][ ][ ][ ][ ][ ][ ][ ] (dd/mmm/yyyy)<br>**Site Number:** [ ][ ][ ] **Investigator's Name (if different form Reporter's Name):**____
VIFOR ONLY	**Date report received:** [ ][ ][ ][ ][ ][ ][ ][ ][ ] (dd/mmm/yyyy)<br>**Name Recipient:** ____<br>**Signature Recipient:** ____

2/2

- *PART I* - **Vifor Pharma Ltd. internal case number:** ________________________

REPORT ON EXPOSURE TO MEDICINES DURING PREGNANCY

➢ DETAILS OF PERSON SUBMITTING REPORT

Name (Title/Level) ____

Address ____

Telephone ______________________________ Fax ___________________________________

➢ DETAILS OF THE PREGNANT WOMAN

Year of birth: ___________________________________Age : _______________________________

Patient-No. : ________________________________ Random. No. ________________________________

First day of the last menstruation: ________________ Calculated birth date: __________________________

Presumed date of conception: ___________________ Pregnancy diagnosed on: ______________________

Body weight: ________________________________ Number of foetuses:___________________________

Number of pregnancies including this one: _________ Outcome of previous pregnancies:________________
(healthy child, miscarriages etc.)

____

____

➢ INFORMATION ON USAGE OF VIFOR COMPOUNDS

Name of the medicine ________________________ Batch number ______________ unknown ☐

Indication __________________________ Dose ____________ Frequency __________________

Start of intake _______________________ Finish of intake ________________________

Further medicines taken during the pregnancy (with exact dates and dosages):

1)______________________________________given from ________________until_____________________

2)______________________________________given from ________________until_____________________

3)______________________________________given from ________________until_____________________

➢ INFORMATION ON THE GENERAL MEDICAL HISTORY OF THE PREGNANT WOMAN

Significant previous diseases or accompanying diseases? No / Yes

If yes, please give details: ____

____

____

Hereditary diseases in the family? No / Yes

- *PART I* -

Vifor Pharma Ltd. internal case number: ________________________

If yes, please give details: ____

____

Sterility treatment? No / Yes

If yes, please give details: ____

____

➢ **INFORMATION ON THE COURSE OF THE PREGNANCY**

Complications during the pregnancy? No / Yes If yes, please give details:

____

____

Presumed causative association with Vifor compound? No / Yes
(Please give opinion, if complications / events have arisen)

___________________________________ ___________________________________
Place, Date Signature/Stamp

- -

- *PART II* -

Vifor (International) internal case number: ________________________

➢ **ADDITIONAL INFORMATION ON OUTCOME OF PREGNANCY**

WELLBEING OF THE NEWBORN / PREMATURELY BORN

Date of birth: ___________________________ Sex: ______________________________

Birth weight:_______________ Size: _______________ APGAR score: ______________________

Healthy newborn / prematurely born? No / Yes (please delete where not applicable)

Complications during labour/birth or after the birth? No / Yes

If yes, please give details: ____

____

Assessment of the newborn / prematurely born: ____

____

Presumed causative association with Vifor compound? No / Yes
(Please give opinion, if complications / events have arisen)

___________________________________ ___________________________________
Place, Date Signature/Stamp

To be written on headed paper

CONSENT TO PARTICIPATE IN A CLINICAL RESEARCH STUDY

**Title of Study:**	**A Multicentre Randomised Placebo-controlled Study to Assess the Efficacy and Safety of a Single Administration of Ferric Carboxymaltose (1,000 mg iron) in Improving Fatigue Symptoms in Iron-deficient Non-anaemic (IDNA) Women of Child Bearing Age**
**Protocol Number:**	**IDNA-2009-01**
**Sponsor:**	**Vifor Pharma Ltd., Flughofstrasse 61 Glattbrugg CH-8152 Switzerland**
**Investigator Name:**	*To be filled in at each site*
**Investigator Address:**	*<add>*
**Contact Number:**	*<add>*
**After Hours Contact:**	*<add>*

General Statement and Background

You are being asked to participate in a research study of a new medicinal product to treat your fatigue/exhaustion symptoms, which is believed to be due to iron deficiency. This document provides you with further information regarding this study. Your participation is voluntary and if you do not wish to participate, there will be no penalty or loss of benefits to which you are otherwise entitled. A decision not to participate will not affect your future care.

The Sponsor of this study is Vifor Pharma Ltd. (hereafter referred to as the Sponsor) and the name of the new medicinal product is Ferinject®, an intravenous preparation of iron.

If there is anything you do not understand or if you would like more information, please ask the study doctor. No study procedures will be done until you have read and signed this form and you will receive a signed and dated copy of this form.

Fatigue is one of the most common complaints in primary care medicine and there is increasing evidence that fatigue and other symptoms affecting your well being and mood may be the result of a lack of iron in your body. If left untreated, deficiency of iron can lead to anaemia, a condition causing changes in your red blood cells, or may cause other symptoms that can appear

before changes in your blood are noticeable. Studies have shown that this condition happens more in women of child bearing age than any other group of people.

In recent years, clinicians have gained valuable experience in recognizing and treating iron deficiency even in the absence of anaemia. However, data from acceptable controlled research studies are not available to provide convincing scientific evidence needed for new medical guidelines for this condition. As a result, this study will assess how safe and how effective a single administration of ferric carboxymaltose (Ferinject® iron) is compared to placebo in improving fatigue symptoms in iron-deficient non anaemic (IDNA) women of child bearing age.

Duration of Study and Number of Participants
If you decide to participate in this study, you will be in this study for up to 10 weeks, including a 2-week screening period and an 8 week observation and assessment period. The study is planned to enroll approximately 288 participating subjects.

Procedures (after you have agreed to participate)
To find out if you are eligible to participate in the study, you will come in for a Screening visit.
At Visit 1 (Screening), the following tests will be done:

- physical examination;
- measurement of heart rate, blood pressure, body temperature and weight;
- samples of blood taken for testing including a blood sample for a pregnancy test at the screening visit.
- self administered questionnaires about your fatigue and general health
- tests to assess your cognitive performance (power of attention, concentration and memory). These tests are computer-based and measure your reaction time to various stimuli. You will complete two training sessions prior to the start of the study. If you are unable to perform three or more of the tests after training you will not be eligible for the study.

At Visit 2 (Baseline, Day 0), Visit 3 (7 days), visit 4 (28 days) and visit 5 (56 days), If you are deemed suitable for the study you will need to return to the study centre 4 more times. The baseline visit will take place within a maximum of 14 days after the initial screening visit. Study medication will be administered at the baseline visit. You will then come to the study centre again at Days 7, 28, and 56. At each of these visits you will have the following assessments completed:

- vital signs (body temperature, blood pressure and heart rate) recorded
- samples of blood and/or urine taken for testing. At Visit 5, some of the blood sample taken at the visit will be used for pregnancy testing.
- Self administered health questionnaires
- Computer based cognitive tests

The study doctor will also ensure you have not taken any caffeine-containing drink or nicotine product at least one hour prior to the fatigue or cognitive function assessments. Each visit will take about 2 hours.

Between visits, you will be provided with a diary to record any important information you want to tell your study doctor during your next visit e.g. discomfort, illness, or changes to medications you are taking.

Study medication
Each study participant will be assigned at random to one of two treatment groups:

Group 1
will be assigned to receive 1000 mg of Ferinject® by intravenous drip infusion. The solution is given by slow injection of into a vein. This procedure is called "infusion" and will last about 15 minutes.

Group 2
will be assigned to receive a placebo, which is a solution with no active drug substance. The infusion will last about 15 minutes.

Neither you nor your doctor can choose which group you belong to. This will be decided by random, i.e., like flipping a coin. You will have a 50% (1:1) chance of being assigned to one or the other group. You will not know which treatment you receive, but your study doctor will know.

Follow-Up
If you have side effects or an illness ongoing at the last study visit, you will receive a phone call 2 weeks later to check how you are feeling.

What will I have to do if I take part?
You will be responsible for attending study visits as arranged with your study doctor for tests and procedures for the duration of your participation in the study. You must also ensure that you follow the specific instructions given to by your doctor.

It is important you tell your doctor about the medications that you are taking, any illness you have, or any recent surgery. You must also tell the study staff if you have to take any other medication during your participation in the study, and inform them straight away of any unusual symptoms or any changes in your health.

Risks
Although special care will be taken into account, taking part in a research project may involve risks that are not known.

The most commonly reported side effect which may be associated to the administration of study treatment is headache. Other reactions that may be associated to the study drug include: allergic-like skin reactions such as rash, redness, swelling and itching, prickling or tingling, hives, local reaction at the injection site. Allergic like reaction are relatively common as they may occur in up to one out of 100 administration. Leakage of the medicinal product at the injection site (if not correctly injected) may lead to brown discolouration and irritation of the skin. In the event of

leakage, the infusion will be stopped immediately. Study doctor will take necessary precautions to minimize the risks and discomfort of such reaction should these occur.

Other possible effects include gastrointestinal symptoms (such as indigestion, belly pain, nausea, vomiting, change in taste, constipation, dyspepsia, diarrhoea and gas), muscle and bone related effects (such as muscle ache, back or joint pains) , trouble breathing, flushing, dizziness, an increase in body temperature, tiredness, chest pain, stiffness, feeling unwell, low blood pressure, and swelling of the feet and ankles. The results of some of your blood tests may show changes.

Blood Collection

All procedures performed during this study are routine. Blood samples will be taken at the scheduled visits from a vein in your arm. Some known risks, although rare, that can be associated with this are pain or soreness, bleeding, burning, dizziness, faint feeling, or a bruise or an infection at the site where the needle was inserted to take the blood. The study nurse and your study doctor will watch you closely both during and after blood is taken. The maximum total amount of blood to be taken for study purposes is approximately 50 mL (about 10 teaspoons). Your blood samples will be tested in laboratory in <<enter country>> and will then be destroyed according to the requirements in <<enter country>>.

Pregnancy

Because you are a woman of child bearing age, you must use a reliable method of contraception during the entire study period and you must inform the study doctor about the method you are using. Your study doctor will advise if the chosen method is acceptable or if additional measures are necessary.

There are no studies performed with Ferinject® in pregnant women. Studies in animals suggest that iron released from Ferinject® can cross the placental barrier and that its use in pregnancy may influence skeletal development in the foetus

If you become pregnant during the study, or think that you have become pregnant, please inform your study doctor immediately. Follow-up information regarding the outcome of the pregnancy and any issues of the infant will be required up to 3 months after birth.

What happens if you need to see another doctor or go to hospital?

If you need to see another doctor, or if you are admitted to another hospital, it is very important that you (or, if you wish,someone in your family) tell the doctor who treats you that you are taking part in this study. If necessary, he will call your study doctor. You should also tell your study doctor if you had to see another doctor, or be admitted to hospital.

Prohibited Medications

The following medication or medication containing any of the following substance should not be taken approximately one month before receiving the study treatment or during the study period:

- Antidepressant
- Antihistamine
- Benzodiazepine or derivative

- Narcotics
- Antibiotics or other antimicrobial
- Gestagens for menstruation repression (unless you have regular menstruation)
- Oral iron preparation or iron containing multivitamins

Your study doctor will review these and any other medications you are taking to determine if it is appropriate for you to take part in this study.

Benefits
You may benefit from this study as your fatigue symptoms may improve or disappear, however, there is no guarantee of this and you may receive no benefit from being in the study. Your participation will however provide information about the study treatment and disease that may benefit others.

The results of all tests and examinations required as part of the study are provided at no cost to you. You will be given close attention from the study staff during the time you are involved in the study. You may receive information about your health from physical examinations and medical tests performed in this study.

Voluntary Participation
Your participation in this study is voluntary. If you decide to participate, you may change your mind and decide to withdraw from the study at any time and for any reason. If you withdraw, you will not suffer any penalty or loss of benefits regarding your future care. If you decide to withdraw from this study, please notify _______________ *(insert contact name and number).* If you withdraw from the study you will be asked to attend a final study visit. It is very important that you attend this final visit for your own health and safety.

In case new information relating to the study medication becomes available that may affect your willingness to continue participation in this study, you will be informed by your study doctor in a timely manner. You will have an opportunity to discuss the new information with your doctor and consider whether or not you want to continue in the study. If you decide to continue in the study, you may be asked to sign an updated Informed Consent form.

Alternative Treatments
There are other treatments available to correct your iron deficiency, including other formulations of iron replacement. Please talk to your study doctor about other options and their potential risks and benefits.

Study-Related Injuries

If you have an injury or illness from the study drug or study-related procedures, you will be given appropriate medical treatment necessary to help you recover from the injury or illness. The medical management of the injury or illness will be provided at no cost to you. The agreement to provide medical treatment at no cost does not include treatment for any injury or illness you experience during the study that is not the result of the study drug or study-related procedures.

If you experience any unexpected symptoms or injury, and if emergency medical treatment is required, please report immediately to:

________________________________ *(Site to insert contact name and number)*.

Subject Insurance *<country specific changes necessary>*
The Sponsor of the study will compensate you if you are injured from the study drug or study-related procedures. For that reason the Sponsor has taken out insurance at the *Insurance Company (name to be added)*. Should you undergo any health problem or other damages during or after the study, contact your study doctor *(Name)*. It is your obligation to immediately notify your study doctor of any such events.

The Sponsor should compensate you if the harm results from procedures that were carried out properly. This means procedures done in the way described in documents given to your doctor about the conduct of this study. This compensation process will not happen if the injury is due to any procedure not carried out properly. Your legal rights to claim compensation for negligence are not affected.

In the case of a claim, the treating doctor and the insurance company must be provided with all information that they may request.

Reasons for Compulsory Study Termination
Your study doctor can withdraw you from the study at any time without your consent if it is in your best interest, or if you are unable to comply with the study procedures (e.g. if you do not attend visits or take your study medication).

In addition, the Sponsor, the Ethics Committee (people who protect subjects' rights), or the relevant Regulatory Authority may withdraw you from the study at any time.

The Sponsor may stop the study at any time for any reason, including but not limited to medical safety, and all subjects will be withdrawn.

If you withdraw voluntarily or are withdrawn involuntarily after the start of study treatment, you will be asked to have the tests and examinations required at the final visit. You have the right to refuse these tests and examinations.

Financial Compensation

There will be no cost to you or your insurance company for the study medication or the study-related procedures and examinations. *<adjust country specific version as per local legal requirements>*

You will not be paid for your participation in this study, but the study drug will be made available to you at no charge during the study, and you will be offered a reasonable compensation to cover any study related procedures including travel costs to and from study visits and other relevant incidentals.

The Sponsor is paying your study doctor and/or *<name of Institution or site>* for the medical procedures and time required during the study.

Confidentiality and Data Protection
Your personal data, and data on diseases, test and examination results will be collected on paper or processed electronically, but only for research purposes in connection with this study.

Direct access to your medical records will be required by authorized representatives of Vifor Pharma to check the accuracy of the study data. Your medical records may also be reviewed by Regulatory Authorities, ethics committees and auditors to check that the study is being carried out correctly. You should understand that by signing the Consent Form you are giving your permission for this to happen.

All data collected will be identified by a code number and your identity will remain unknown. All information which is collected about you that leaves the clinic will have your name and address removed so that you cannot be recognized by it. Your study doctor is responsible for keeping confidentially a code list which makes it possible to link your assigned number to your name. This will be kept in a safe place to ensure that in case of an emergency you can be identified and contacted. The code list will be kept for <insert retention period> years after the end of the study with the study doctor files.

All your study data will be protected in accordance with the European Data Protection legislation. However, your study data (the information with the study code number) may be transferred to a country that may not have the same level of personal protection as the EU. By signing this Consent Form you are giving your permission for this transfer to happen.

You understand that any data or specimens (e.g. blood samples) collected during this study may be used in the research and development of the study medication. The Sponsor does not intend to provide you with ownership or financial benefits that may result from this research or future commercial products.

You have the right to ask the study doctor to see and copy your personal information related to the study.

If you withdraw your consent to share your personal study information, you will be withdrawn from the study and no new data relating to your personal health information will be added to the study records. Although they will stop collecting new information about you, the information they have already collected will still be used for study-related purposes by the study doctor, Sponsor and/or people who work with the Sponsor so that the integrity of the study will be maintained. You may ask for your previously retained identifiable samples to be destroyed to prevent further analysis.

The results of your treatment, including laboratory tests, may be published for scientific purposes but you will not be identified in any report or publication. Your study doctor will be given a copy of the report or publication at the end of the study.

Who Has Reviewed The Study?

An Independent Ethics Committee has reviewed the study and given approval of it.

Whom To Contact With Questions

If you have questions or concerns about the study, or your rights as a participant, or any injury or are unwell, you may contact the Study Doctor or his/her staff at the telephone number below.

Name:_______________________________Telephone: (_____) ________________

If you are calling after hours or on a weekend, you may contact

Name:_______________________________Telephone: (_____) ________________

Consent to Participate Study

Title of Study: **A multicenter randomized placebo-controlled study to assess the efficacy and safety of a single administration of FERINJECT® (1000 mg) in improving fatigue symptoms in iron-deficient non anaemic (IDNA) women of child bearing age**

Protocol Number: **IDNA 2009-01**

I have been given time to read, and have read the subject information about study participation carefully. I have had the opportunity to ask my Study Doctor questions about anything I may not have understood about my participation in this study. All questions have been answered fully.

I understand that my participation in this study is voluntary; I can withdraw from this study at any time without penalty or loss of benefits, and without it affecting my future care.

By signing this form, I agree to take part in this study, and will allow all necessary tests to be done during my participation. I agree that my own doctor may be informed of my participation in this study. I authorize the release of my medical records to the Sponsor, agents of the Sponsor, relevant regulatory authorities and the (insert name of IRB/IEC), while keeping my personal data confidential. I declare that I have been told about the insurance terms and I accept them, as well as my rights and obligations resulting from them. A copy of the Subject Information and this signed and dated Informed Consent Form for the study will be given to me. *(<adjust to local requirements in country specific versions>, e.g. in Germany, data protection needs to be mentioned>)*

A copy of the information sheet and signed consent will be given to you to keep.

____________________	____________________	____________________
Printed Name of Participant	Date	Signature of Participant or Participant's Legally Authorised Representative, if applicable

____

Printed Name of Subject or Subject's Legally Authorised Representative, if applicable

____________________	____________________	______________
Printed Name of Witness	Date	Signature

[Keep this witness line only if applicable for your country]

Statement of Investigator or Designee

I, the undersigned, certify that to the best of my knowledge, the participant/participant's representative signing this consent form had the study fully and carefully explained and clearly understands the nature, risks, and benefits of participation in this research study.

______________________	____________________	____________
Printed Name of Person administering Consent	Signature	Date

These guidelines apply to all study site personnel administrating informed consent to a potential subject *or* their legally acceptable representative, which must be done ***prior*** to conducting any study related functions, including verifying eligibility.

These guidelines are to be used when your site IRB does not provide you with an equivalent documented consent process; or when your site does not have an equivalent written process (like an SOP).

1. Present the potential subject or legally acceptable representative with:
   - The most up-to-date version of the IRB/REB/EC approved informed consent form (ICF)
   - The Subject Information Sheet (PIS) (if any)

2. Explain the following to the potential subject:
   - That the trial involves research
   - The purpose of the trial
   - The trial treatments, procedures to be followed and (if randomized) the probability of each treatment
   - Alternative procedures or treatment that may be available
   - The subject's responsibilities
   - All aspects of the trial which are experimental
   - Reasonably foreseeable risks
   - Reasonably expected benefits
   - Compensation and/or treatment available in the event of trial-related injury
   - Anticipated payment to the subject, and expenses (if applicable)
   - The subject's participation is voluntary; the subject may withdraw consent at anytime. In the USA and whenever possible the withdrawal of consent must be done in writing.
   - Monitor(s), auditor(s) and the IRB/EC and Regulatory Authorities may be allowed direct access to the subjects' medical records.
   - Records identifying the subject will be kept confidential
   - If any information becomes available which may be relevant to the subject's willingness to continue in the trial, he/she should be informed in a timely manner
   - The person(s) to contact for further information regarding the trial and the subject's rights
   - The foreseeable circumstances and/or reasons whereby the subject may be withdrawn from the trial
   - The foreseeable circumstances and/or reasons whereby the trial may be terminated
   - The expected duration of the subject's participation in the trial
   - The approximate number of subjects in the trial

3. Throughout the process, ensure that:

   - The potential subject is not coerced or unduly influenced to participate in the trial

- There is ample opportunity and time for the subject to ask questions and to receive satisfactory answers.
- If the subject (or representative) is unable to read, an impartial witness is present during the entire consent discussion. By signing the consent form, the witness confirms that the trial was fully explained and verbal consent willingly given.
- The consent form is **signed** and **dated** by the subject (or representative), the person explaining the study and the witness (if applicable).
- The subject (or representative) receives a copy of the signed and dated consent form and all other written subject information.

IT IS THE PRINCIPAL INVESTIGATOR'S RESPONSIBILITY TO DOCUMENT THIS PROCESS

4. Tips on documentation of the informed consent process

- There should be a "contextual" statement in the source document to show exactly how and when IC was administered - including the time (even if it is on the ICF).

PATIENT HEALTH QUESTIONNAIRE (PHQ-9)

NAME: ______________________________ **DATE:** ______________

Over the last *2 weeks,* how often have you been bothered by any of the following problems?
(use "✓" to indicate your answer)

	Not at all	Several days	More than half the days	Nearly every day
**1.** Little interest or pleasure in doing things	0	1	2	3
**2.** Feeling down, depressed, or hopeless	0	1	2	3
**3.** Trouble falling or staying asleep, or sleeping too much	0	1	2	3
**4.** Feeling tired or having little energy	0	1	2	3
**5.** Poor appetite or overeating	0	1	2	3
**6.** Feeling bad about yourself—or that you are a failure or have let yourself or your family down	0	1	2	3
**7.** Trouble concentrating on things, such as reading the newspaper or watching television	0	1	2	3
**8.** Moving or speaking so slowly that other people could have noticed. Or the opposite — being so figety or restless that you have been moving around a lot more than usual	0	1	2	3
**9.** Thoughts that you would be better off dead, or of hurting yourself	0	1	2	3
	add columns	______ +	______ +	______

(Healthcare professional: For interpretation of TOTAL, please refer to accompanying scoring card). TOTAL: ______

**10.** If you checked off *any problems,* how *difficult* have these problems made it for you to do your work, take care of things at home, or get along with other people?	Not difficult at all ________<br>Somewhat difficult ________<br>Very difficult ________<br>Extremely difficult ________

Copyright © 1999 Pfizer Inc. All rights reserved. Reproduced with permission. PRIME-MD© is a trademark of Pfizer Inc.

A2663B 10-04-2005

PHQ-9 Patient Depression Questionnaire

For initial diagnosis:

1. Patient completes PHQ-9 Quick Depression Assessment.
2. If there are at least 4 ✓s in the shaded section (including Questions #1 and #2), consider a depressive disorder. Add score to determine severity.

Consider Major Depressive Disorder

- if there are at least 5 ✓s in the shaded section (one of which corresponds to Question #1 or #2)

Consider Other Depressive Disorder

- if there are 2-4 ✓s in the shaded section (one of which corresponds to Question #1 or #2)

Note: Since the questionnaire relies on patient self-report, all responses should be verified by the clinician, and a definitive diagnosis is made on clinical grounds taking into account how well the patient understood the questionnaire, as well as other relevant information from the patient.
Diagnoses of Major Depressive Disorder or Other Depressive Disorder also require impairment of social, occupational, or other important areas of functioning (Question #10) and ruling out normal bereavement, a history of a Manic Episode (Bipolar Disorder), and a physical disorder, medication, or other drug as the biological cause of the depressive symptoms.

To monitor severity over time for newly diagnosed patients or patients in current treatment for depression:

1. Patients may complete questionnaires at baseline and at regular intervals (eg, every 2 weeks) at home and bring them in at their next appointment for scoring or they may complete the questionnaire during each scheduled appointment.
2. Add up ✓s by column. For every ✓: Several days = 1 More than half the days = 2 Nearly every day = 3
3. Add together column scores to get a TOTAL score.
4. Refer to the accompanying **PHQ-9 Scoring Box** to interpret the TOTAL score.
5. Results may be included in patient files to assist you in setting up a treatment goal, determining degree of response, as well as guiding treatment intervention.

Scoring: add up all checked boxes on PHQ-9

For every ✓ Not at all = 0; Several days = 1;
More than half the days = 2; Nearly every day = 3

Interpretation of Total Score

1-4	Minimal depression
5-9	Mild depression
10-14	Moderate depression
15-19	Moderately severe depression
20-27	Severe depression

PHQ9 Copyright © Pfizer Inc. All rights reserved. Reproduced with permission. PRIME-MD ® is a trademark of Pfizer Inc.

A2662B 10-04-2005

Date:______________ Qualifying Assessment

ID

PIPER FATIGUE SCALE (PFS)

Directions: Many individuals can experience a sense of unusual or excessive tiredness whenever they become ill, receive treatment, or recover from their illness/treatment. This unusual sense of tiredness is not usually relieved by either a good night's sleep or by rest. Some call this symptom "fatigue" to distinguish it from the usual sense of tiredness.

For each of the following questions, please fill in the space provided for that response that best describes the fatigue you are experiencing now or for today. Please make every effort to answer each question to the best of your ability. If you are not experiencing fatigue now or for today, fill in the circle indicating "0" for your response. Thank you very much!

1. How long have you been feeling fatigue? (Check one response only).

   1. not feeling fatigue
   2. minutes
   3. hours
   4. days
   5. weeks
   6. months
   7. other (Please describe) ____

2. To what degree is the fatigue you are feeling now causing you distress?

No Distress A Great Deal

1 2 3 4 5 6 7 8 9 10

3. To what degree is the fatigue you are feeling now interfering with your ability to complete your work or school activities?

None A Great Deal

1 2 3 4 5 6 7 8 9 10

4. To what degree is the fatigue you are feeling now interfering with your ability to socialize with your friends?

None A Great Deal

1 2 3 4 5 6 7 8 9 10

5. To what degree is the fatigue you are feeling now interfering with your ability to engage in sexual activity?

None								A Great Deal	
1	2	3	4	5	6	7	8	9	10

6. Overall, how much is the fatigue which you are now experiencing interfering with your ability to engage in the kind of activities you enjoy doing?

None								A Great Deal	
1	2	3	4	5	6	7	8	9	10

7. How would you describe the degree of intensity or severity of the fatigue which you are experiencing now?

Mild								Severe	
1	2	3	4	5	6	7	8	9	10

8. To what degree would you describe the fatigue which you are experiencing now as being?

Pleasant								Unpleasant	
1	2	3	4	5	6	7	8	9	10

9. To what degree would you describe the fatigue which you are experiencing now as being?

Agreeable								Disagreeable	
1	2	3	4	5	6	7	8	9	10

10. To what degree would you describe the fatigue which you are experiencing now as being?

Protective								Destructive	
1	2	3	4	5	6	7	8	9	10

11. To what degree would you describe the fatigue which you are experiencing now as being?

Positive								Negative	
1	2	3	4	5	6	7	8	9	10

12. To what degree would you describe the fatigue which you are experiencing now as being:

Normal									Abnormal
1	2	3	4	5	6	7	8	9	10

13. To what degree are you now feeling:

Strong									Weak
1	2	3	4	5	6	7	8	9	10

14. To what degree are you now feeling:

Awake									Sleepy
1	2	3	4	5	6	7	8	9	10

15. To what degree are you now feeling:

Lively									Listless
1	2	3	4	5	6	7	8	9	10

16. To what degree are you now feeling:

Refreshed									Tired
1	2	3	4	5	6	7	8	9	10

17. To what degree are you now feeling:

Energetic									Unenergetic
1	2	3	4	5	6	7	8	9	10

18. To what degree are you now feeling:

Patient									Impatient
1	2	3	4	5	6	7	8	9	10

19. To what degree are you now feeling:

Relaxed								A Great Deal	
1	2	3	4	5	6	7	8	9	10

20. To what degree are you now feeling:

Exhilarated									Depressed
1	2	3	4	5	6	7	8	9	10

21. To what degree are you now feeling:

Able to Concentrate									Unable to Concentrate
1	2	3	4	5	6	7	8	9	10

22. To what degree are you now feeling:

Able to Remember									Unable to Remember
1	2	3	4	5	6	7	8	9	10

23. To what degree are you now feeling:

Able to Think Clearly									Unable to Think Clearly
1	2	3	4	5	6	7	8	9	10

24. Overall, what do you believe is *most* directly contributing to or causing your fatigue?
____
____
____

25. Overall, the *best* thing you have found to relieve your fatigue is: ______________________
____
____
____

26. Is there anything else you would like to add that would describe your fatigue better to us?
____
____
____

27. Are you experiencing any other symptoms right now? ______________________________
____
____
____

Scoring Piper Fatigue Scale (PFS) Survey Results:

PFS current format and scoring instructions:

1. The PFS in its current form is composed of 22 numerically scaled, "0" to "10" items that measure four dimensions of subjective fatigue: behavioral/severity (6 items; # 2-7); affective meaning (5 items: # 8-12); sensory (5 items: # 13-17); and cognitive/mood (6 items: # 18-23). These 22 items are used to calculate the four sub-scale/dimensional scores and the total fatigue scores.
2. Five additional items (# 1 and # 24-27) are not used to calculate subscale or total fatigue scores but are recommended to be kept on the scale as these items furnish rich, qualitative data. Item # 1, in particular gives a categorical way in which to assess the duration of the respondent's fatigue.
3. To score the PFS, add the items contained on each specific subscale together and divide by the number of items on that subscale. This will give you a subscale score that remains on the same "0" to "10"numeric scale. Should you have missing item data, and the respondent has answered at least 75%-80% of the remaining items on that particular subscale, calculate the subscale mean score based on the number of items answered, and substitute that mean value for the missing item score (mean-item substitution).
4. Recalculate the subscale score. To calculate the total fatigue score, add the 22-item scores together and divide by 22 in order to keep the score on the same numeric "0" to "10" scale.[1]

Severity Codes:

**0**	**NONE**
**1-3**	**MILD**
**4-6**	**MODERATE**
**7-10**	**SEVERE**

1. Piper BF, Dibble SL, Dodd MJ, Weiss MC, Slaughter RE, Paul SM. The revised Piper Fatigue Scale: psychometric evaluation in women with breast cancer. Oncol Nurs Forum. 1998 May;25(4):677-84.

Your Health and Well-Being

This survey asks for your views about your health. This information will help keep track of how you feel and how well you are able to do your usual activities. ***Thank you for completing this survey!***

For each of the following questions, please mark an ☒ in the one box that best describes your answer.

1. In general, would you say your health is:

Excellent	Very good	Good	Fair	Poor
☐ 1	☐ 2	☐ 3	☐ 4	☐ 5

2. The following questions are about activities you might do during a typical day. Does your health now limit you in these activities? If so, how much?

	Yes, limited a lot	Yes, limited a little	No, not limited at all
a Moderate activities, such as moving a table, pushing a vacuum cleaner, bowling, or playing golf	☐ 1	☐ 2	☐ 3
b Climbing several flights of stairs	☐ 1	☐ 2	☐ 3

SF-12v2™ Health Survey © 1994, 2002 by QualityMetric Incorporated and Medical Outcomes Trust. All Rights Reserved.
SF-12® a registered trademark of Medical Outcomes Trust.
(SF12v2 Acute, US Version 2.0)

SAMPLE

3. **During the <u>past week</u>, how much of the time have you had any of the following problems with your work or other regular daily activities <u>as a result of your physical health</u>?**

	All of the time	Most of the time	Some of the time	A little of the time	None of the time
a Accomplished less than you would like	☐ 1	☐ 2	☐ 3	☐ 4	☐ 5
b Were limited in the kind of work or other activities	☐ 1	☐ 2	☐ 3	☐ 4	☐ 5

4. **During the <u>past week</u>, how much of the time have you had any of the following problems with your work or other regular daily activities <u>as a result of any emotional problems</u> (such as feeling depressed or anxious)?**

	All of the time	Most of the time	Some of the time	A little of the time	None of the time
a Accomplished less than you would like	☐ 1	☐ 2	☐ 3	☐ 4	☐ 5
b Did work or other activities less carefully than usual	☐ 1	☐ 2	☐ 3	☐ 4	☐ 5

5. **During the <u>past week</u>, how much did <u>pain</u> interfere with your normal work (including both work outside the home and housework)?**

Not at all	A little bit	Moderately	Quite a bit	Extremely
☐ 1	☐ 2	☐ 3	☐ 4	☐ 5

SAMPLE

SF-12v2™ Health Survey © 1994, 2002 by QualityMetric Incorporated and Medical Outcomes Trust. All Rights Reserved.
SF-12® a registered trademark of Medical Outcomes Trust.
(SF12v2 Acute, US Version 2.0)

6. **These questions are about how you feel and how things have been with you __during the past week__. For each question, please give the one answer that comes closest to the way you have been feeling. How much of the time during the __past week__...**

	All of the time	Most of the time	Some of the time	A little of the time	None of the time
a Have you felt calm and peaceful?	☐ 1	☐ 2	☐ 3	☐ 4	☐ 5
b Did you have a lot of energy?	☐ 1	☐ 2	☐ 3	☐ 4	☐ 5
c Have you felt downhearted and depressed?	☐ 1	☐ 2	☐ 3	☐ 4	☐ 5

7. **During the __past week__, how much of the time has your __physical health or emotional problems__ interfered with your social activities (like visiting friends, relatives, etc.)?**

All of the time	Most of the time	Some of the time	A little of the time	None of the time
☐ 1	☐ 2	☐ 3	☐ 4	☐ 5

Thank you for completing these questions!

SAMPLE

SF-12v2™ Health Survey © 1994, 2002 by QualityMetric Incorporated and Medical Outcomes Trust. All Rights Reserved.
SF-12® a registered trademark of Medical Outcomes Trust.
(SF12v2 Acute, US Version 2.0)

Essential diagnostic criteria for RLS[1].

All of the following 4 criteria are necessary for a diagnosis.

1. An urge to move the legs, usually accompanied or caused by uncomfortable and unpleasant sensations in the legs.

2. The urge to move or unpleasant sensations begin or worsen during periods of rest or inactivity such as lying or sitting.
3. The urge to move or unpleasant sensations are partially or totally relieved by movement, such as walking or stretching, at least as long as the activity continues.
4. The urge to move or unpleasant sensations are worse in the evening or night than during the day or only occur in the evening or night.

---

[1] Allen RP, Picchietti D, Hening WA, Trenkwalder C, Walters AS, Montplaisir J. Restless legs syndrome: diagnostic criteria, special considerations, and epidemiology. *Sleep Med* 2003; 4: 101-19.

Restless Legs Syndrome Rating Scale

Have the patient rate his/her symptoms for the following ten questions.
The patient and not the examiner should make the ratings, but the examiner should be available to clarify any misunderstandings the patient may have about the questions.
The examiner should mark the patient's answers on the form.

In the past week…
(1) Overall, how would you rate the RLS discomfort in your legs or arms?
- _ (4) Very severe
- _ (3) Severe
- _ (2) Moderate
- _ (1) Mild
- _ (0) None

In the past week…
(2) Overall, how would you rate the need to move around because of your RLS symptoms?
- _ (4) Very severe
- _ (3) Severe
- _ (2) Moderate
- _ (1) Mild
- _ (0) None

In the past week…
(3) Overall, how much relief of your RLS arm or leg discomfort did you get from moving around?
- _ (4) No relief
- _ (3) Mild relief
- _ (2) Moderate relief
- _ (1) Either complete or almost complete relief
- _ (0) No RLS symptoms to be relieved

In the past week…
(4) How severe was your sleep disturbance due to your RLS symptoms?
- _ (4) Very severe
- _ (3) Severe
- _ (2) Moderate
- _ (1) Mild
- _ (0) None

In the past week…
(5) How severe was your tiredness or sleepiness during the day due to your RLS symptoms?
- _ (4) Very severe
- _ (3) Severe
- _ (2) Moderate
- _ (1) Mild
- _ (0) None

In the past week…
(6) How severe was your RLS as a whole?
- _ (4) Very severe
- _ (3) Severe
- _ (2) Moderate
- _ (1) Mild
- _ (0) None

In the past week…
(7) How often did you get RLS symptoms?
- _ (4) Very often (6 to 7 days in 1 week)
- _ (3) Often (4 to 5 days in 1 week)
- _ (2) Sometimes (2 to 3 days in 1 week)
- _ (1) Occasionally (1 day in 1 week)
- _ (0) Never

In the past week…
(8) When you had RLS symptoms, how severe were they on average?
- _ (4) Very severe (8 hours or more per 24 hour)
- _ (3) Severe (3 to 8 hours per 24 hour)
- _ (2) Moderate (1 to 3 hours per 24 hour)
- _ (1) Mild (less than 1 hour per 24 hour)
- _ (0) None

In the past week…
(9) Overall, how severe was the impact of your RLS symptoms on your ability to carry out your daily affairs, for example carrying out a satisfactory family, home, social, school or work life?
- _ (4) Very severe
- _ (3) Severe
- _ (2) Moderate
- _ (1) Mild
- _ (0) None

In the past week…
(10) How severe was your mood disturbance due to your RLS symptoms - for example angry, depressed, sad, anxious or irritable?
- _ (4) Very severe
- _ (3) Severe
- _ (2) Moderate
- _ (1) Mild
- _ (0) None

Sum of scores =

Scoring criteria are: Mild (score 1-10); Moderate (score 11-20); Severe (score 21-30); Very severe (score 31-40)

1. Answers for this IRLS are scored from 4 for the first (top) answer (usually ‘very severe’) to 0 for the last answer (usually none). All items are scored. The sum of the item scores serves as the scale score.

The International Restless Legs Syndrome Study Group holds the copyright for this scale.

COGNITIVE ASSESSMENTS

The CDR Computerised Cognitive Assessment System

A selection of tasks from the CDR computerised cognitive assessment system will be administered, parallel forms of the tests being presented on each testing session. All tasks are computer-controlled, the information being presented on high resolution screens, and the responses recorded via a response module containing two buttons, one marked 'NO' and the other 'YES'. In the word recall tasks the patient writes down the words on a sheet of paper. The test battery takes about 20-25 minutes to perform. The tests are administered in the following order:

- Immediate Word Recall: A list of 15 words is presented on the screen at the rate of 1 every 2 seconds for the patient to remember. The patient is then given 1 minute to recall as many of the words as possible.
- Picture Presentation: A series of 20 pictures is presented on the screen at the rate of 1 every 3 seconds for the patient to remember. No data are recorded from this task.
- Simple Reaction Time: The patient is instructed to press the 'YES' response button as quickly as possible every time the word 'YES' is presented on the screen. Thirty stimuli are presented with a varying inter-stimulus interval.
- Digit Vigilance Task: A target digit is randomly selected and constantly displayed to the right of the screen. A series of digits is then presented in the centre of the screen at the rate of 150 per minute and the patient is required to press the 'YES' button as quickly as possible every time the digit in the series matched the target digit. There are 45 targets. The task lasts for about 3 minutes.
- Choice Reaction Time: Either the word 'NO' or the word 'YES' is presented on the screen and the patient is instructed to press the corresponding button as quickly as possible. There are 30 trials for which each stimulus word is chosen randomly with equal probability and there is a varying inter-stimulus interval.
- Spatial Working Memory: A picture of a house is presented on the screen with four of its nine windows lit. The patient has to memorise the position of the lit windows. For each of the 36 subsequent presentations of the house, the patient is required to decide whether or not the one window that was lit was also lit in the original presentation. The patient responds by pressing the 'YES' or 'NO' response button as appropriate.
- Numeric Working Memory: A series of five digits is presented for the patient to hold in memory. This is followed by a series of 30 probe digits for each of which the patient has to decide whether or not it was in the original series and press the 'YES' or 'NO' response button as appropriate.
- Delayed Word Recall: The patient is again given 1 minute to recall as many of the words as possible.
- Word Recognition: The original words plus 15 distractor words are presented one at a time in a randomised order. For each word the patient is required to indicate whether or not the patient recognises it as being from the original list of words by pressing the 'YES' or 'NO' button as appropriate, as quickly as possible.

- Picture Recognition: The original pictures plus 20 distractor pictures are presented one at a time in a randomised order. For each picture the patient has to indicate whether or not the patient recognises it as being from the original series by pressing the 'YES' or 'NO' button as appropriate.

Additional Assessments

- Bond-Lader VAS of Mood and Alertness (Bond and Lader, 1974): This questionnaire of 16 analogue scales derives three factors that assess change in Self-rated Alertness, Self-rated Calmness and Self-rated Contentment. It has proven sensitivity to a wide range of compounds. A computerised version of this test is employed, with the patient using the computer mouse.

CDR TESTING SCHEDULE

Training

Training on the CDR system and the Visual Analogue Scales will take place prior to the first day of dosing of the trial in order to ensure an optimal level of performance for the baseline assessment on the first study day. Training helps overcome initial test anxiety, familiarises the patients with the procedures, enables the development of strategies for task performance and overcomes any initial practice effects. Two training sessions will be completed by each patient prior to the start of the study. If a patient is unable to perform three or more of the CDR tasks to the specified level (age-matched normative data) then the patient shall be excluded from the study.

Study Days

The CDR system test battery will be administered at pre-dose on Day 1, Day 8, Day 28 and Day 56 (or at the early termination visit).

Patients should not smoke, or drink caffeine containing drinks for 1 hour prior to testing.

DEPENDENT VARIABLES

CDR Assessments

**Task**	**Major Measure**	**Supportive Measure**
Attentional Tasks		
Simple Reaction Time	Speed (msec)	
Digit Vigilance	Speed (msec)<br>Targets detected (%)	False Alarms (#)
Choice Reaction Time	Speed (msec)	Accuracy (%)
Working Memory Tasks		
Numeric Working Memory	Sensitivity (SI)<br>Speed (msec)	

Spatial Working Memory	Sensitivity (SI)<br>Speed (msec)	
Episodic Secondary Memory Tasks		
Immediate Word Recall	Words correctly recalled (%)	Errors (#)
Delayed Word Recall	Words correctly recalled (%)	Errors (#)
Word Recognition	Sensitivity (SI)<br>Speed (msec)	
Picture Recognition	Sensitivity (SI)<br>Speed (msec)	

Major measures are measures which, if affected, reflect upon the overall efficiency of the performance of the task. Supportive measures are those which, if affected by study treatment, will not in themselves reflect on the actual ability to perform the task, but will be important in modulating the interpretation of any changes in the major measures. The overall procedure is therefore to determine whether any changes occurred for any of the major measures. If any changes occurred, an interpretation of the consequence for task performance is made taking into consideration changes in any other measures of that task.

In all tasks the overall speed scores are derived from correct responses.

A signal detection theory index of sensitivity is used for the working memory and recognition tasks to provide an overall measure of quality of recognition. This sensitivity index (SI) is calculated from formulae presented by Frey and Colliver (1973) and combines the accuracy scores to the original as well as the novel (distractor) information. Thus the sensitivity index, by combining the ability to identify previously presented items and to correctly reject items which were not previously presented, represents the overall ability of the patient to recognise (or be sensitive to) the task information. For all practical purposes this score ranges from 0 to 1. A score of 1 represents perfect recognition performance - all of the previously presented items are correctly identified, and all distractors are correctly rejected as being novel. At the other extreme, a score of zero represents chance performance, or total insensitivity to the task information, i.e. the balance of the responses to the original and distractor items yields no evidence that the patient is able to discriminate between them. In practice, negative sensitivity scores are sometimes obtained with poor performance.

FACTOR SCORES

In addition to the analysis of the individual measures, five combined scores based on the outcome of factor analysis are used to further characterise the data (Wesnes et al, 2000b).

**Factor Score**	**CDR Task Measure**
Power of Attention	Simple Reaction Time
	Choice Reaction Time
	Digit Vigilance - Speed of Detections

Continuity of Attention	Choice Reaction Time – Accuracy
	Digit Vigilance – Correct Detections
	Digit Vigilance – False Alarms
Quality of Working Memory	Numeric Working Memory – Sensitivity Index
	Spatial Working Memory – Sensitivity Index
Quality of Episodic Secondary Memory	Immediate Word Recall – Accuracy & Errors
	Delayed Word Recall – Accuracy & Errors
	Word Recognition – Sensitivity Index
	Picture Recognition – Sensitivity Index
Speed of Memory	Speed of Numeric Working Memory
	Speed of Spatial Working Memory
	Speed of Word Recognition
	Speed of Picture Recognition

Power of Attention

Simple Reaction Time
Choice Reaction Time
Speed of Detections in Digit Vigilance task

The speed scores from the three attentional tasks all load strongly and in the same direction on this factor. In such tasks, speed reflects the intensity of concentration at that particular moment, the faster the response, the more processes that are being brought to bear upon the task. In everyday terms, this reflects high levels of effortful concentration, for example, 'straining to hear a sound', 'peering closely at an object of great interest', 'listening intently to someone speaking', 'watching a dramatic part of a film' and so on. These three reaction time scores, which are all close in overall magnitude, are summed to create the factor score for this item. The summation technique is a simple addition of the three scores.

Continuity of Attention

Accuracy of responding in Choice Reaction Time task
Percent Target Detection in Digit Vigilance task
False Alarms in Digit Vigilance task

Here the scores all reflect the ability of the patient to sustain concentration. The overall ability to detect the targets in the three-minute Digit Vigilance task is a clear example. The ability of the patient in the Choice Reaction Time task to make the correct decision over each of the 50 trials is also easy to incorporate within this framework. In the Digit Vigilance task again, the number of times that concentration lapses is reflected by the times the patient mistakes a non-target for a target item, and presses the button in error. Note also, and very importantly, that this loads negatively (or in the inverse direction) on this factor, compared to the first two items; thus for the accuracy scores, the higher the better, whereas for false alarms, the fewer which are made, the better the performance. To combine these scores, the following was done. For Choice Reaction Time, the number of correct responses out of 50 was identified. For Digit Vigilance, the total number of targets correctly identified (out of 45) was

calculated. The number of false alarms in the Digit Vigilance task was subtracted directly from the total targets correctly detected. These two scores were summed. As all scores are of comparable magnitude, this method yields the fairest overall combination.

Quality of Working Memory

Numeric Working Memory Sensitivity
Spatial Working Memory Sensitivity

These two quite different tasks tap the two mechanisms we have available for keeping information in our minds without previously having learnt it. In the first, the Numeric Working Memory task, the score reflects how well patients can maintain a string of digits in working memory by continual rehearsal without forgetting any of them. In the second, the Spatial Working Memory task, the score reflects how well patients can hold information about the spatial location of information in working memory. In every day terms, this factor reflects the ability to remember someone's name immediately after being introduced, to remember a list of things to do, a telephone number, or where you have just placed a particular item. The factor score for this measure is obtained by summing the two sensitivity scores. The summation technique is a simple addition of the two scores.

Quality of Episodic Secondary Memory

Immediate Word Recall Accuracy & Errors
Delayed Word Recall Accuracy & Errors
Word Recognition Original and New Stimuli - Accuracy
Picture Recognition Original and New Stimuli - Accuracy

The measures of how well the patients can recall the words in the Immediate and Delayed Word Recall tasks, as well as how well they can correctly recognise the same words, and also correctly recognise the pictures, all load strongly and in the same direction on this factor. This is a unique factor, none of the items load significantly on any other factor. This factor thus reflects the ability to store, hold and retrieve information of an episodic nature (ie an event, a name, an object, a scene, an appointment etc). To derive this factor score the following steps are taken. For the Word Recall tasks, the number of words recalled correctly are adjusted for the number of words falsely recalled. The score for each task is then calculated as a percentage of the 15 words originally presented. For the Word and Picture Recognition tasks, there are two important measures, the number of items correctly recognised, and the number of items which were not previously presented that were also correctly rejected. These scores are averaged to obtain an overall percentage accuracy, and then adjusted for chance responding (50%), to yield a score which reflects the pure recognition ability for the words and pictures. These scores from the four tasks which are all of a comparable magnitude, are then summed to derive the factor score for this measure. The summation technique is a simple addition of the four scores.

Speed of Memory

Numeric Working Memory Speed
Spatial Working Memory Speed
Word Recognition Speed
Picture Recognition Speed

The speed measures from the two working memory tasks and the two recognition tasks all load highly and in the same direction on this factor. This factor reflects the time it takes to correctly decide whether or not an item is held in working memory, or to correctly decide whether or not an item is held in episodic secondary memory. In every day terms, it reflects the time it takes to recall a name, a face, or any other item from episodic secondary memory. For working memory, the equivalent is remembering the next thing you are about to say, what someone just said to you, where you have just placed something, or why you have just walked into a room. The speed scores for these four tasks are summed to form the factor score. The summation technique is a simple addition of the four scores.

Bond-Lader VAS

The sixteen VAS scores are combined, as recommended by Bond and Lader (1974) to form three assessments:

Self-rated Alertness, Self-rated Contentment, Self-rated Calmness.

REPORTING

A report will be prepared to document the outcome of the analysis. The report will contain a summary, an introduction, a description of the methods used, the results and a full discussion and conclusion. Appendices to the report will contain graphs of the data and listings of summary statistics, summary statistics for the difference from baseline data, summary SAS output and raw data.

QUALITY ASSURANCE

All stages of CDR's involvement in this study will be subject to CDR internal quality assurance. Quality control checks and quality assurance will be conducted on system set-up, administration of the system to patients, data processing and preparation of the report.

ARCHIVING

In accordance with GCP, all data and relevant study materials pertaining to the cognitive assessments in this study will be retained in the CDR archive facility for a minimum of fifteen years after submission of the final report. There is no requirement for a copy of the CDR data to remain at the study site.

REFERENCES & RELEVANT PUBLICATIONS

Allain H, Neuman E, Malbezin M, Salzman V, Guez D, Wesnes K, Gandon JM (1997). Bridging study of S12024 in 53 in-patients with Alzheimer's disease. Journal of American the Geriatric Society. 45: 125-126.

Beuzen J-N, Taylor N, Wesnes K, Wood A (1999). A comparison of the effects of olanzapine, haloperidol and placebo on cognitive and psychomotor functions in healthy elderly patients. Journal of Psychopharmacology 13: 152-158.

Bond A, Lader M (1974). The use of analogue scales in rating self-rated feelings. British Journal of Medical Psychology 47: 211-218.

Frey PW, Colliver JA (1973). Sensitivity and responsivity measures for discrimination learning. Learning and Motivation 4: 327-342.

Ebert U, Siepmann M, Oertel R, Wesnes K, Kirch W (1998). Pharmacokinetics and pharmacodynamics of scopolamine after subcutaneous administration. Journal of Clinical Pharmacology 38: 720-726.

Harrington F, Saxby BK, McKeith IG, Wesnes K and Ford GA (2000). Cognitive Performance in Hypertensive and Normotensive Older Subjects. Hypertension 36: 1079-1082.

Holland RL, Wesnes K, Dietrich B (1994). Single dose human pharmacology of umespirone. European Journal of Clinical Pharmacology 46: 461-468.

Kastberg H, Jansen JA, Cole G, Wesnes K (1998). Tiagabine: absence of kinetic or dynamic interactions with ethanol. Drug Metabolism and Drug Interactions 14: 259-273.

Legangneux E, McEwen J, Wesnes KA, Bergougnan L, Miget N, Canal M, L'Heritier C, Pinquier JL, Rosenzweig P (2000). The acute effects of amisulpride (50 mg and 200 mg) and haloperidol 2 mg on cognitive function in healthy elderly patients. Journal of Psychopharmacology 14: 164-171.

McKeith I, Del Ser T, Spano F, Wesnes K, Anand R, Cicin-Sain A, Ferrera R and Spiegel R (2000). Efficacy of rivastigmine in dementia with Lewy bodies: results of a randomised placebo-controlled international study. Lancet 356: 2031-2036.

McNair DM, Lorr M, Droppleman LF. EdITS Manual for the Profile of Mood States. EdITS/Educational and Industrial Testing Service 1992; San Diego, CA 92167.

Preece AW, Iwi G, Davies-Smith A, Wesnes K, Butler S, Lim E, Varey A (1999). Effect of a 915-MHz simulated mobile phone signal on cognitive function in man. International Journal of Radiation Biology 75: 447-456.

Rapeport WG, Williams SA, Muirhead DC, Dewland PM, Tanner T, Wesnes K (1996). Absence of a sertraline mediated effect on the pharmacokinetics and pharmacodynamics of carbamazepine. Journal of Clinical Psychiatry 57: 20 – 23.

van Harten J, Stevens L, Raghoebar M, Holland R, Wesnes K and Cournot A (1992). Fluvoxamine does not interact with alcohol or potentiate alcohol-related impairment of cognitive function. Clinical Pharmacology Therapeutics. 52: 427-435.

Walker MP, Ayre GA, Cummings JL, Wesnes K, McKeith IG, O'Brien JT, Ballard CG (2000). Quantifying fluctuation in Dementia with Lewy Bodies, Alzheimer's disease and vascular dementia. Neurology 54: 1616-1625.

Wesnes K (2000). The value of assessing cognitive function in drug development. Dialogues in Clinical Neuroscience 2: 185-203.

Wesnes KA, Garratt C, Wickens M, Gudgeon A, Oliver S (2000a). Effects of sibutramine alone and with alcohol on cognitive function in healthy patients. British Journal of Clinical Pharmacology 49: 110-117.

Wesnes KA, Ward T, McGinty A, Petrini O (2000b). The memory enhancing effects of a Ginkgo biloba/Panax ginseng combination in healthy middle aged patients. Psychopharmacology 152: 353-361.
